# Fall inducing movable platform (FIMP) for overground trips and slips

**DOI:** 10.1186/s12984-020-00785-0

**Published:** 2020-12-03

**Authors:** Jie Kai Er, Cyril John William Donnelly, Seng Kwee Wee, Wei Tech Ang

**Affiliations:** 1 Nanyang Technological University, Rehabilitation Research Institute of Singapore, 11 Mandalay Road, #14-03, 308232 Singapore, Singapore; 2 Tan Tock Seng Hospital, Centre for Advanced Rehabilitation Therapeutics, 11 Jalan Tan Tock Seng, 308433 Singapore, Singapore

**Keywords:** Balance, Overground walking, Fall inducing platforms, Ankle perturbation, SPM

## Abstract

**Background:**

The study of falls and fall prevention/intervention devices requires the recording of true falls incidence. However, true falls are rare, random, and difficult to collect in real world settings. A system capable of producing falls in an ecologically valid manner will be very helpful in collecting the data necessary to advance our understanding of the neuro and musculoskeletal mechanisms underpinning real-world falls events.

**Methods:**

A fall inducing movable platform (FIMP) was designed to arrest or accelerate a subject’s ankle to induce a trip or slip. The ankle was arrested posteriorly with an electromagnetic brake and accelerated anteriorly with a motor. A power spring was connected in series between the ankle and the brake/motor to allow freedom of movement (system transparency) when a fall is not being induced. A gait phase detection algorithm was also created to enable precise activation of the fall inducing mechanisms. Statistical Parametric Mapping (SPM1D) and one-way repeated measure ANOVA were used to evaluate the ability of the FIMP to induce a trip or slip.

**Results:**

During FIMP induced trips, the brake activates at the terminal swing or mid swing gait phase to induce the lowering or skipping strategies, respectively. For the lowering strategy, the characteristic leg lowering and subsequent contralateral leg swing was seen in all subjects. Likewise, for the skipping strategy, all subjects skipped forward on the perturbed leg. Slip was induced by FIMP by using a motor to impart unwanted forward acceleration to the ankle with the help of friction-reducing ground sliding sheets. Joint stiffening was observed during the slips, and subjects universally adopted the *surfing* strategy after the initial slip.

**Conclusion:**

The results indicate that FIMP can induce ecologically valid falls under controlled laboratory conditions. The use of SPM1D in conjunction with FIMP allows for the time varying statistical quantification of trip and slip reactive kinematics events. With future research, fall recovery anomalies in subjects can now also be systematically evaluated through the assessment of other neuromuscular variables such as joint forces, muscle activation and muscle forces.

## Background

Global fall incidence in elderly population(s) (age $\ge$ 65) has an annual mean rate of approximately 30% [1], with the rate doubling for individuals above 75 years old [2]. The importance of fall related solutions increases as the world population ages. However, the rarity and variability of real-world falls greatly impedes the progression of falls related research. It is impracticable to request the elderly to wear motion capture sensors all year round only to capture one instance of fall. Hence, systems capable of inducing falls in safe controlled environments are essential to advancing our understanding of the neuro and musculoskeletal mechanisms underpinning falls events.

Trips and slips are the focus of this work as they represent the majority of externally induced falls in real world settings [3–5]. Trips induce different recovery strategies depending on when in the gait phase an individual is perturbed. An elevating strategy is utilised when the swing leg encounters an easy to overcome perturbation during the early to mid swing gait phase [6, 7]. If the obstacle or perturbation is sufficiently large, a skipping strategy is utilised [8]. This is due to the perturbed leg being arrested from forward motion, requiring the contralateral leg to skip forward to reinstate a suitable base of support and regain stability. A lowering strategy is used during late-swing trips, where the perturbed leg lowers immediately after perturbation and an additional step is taken to clear the obstacle [6, 7].

Backwards falls are normally caused by slips that occur during the initial stance phase. Normally, the stance leg acts as a resistance force (foot to ground friction) during the initial stance phase that converts the forward momentum of the body into angular momentum of the upper body relative to the lower body. This conversion is possible because of the ankle rotating joint and the superiorly located body Center of Mass (CoM) to the resistive force. When there is a lack of friction, the resistive force is no longer sufficient to generate upper body angular momentum, and the entire body slides forward, creating a slip. Recovery strategies following a slip sees the slipped foot immediately adopting a flat-footed configuration relative to the ground and the contralateral leg is placed behind the CoM to provide a recovery moment. Subsequent walking gait following the initial slip response will follow the *surfing* strategy, with the swing foot sliding forward instead of stepping off quickly during the swing phase [9]. This is done to increase the contact area between the foot and the ground, which is though to increase frictional forces.

Trips and slips have often been studied and induced separately as their fall and recovery mechanisms are vastly different. Trips are commonly induced by an obstacle while walking on an instrumented treadmill [10–12]. These treadmill systems allow for precise and accurate velocity control that conventional overground walking systems are generally not able to replicate. Obstacles and perturbations can also be rendered easily as many mechanisms can be hidden around and under the instrumented treadmill systems. Though there are obvious benefits for the use of instrumented treadmill systems within the falls literature, it is widely known that an individual’s gait pattern changes when walking on a treadmill versus overground. Differences in an individual’s kinematics [13–15], joint moments and muscular activation [15–17] have been well documented. Additionally, control of the treadmill after fall onset is critical to replicate true fall dynamics. The treadmill must travel exactly to the speed of the recovery limb to prevent artificially widening or narrowing their base of support (BoS).

Another type of trip induction system uses overground walking to generate more realistic real-world type falls. This type of systems need to account for the subject’s changing linear position during walking gait. Multiple hidden obstacles are built to induce trips along a fixed pathway [6, 18–20]. Since different gait phases induce different recovery strategies, these obstacles have to be densely packed to synchronise the simulated trip with the correct gait phase [6, 9]. The number and size of these fall inducing mechanisms makes this an expensive experimental technique which may not be practical for many laboratories globally. A more cost-effective approach is to develop a localised brake and motor system in the place of multiple ground-based obstacles to induce falls over a distance [21]. The primary drawback of this system is the need for an overhanging railing harness system for safety, limiting its use to designated locations. The overhanging railing harness system also has high inertia which can alter the gait mechanics of the subject under investigation.

Slip experiments commonly depend on a split-belt treadmill [22, 23] or a motorised floor plate [24] to provide the sudden gain in acceleration during a slip. However, the limited actuation distance of these devices means that slip only occurs over a short distance. Once the motorised plate or treadmill stops, the subject can generally regain stability immediately, unlike real-world slips in which velocity decreases slowly over a slippery surface. Even if the deceleration of the motorised plate or treadmill is controlled, it is difficult to match the intended joint kinematics. Furthermore, as previously mentioned, treadmill-walking can change the gait mechanics of the subject, arguably preventing the observation of a true transition from walking to slipping. A sliding plate [25] is better at replicating true slip scenarios, but its limited sliding distance is an important constrain. Another method of inducing slip relies on sliding over a slippery surface [26–28]. This method replicates true slip scenarios, but similar to a trip, they are also constrained by a high inertia overhanging harness which can prevent the observation of a true transition from walking to slipping.

To the best of our knowledge, there exists one fall inducing robot for overground walking that allows for changes in heading angle, does not impose constraints on the walking path and is not constrained by a high inertia overhanging harness [29]. This robot induces fall-like imbalance through perturbation to the pelvis. However, this method of fall induction bypasses the lower limbs’ reactive responses that are present in real-world fall scenarios. The unwanted dynamics of the lower limbs caused by obstacles and slippery surfaces are disregarded, preventing the reproduction of ecologically valid fall recovery strategies. For example, a leg that encountered an obstacle during a real trip will experience sudden deceleration and the user will require time to overcome the unwanted dynamics and widen their BoS. Instead of leg deceleration during a trip, the forward pelvis perturbation from the robot may unintentionally assist the subject to widen their BoS, resulting in improved stability.

**Fig. 1:**
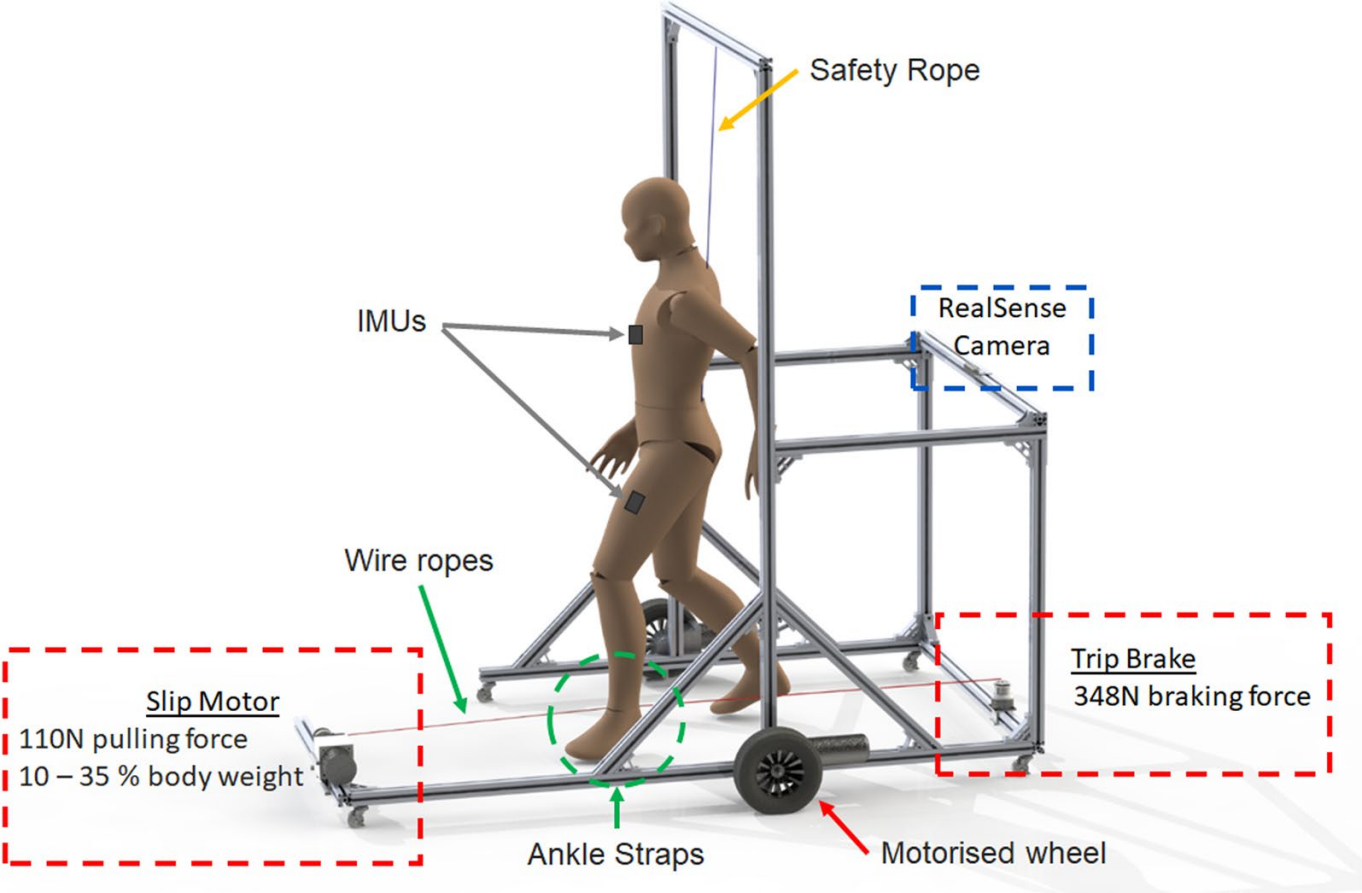
The Fall Inducing Movable Platform (FIMP). Its frame was constructed from 40 $\times$ 40 mm aluminium profiles. A brake is used to induce trips, while a DC motor is used for slips. Wire ropes (illustrated as thin red lines) connect the mechanisms to the left leg at the ankle (green dashed circle). A power spring connected in series between the ankle and the brake or motor provided the freedom of movement required to walk normally. A RealSense camera mounted at the back of the platform enables subject following. The safety rope attached to the overhead crossbeam of the system prevents fall impact while imposing minimal inertial load on the subject during walking

The purpose of this research is to develop a Fall Inducing Movable Platform (FIMP) for realistic fall induction (Fig. 1). The FIMP should have the following characteristics:

- Usable on relatively level ground without space constraints.
- Allows changes in heading angle, velocity and gait patterns.
- Minimises mechanical inertia from the safety harness system worn by the subject.
- Induces ecologically valid falls via ankle perturbations.
- Capable of inducing both a trip and slip.
- Capable of producing random, unexpected perturbations.

FIMP acts as a platform for the mounting and integration of sensors, actuators and processing units required to perform ecologically valid falls.

Another shortcoming of prior research in this fall-related field is in the analysis of the time varying human motion data (i.e., kinematic, kinetics, muscle force). Time varying or continuum human movement data are not analysed as a time series, but as numerous discrete, or zero-dimensional (0D), data points, such that only 0D statistics such as the maxima, minima, mean, and median can be analysed.

Such methods fail to take into account the shapes of the waveforms and predisposes the analysis to both type 1 and type 2 errors. Instead, a topological method for detecting statistically significant field changes in n-dimensional continua called Statistical Parametric Mapping (SPM) [30] was employed to overcome these shortcomings. SPM allows for the time-normalised analysis of a waveform in its entirety, such as flexion joint angles, forming a statistical parametric map. Significance is reached only when the value of the test statistic exceeds the test statistic threshold. SPM applicability to that analysis of joint kinematics [31] and clinical gait [32] has been established . In this paper, the SPM analysis toolbox, 1-Dimensional Statistical Parametric Mapping (SPM1D) [33], is applied to falls analyses. The usage of SPM1D with FIMP’s ecologically valid falls allows for the detailed time varying analysis of an individual’s or group of individual’s fall reactive kinematics performance.

## Methods

The FIMP is a system comprised of the following components:

- Mobile Platform
- Trip and slip mechanism
- Subject following and support algorithm
- Gait phase detection algorithm
- User interface and system control

### Mobile platform

The FIMP is 175 cm long, 115 cm wide, and 208 cm tall (Fig. 1). These dimensions were arrived at experimentally, to minimise its footprint while avoiding contact with a subject’s lower limbs. Two motorised wheels were mounted along the central axis of the FIMP such that the FIMP can rotate with a zero turning radius. The 2 DC motors from *Motion Tech Motor* are each rated at 250 W, and were controlled via a Sabertooth 2 $\times$ 32 motor driver. The motor driver was configured in mixed mode, accepting 2 inputs: an input signal that controls the forward speed, and another that controls the turning speed and radius by driving the wheels at different speeds and directions. Five caster wheels were placed around the platform to ensure stability during motion. A 24 V, 35AH Lipo battery can power the entire system for approximately 4 h of continuous movement, however the battery is always charged after a single day trial.

### Trip mechanism

**Fig. 2:**
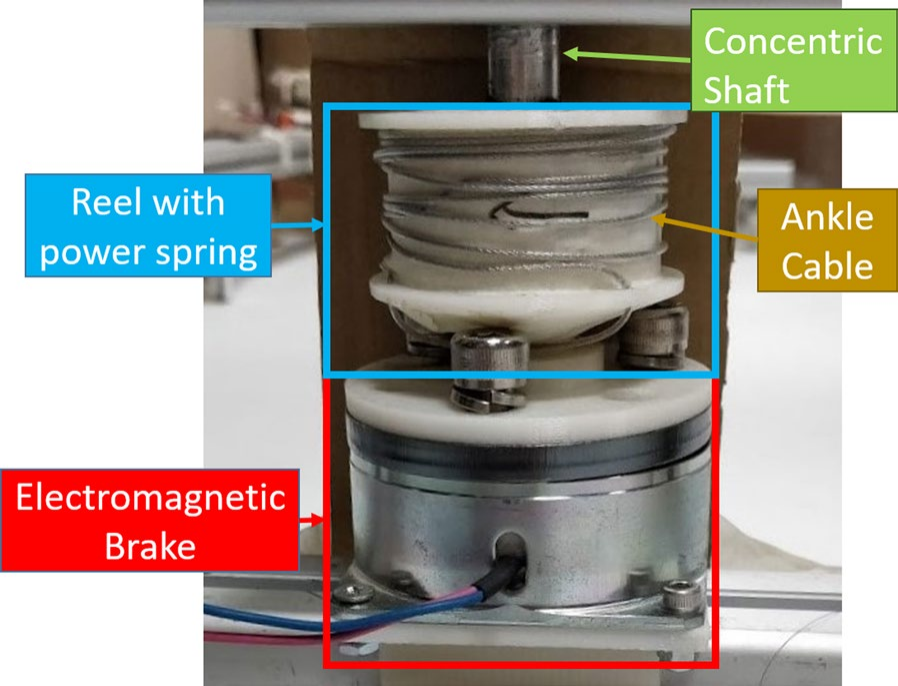
The trip brake mounted on the back of the FIMP. When the brake (red box) is activated, it stops the reel (blue box) from rotating and it prevents the ankle from advancing via the ankle cable tied around the reel. The wire rope is kept taut at all times with a power spring within the reel. A shaft runs through the entire assembly to keeps the reel, power spring and electromagnetic brake concentric with each other

Trip is induced by a posteriorly located electromagnetic brake (Fig. 2, *SINFONIA* ERS-260L, 8Nm maximum holding torque). The brake is attached to the ankle of the subject (green dashed circle in Fig. 1) via wire ropes, henceforth referred to an ankle cable. When a trip is required, the brake activates and generates up to 347.8 N of braking force (Table 1). Otherwise, the brake remains powered off and the power spring connected in series between the brake and ankle pulls with a passive static force of 3.0 N and a dynamic force of 5.9 N. Since the trip motor is posterior to the left leg, trip perturbation is always induced on the left leg.

The power spring ensures that the ankle cable is always taut. A taut ankle cable minimizes any delay between the brake activation time to the ankle feeling the pulling force. This delay controls the precision of the trip timing, which is important for generating different recovery strategies (elevating, lowering, skipping). The power spring also allows the ankle to move relatively free when the brake is deactivated. The impact of power spring pulling forces during normal walking is evaluated as FIMP transparency (Sect. *FIMP Transparency*). The higher the transparency of FIMP, the lesser the impact of the power spring has on an individual’s normal walking gait. Ideally, FIMP should be fully transparent, such that walking with the ankle cable is the same as walking without it.

The severity of the trip can be controlled by 3 factors: (1) perturbation onset, (2) brake activation duration and, (3) walking speed. Perturbation onset dictates the gait phase at which the perturbation is applied. Trips are more severe when it happens in the mid swing phase as the projected BoS on the ground is smaller. This factor is controlled by the gait phase detection algorithm. Increasing the brake activation duration also increases the severity of the trip perturbation and may alter the recovery strategies [34]. A lengthened perturbation duration translates to an increased in undesired momentum that has to be removed during the recovery strategy. The increased undesirable momentum further escalates the difficulty by shortening the remaining duration available for trip recovery (with brake activation duration held constant), preventing the formation of a sufficiently large BoS capable of removing undesirable or unintended momentum. This mimics situations where the swing leg gets tangled on a rope or is in contact with a tall obstacle that elevated toe clearance alone cannot overcome.

The brake activation duration for eliciting different fall recovery responses are listed in Table 2. All of the listed fall types were evaluated in this work with the exception of the elevating strategy as it requires accurate tuning of the perturbation onset and duration. More work needs to be done before the elevating strategy can be studied.

Walking speed is not easily controlled by FIMP, hence subjects were asked to maintain their normal walking speed for all trials.

### Slip mechanism

**Fig. 3:**
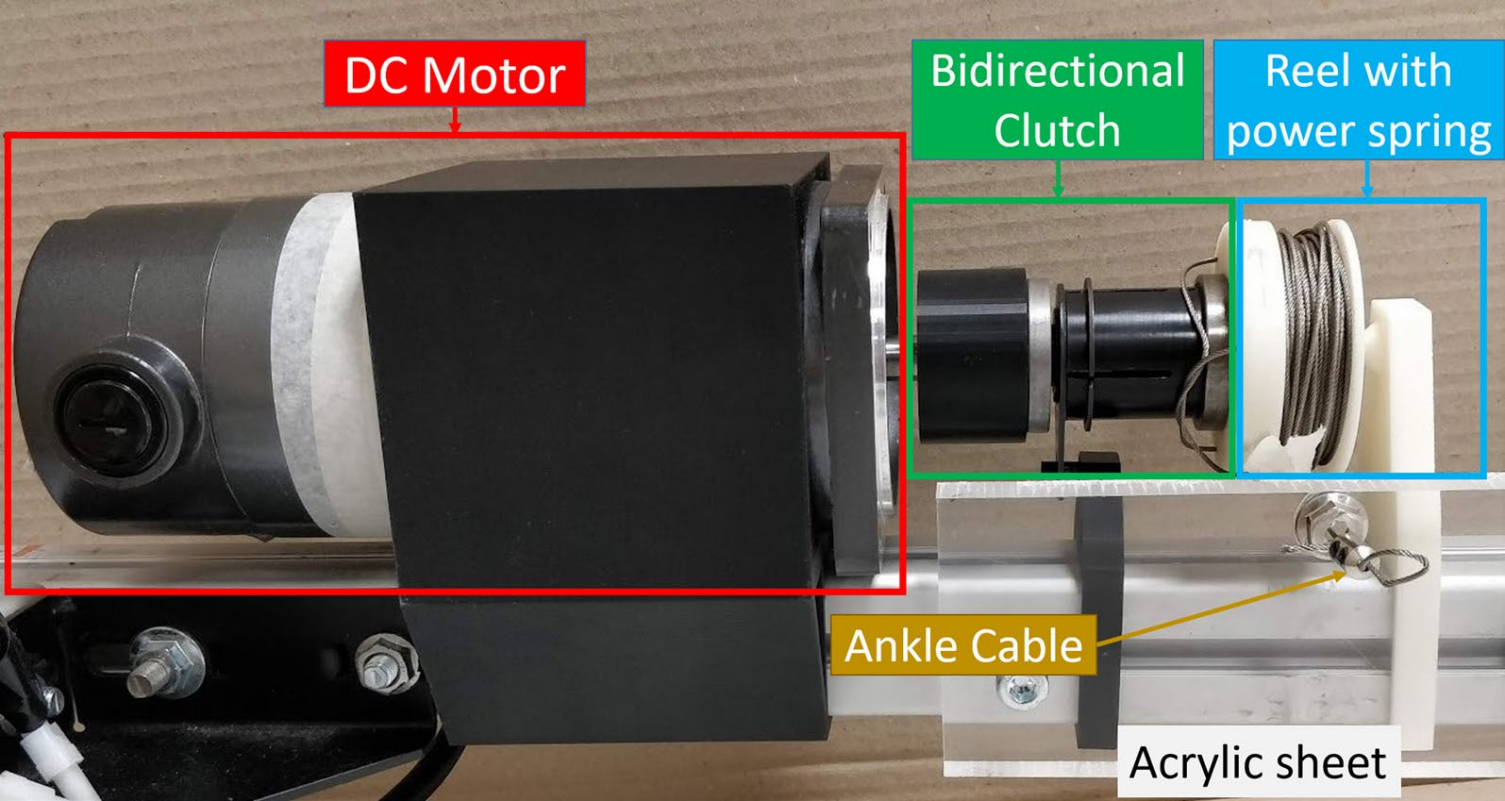
The slip motor (red box) is mounted horizontally, with its output shaft connected to a bi-directional clutch (green box) and finally to a reel of ankle cable. The ankle cable is passed through a thick acrylic sheet to prevent over-reeling. The hole through the acrylic sheet also serves to guide the ankle cable onto the reel and prevents tangling. A resettable fuse in series with the motor acts as a safety measure to prevent the generation of excessive torque/ force. Similar to the trip brake, a power spring is also located within the reel to always keep the ankle cable taut

Slip is induced using a DC motor located anterior to the subject (*XaJong Co. Ltd* M35SWG-2436, 1 Nm continuous torque @ 3600 rpm). The DC motor rotates with 0.12 Nm of torque, translating to approximately 110.3 N of anterior pulling force (Table 1). This approach was chosen as the sudden acceleration that a slipping stance leg experiences can only be recreated with a strong tug (Fig. 3). A wrap spring clutch (*Tiny-Clutch | Helander Product, Inc*) is attached to the output of the DC motor to disengage its inertia from the ankle. A power spring is also placed in series between the wrap spring clutch and the ankle to keep the ankle cable taut. When the motor is disengaged, the power spring pulls with a passive static force of 1.0 N and a dynamic force of 3.5 N. Using both trip and slip power spring system concurrently helps to increase system transparency, as the opposing power springs cancel each other out. Similar to trip, the left leg will always be the perturbed leg as the DC motor is anterior to the left leg.

During slip trials, 2 layers of sliding sheets with coefficient of friction approximately 0.15 between them in both static and dynamic scenarios, were overlain on the pathway to replicate true slip conditions where the slipping foot continues to slide forward after the initial tug from the slip motor. The condition of the shoe outsole should not interfere with the sliding friction as it is the double layer of sliding sheet underneath the shoe that is creating the slippery conditions.

For this investigation of slip, the motor on the FIMP was activated at the beginning of the stance phase. The timing of the perturbation is critical since subjects will only feel a loss of balance if sufficient body weight has been placed on the stance leg prior to the pull from the motor. Pulling marginally earlier at the late swing phase will induce a reaction similar to that in the lowering strategy. On the contrary, pulling later in the stance phase will elicit an unnatural slip perturbation as there is less forward momentum on the foot.

The severity of the slip can be controlled by 3 factors: (1) motor activation duration, (2) pulling force and (3) ground to foot friction. These factors control the forward sliding speed of the stance leg, altering its distance away from the CoM. The further the CoM is behind the stance leg, the greater the difficulty in forming a sufficiently large BoS that can contain the CoM and maintain stability. Since the pulling force of the motor and the ground to foot friction are constants in this work, the fall severity is solely determined by the motor activation duration. The minimum activation duration required to elicit a constant slip is shown in Table 2.

**Table 1:** Ankle cable tension force of different fall mechanisms

	Trip mechanism	Slip mechanism
Static force (N)	3.0	1.0
Dynamic force (N)	5.9	3.5
Max force(N)	347.8	110.3

**Table 2:** Types of fall induced by FIMP

Fall recovery response type	Gait Phase	Fall’s induce mechanism	Activation Timing (ms)
Elevating strategy	Early-mid swing	Brake	<250
Skipping strategy	Early-mid swing	Brake	250
Lowering strategy	Mid-late swing	Brake	400
Slip	Early stance	Motor	250

### Subject follower algorithm

The subject follower algorithm was developed since manual control of a platform prevents precise synchronisation of platform movements with the braking or pulling action of the trip or slip mechanisms. This introduces unwanted variability in the severity of the falls induced. With the algorithm controlling the motion of a motorised platform, the subject is able to walk freely about level ground with changing heading angle, velocity, and gait pattern. The perceivable inertia of a mandatory safety harness can also be greatly reduced if anchored to the moving platform such that it follows the subject without friction.

**Fig. 4:**
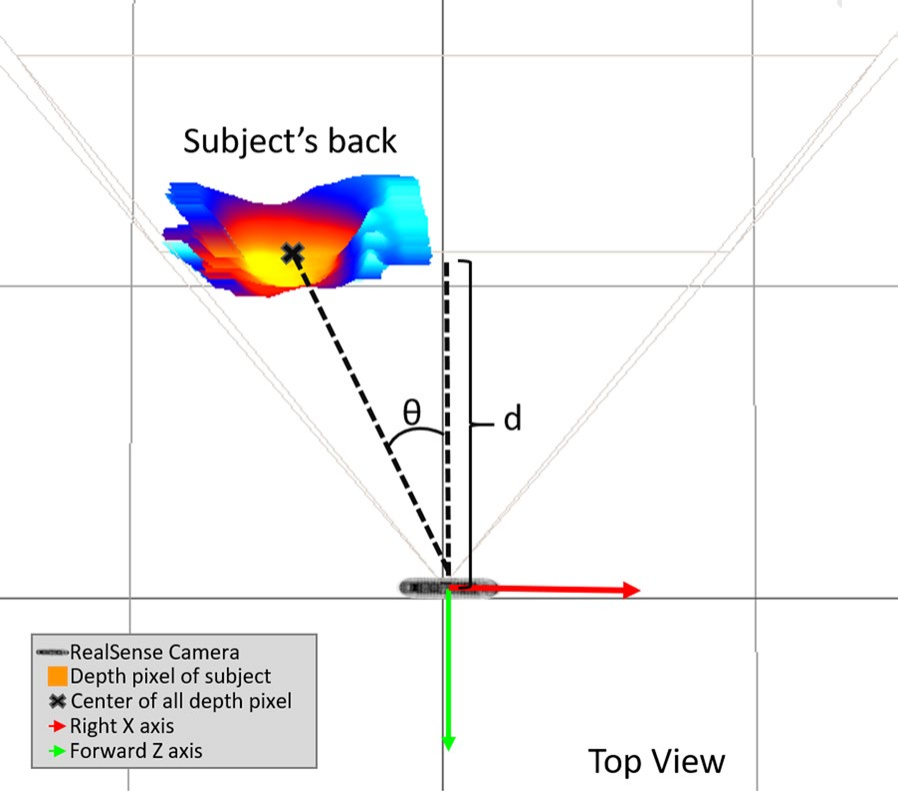
A top view snapshot of a subject’s back view standing in front of a RealSense camera. The position of the average depth pixel (black cross) is used to calculate the distance (*d*) along the forward axis and orientation angle ($\theta$) for the subject follower algorithm

The algorithm is fed with data from an Intel RealSense Depth Camera D435 mounted at the rear of the platform (Fig. 1) and pointed directly forward towards the centre of the FIMP at the subject’s back (Fig. 4). The estimated distance (*d*) of the subject along the Z-axis (forward axis) from the camera is obtained by averaging the depth pixel inputs from the FIMP to be estimated in a contactless manner, eliminating interference with the subject’s normal walking gait. A Proportional Derivative (PD) controller uses this information to control the motorised wheels such that the camera is always positioned 50 cm behind the subject. Additionally, the angle ($\theta$) between 2 vectors (from the average pixel centre of the subject to the camera, and FIMP’s forward axis, Fig. 4) is used to re-centre the subject within the FIMP when the subject turns, via a second PD controller that controls the differential speed of the two motorised wheels.

A step response is normally used to quantify the performance of systems similar to the subject follower. However, the large variance in gait speed and fall recovery movements makes it difficult to choose a reasonable step response for system validation. Instead, FIMP was designed to have a large working area to tolerate large differences in gait parameters and fall recovery movements.

### Gait phase detection algorithm

Rapid and accurate estimation of the gait phase is critical to the induction of the correct fall recovery strategies. The gait phase can be estimated with a single Inertial Measurement Unit (IMU) on the thigh if the body’s sagittal plane always maintains a fixed orientation relative to the global reference frame. The hip angular velocity and hip flexion angle in the sagittal plane were first fed through a single pass second order Butterworth bandpass filter with cutoff frequencies of 0.4 Hz and 3 Hz. Subsequently, the angular velocity and flexion angle is fitted to a unit sine wave (unit magnitude with varying frequency and phase) using Levenberg Marquardt algorithm. The gait phase is calculated from the result as the inverse tangent of the fitted flexion angle over the angular velocity.

If the body’s sagittal plane’s orientation is not fixed, that is, the subject changes his/her heading angle, a single thigh IMU is insufficient to determine the hip’s angular velocity and flexion angle. In this case, the changes in heading angle need to be tracked so that the hip angular velocities and hip flexion angles can be computed in a consistent manner. This was accomplished with an additional IMU on the subject’s torso.

**Fig. 5:**
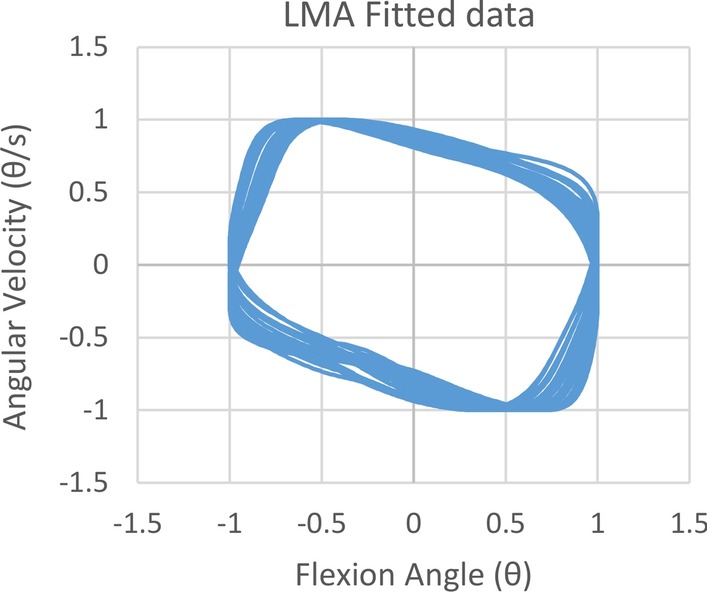
The progression of hip flexion angle versus angular velocity phase portrait after it undergoes bandpass filtration and unit sine wave filtering. The more circular the phase portrait, the better the linearity of the estimated phase angle

**Fig. 6:**
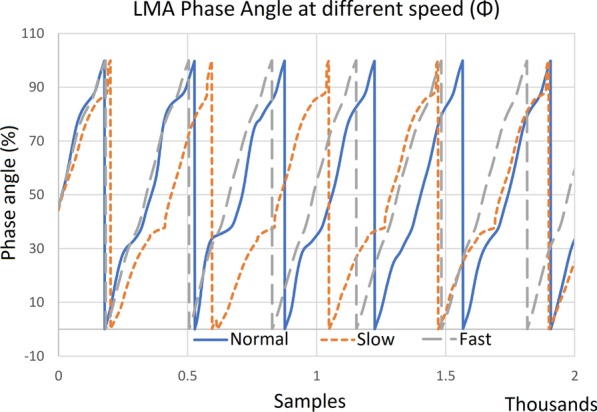
Phase angle for different walking speed (Normal (blue solid line), Slow (orange dotted line) and Fast (grey dash dotted line)). Each walking speed has a different period as seen by the repeating gait cycle

A phase portrait can be used to analyse the performance of the gait phase detection algorithm. An ideal phase portrait should be circular to ensure a linear distribution of the estimated gait phase over the fitted flexion angle and velocity. The phase portrait generated by our algorithm in Fig. 5 does not resemble a circle, but the estimated phase output (Fig. 6) shows a highly linear periodic pattern even for different walking speed. Although complete linearity is not achieved, the periodicity of the gait phase is sufficient for the purpose of perturbation trigger control in this study.

### User interface and system control

**Fig. 7:**
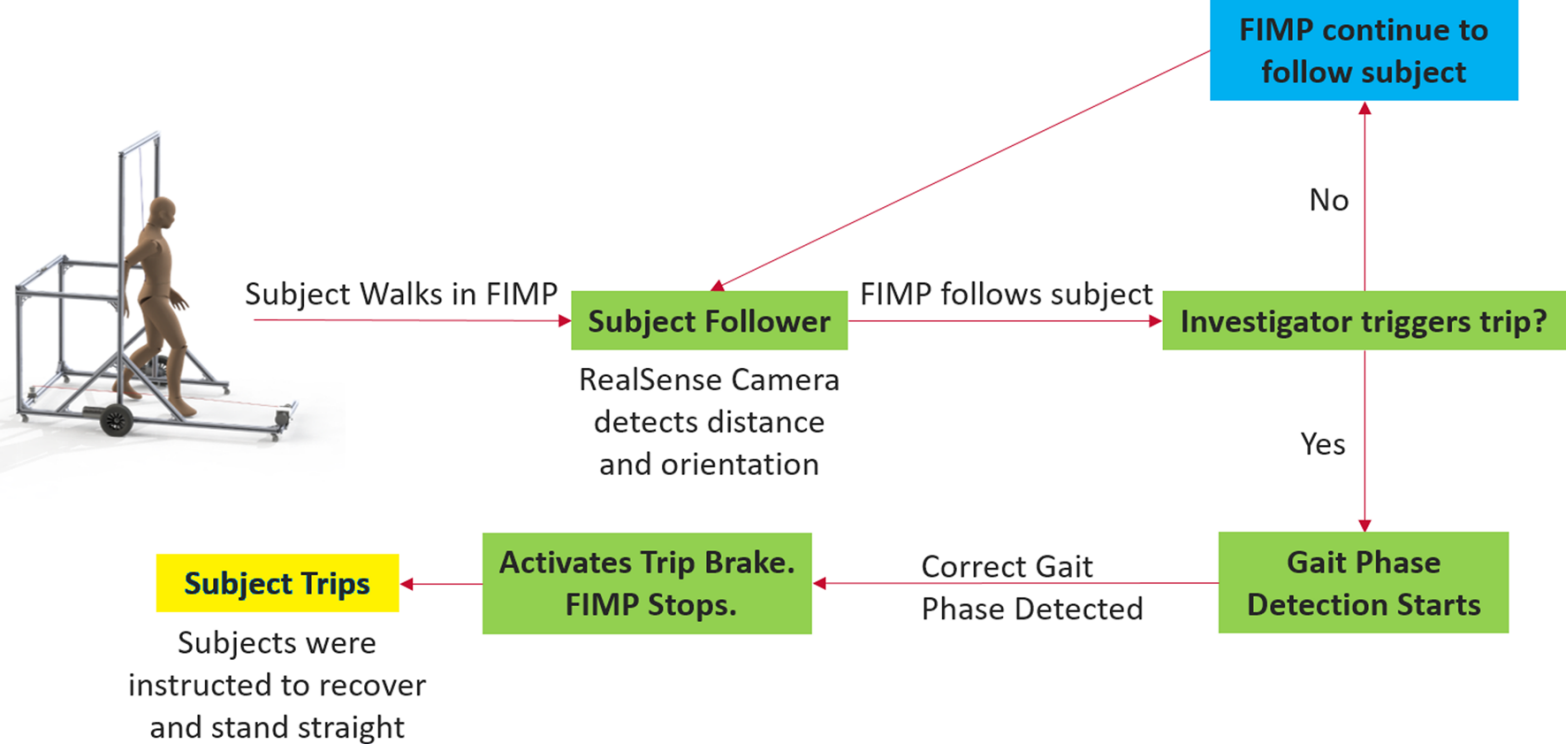
Control flow for the FIMP. Subject starts walking in the FIMP. RealSense camera detects the user’s position and drives the wheels to follow the subject. As the FIMP follows the subject, it polls a “fall button” used by the investigator to signal his/her intention to trip the subject. When the button is depressed, the gait phase detection algorithm is run to detect the appropriate time to trigger the fall inducing mechanism for the pre-selected fall type. When the subject enters the desired gait phase, the fall inducing mechanism is activated as the FIMP is simultaneously brought to a stop. Subjects are instructed to recover and stand straight after the trip

The control flow of the FIMP is shown in Fig. 7. The subject follower algorithm allows the platform to follow the subject around. The platform is equipped with a “fall button” that the investigator can choose to trigger at random. When triggered, the on-board single-board computer (Raspberry Pi 3B+) commences gait phase detection for the subject, the output of which is further discretised into one of twenty equally spaced segments from $-\pi$ to $\pi$. Of these twenty segments, one segment is assigned as the trigger for each type of induced fall. When the detected gait phase enters the segment of the desired fall type, the appropriate fall inducing mechanism is activated and the FIMP comes to a stop. Subjects were instructed to recover from the perturbation as quickly as they could manage and stand straight after the fall. An emergency stop button placed between the Lithium polymer battery power source and the wheels and fall inducing mechanism allows the platform to be shut off quickly in case of emergencies.

### Experimental protocols

All human trials were approved by the Institutional Review Board of Nanyang Technological University (IRB-2018-08-006).

**Fig. 8:**
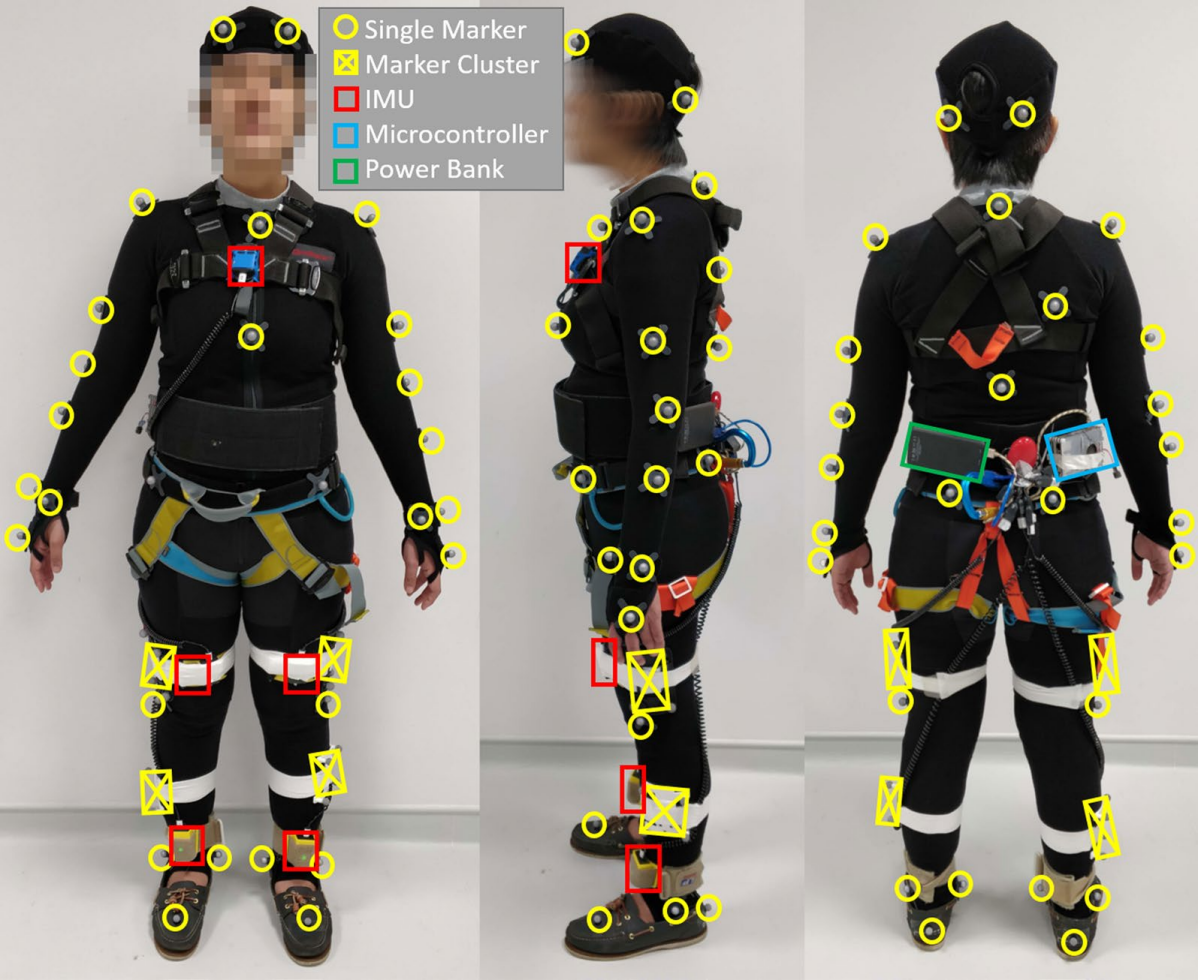
All subjects first puts on a base hook and loop suit, followed by a torso and seat safety harness, and finally the hook and loop belt with the IMU system. The safety harness attached to the top of FIMP will prevent any bodily injuries should the subject fail to perform adequate recovery motions. A total of 5 IMUs and 53 reflective markers were secured to the subject via the base hook and loop suit

5 IMUs were placed anteriorly on the subject’s torso, left and right (L/R) thighs and L/R shanks. The torso and left thigh’s IMUs were used for gait phase detection and the remaining IMUs were for data collection. The IMUs were obtained from *Yost Labs*, and are connected to a single-board computer (Raspberry Pi 3b+) using a USB hub. A mean sampling rate of 333 Hz was obtained with the use of Yost Labs’ proprietary Q-Comp filter. The entire IMU setup was attached to a wide hook and loop belt worn around the subject’s waist (Fig. 8). This waistbelt was secured firmly to prevent downwards slippage, but remained sufficiently loose for comfortable hip and lumbar flexion.

A 16 *Qualysis Miqus M3* motion-capture system with 2 video cameras were used to track and capture subject’s motion. The cameras were configured to sample at 200Hz. 53 reflective markers were placed at the following locations: L/R Forehead, L/R Back head, clavicle, sternum, C7, right back, T10, L/R shoulder, L/R upper arm, L/R elbow, L/R forearm, L/R dorsal tubercle of radius, L/R styloid process of ulna, L/R middle finger’s metacarpophalangeal joint, L/R anterior superior iliac spine, L/R posterior superior iliac spine, L/R thigh (4 markers), L/R lateral knee epicondyle, L/R tibia (4 markers), L/R ankle medial malleolus, L/R ankle lateral malleolus, L/R heel and finally L/R middle toe metacarpophalangeal joint (Fig. 8).

A total of 7 subjects (2 females and 5 males) were recruited for this experiment. Their average age was $25\pm 0.94$ years, had an average height of $168\pm 2.36$ cm, an average weight of $58\pm 6.24$ kg, and an average Body Mass Index (BMI) of $20\pm 1.69$. All subjects had no history of locomotor impairment, neurological impairment or cardiovascular disease.

Each subject was asked to perform 3 types of walking trials at their preferred walking gait velocity along a straight path:

(1) NormalWalking (NW): : 5 trials of walking without ankle cable and without harness.
(2) StrapWalking (SW): : 5 trials of walking with attached ankle cable and with safety harness, but no fall.
(3): Walking with attached ankle cable and harness with randomly induced falls:
  (3.1) MidSwing trip (MS): : 3 trips initiated at mid swing gait phase.
  (3.2) TerminalSwing trip (TS): : 3 trips initiated at terminal swing gait phase.
  (3.3) Slip (SL): : 3 slips initiated at early-stance gait phase.

Due to the space constraints of the laboratory and the limited calibrated motion capture volume (9*m* by 5*m* by 2*m*), all falls were induced within a zone of length 3 m in the middle of the designated pathway (i.e. the 6th to 9th step of a 15 steps pathway). Only the left leg will be perturbed. Randomness was introduced by perturbing the subject only during some of the trials. In these trials, an investigator follows closely behind the FIMP to intervene during emergencies by depressing the emergency kill switch. He/She will also decide whether a fall will be attempted in a particular trial via the “fall button”.

It is known that prior knowledge and experience of slip perturbations will alter the recovery responses of subjects [9, 35]. For consistency, subjects were exposed to the slip perturbation once prior to the commencement of the randomized trials. Analyses of the slip perturbations excludes this first conditioning trial.

For slips (SL), the aforementioned low-friction sliding sheets were placed along the pathway. Trips (MS and TS) and normal walking trials (NW and SW) were conducted on the naked laboratory floor.

All motion captured data were filtered with a dual pass $1^{st}$ order Butterworth low pass filter at 6*Hz* before analysis. For each analysis, the time series data from the sensors were segmented into distinct strides bookended by heel strike events, and normalised feature scaled in time. Each stride was then split into its stance and swing phases, divided by the toe-off event, and scaled to occupy 60% and 40% of the gait cycle, respectively [36]. Data from the left and right legs were compared separately as the ankle strap was only attached to the left ankle during SW trials.

## Results

In all statistical analysis, the results will always be presented in a 4 row by 3 column diagram (Figs. 10, 11, 13, 14, 16 and 17). One-way repeated measure Analysis of Variance (ANOVA) with SPM1D were employed to identify the time instances at which the perturbed gait cycles (either MS, TS or SL trials) deviated from NW and SW. An alpha threshold of 0.05 was chosen and multiplicity correction was not used as outcomes were assessed separately for each of the 3 joints and also for each of the 3 trial types (MS, TS, SL). Each column in the statistical analysis represents the statistical analysis for the hip, knee and ankle joints. The top row shows the mean and the standard deviation clouds for all the NW, SW and either the MS or TS or SL fall trials. Any significant kinematic deviation between the 3 trials will be shown in the second row as grey shaded area above the dotted horizontal line. This horizontal line represents a threshold at which one would expect 5% of statistical maps under the null hypothesis with similar signal smoothness to contain a region of statistical significance [30]. The third row shows significant deviations in the form of colour maps for easy visualization. The colours were separated into intervals of 1 Mean Square of Error (MSE) as the F-value for ANOVA was calculated as the mean of group effects over MSE (Mean Relative Effect). The fourth and last row shows significance for individual subjects (labelled S1 through S7) when compared to their individual NW and SW trials. A deeper and darker red colour indicates a greater statistical significance for that period of gait phase in the third and fourth row.

### FIMP transparency

Due to space, the FIMP transparency analyses can be found in the Additional file 1. From these analyses, it is observed that significant differences were found for the left ankle’s plantar/dorsi flexion kinematics, although the magnitude of difference were minimal and not consistent across subjects. There exists no other significant differences for both the left and right legs joints in the sagittal plane. This implies that the ankle strap has minimal effect on the individual’s normal walking gait.

### FIMP terminal swing tripping effectiveness

This section examines the effect of terminal swing trips on the ipsilateral leg (perturbed left leg) and on the contralateral leg (right leg). The brake’s activation timing was calibrated to arrest the leg’s kinematics for 400*ms* after the detection of the mid swing phase. Results are shown in Figs. 10 and 11.

For each leg, only the gait cycles that contain the instances of perturbations were averaged and analysed.

**Fig. 9:**
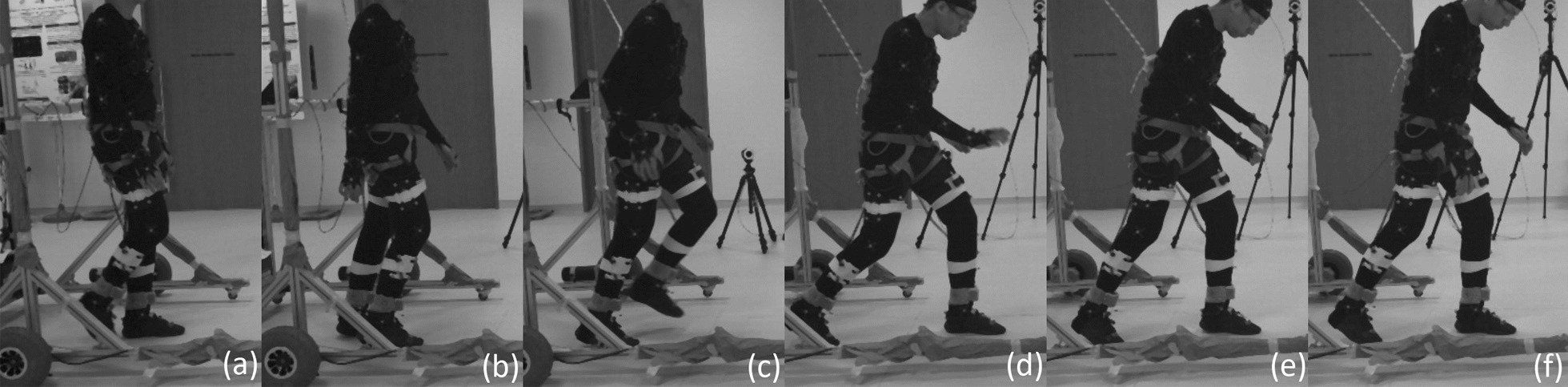
Terminal swing fall sequence (running from left to right) induced by the brake attached to the subject’s left ankle via the ankle cable wire rope. Series of events: **a** Subject encounters perturbation; **b** subject lowers left leg (closer to reader); **c** contralateral right leg lifts off; **d** right leg widens to form large BoS; **e** subject stops descending; **f** subject stops descending and is recovering to a standing posture

#### Left leg (terminal swing trip)

**Fig. 10:**
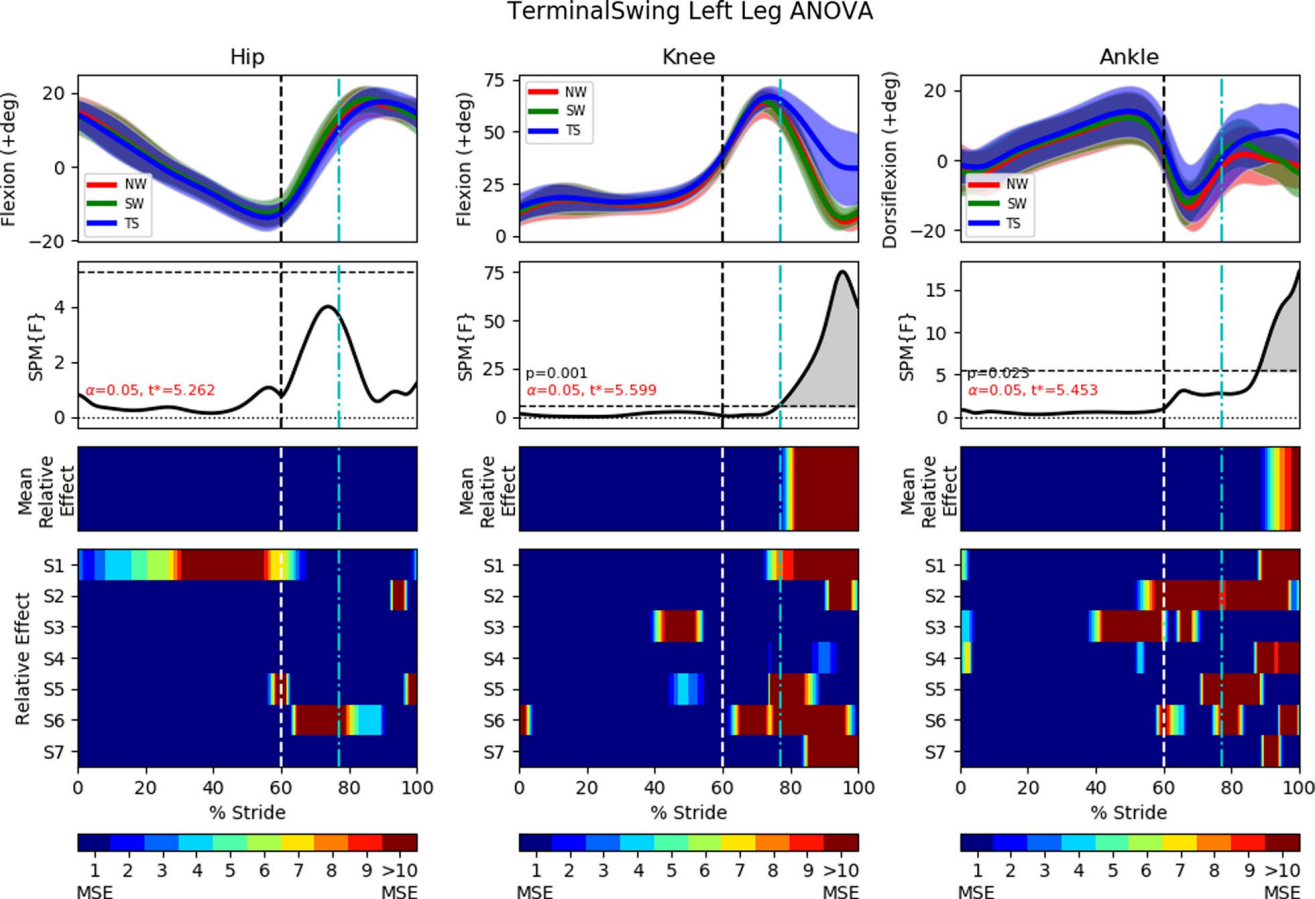
One-way repeated measure ANOVA comparison of NormalWalking (NW) vs StrapWalking (SW) vs TerminalSwing (TS) trials for the left leg with SPM1D. The ankle strap, connected to the electromagnetic brake via the ankle cable wire rope, is worn on the left leg. The top row of graphs shows the mean and the standard deviation clouds for the hip, knee and ankle flexion angles for all subjects; mean (± st.dev). The second row of graphs plots the results of the ANOVA test for the comparison in the top row. Any grey shaded area above the dotted horizontal line indicates significant differences. Any grey shaded area above the dotted horizontal line indicates significant differences. The third row contains colour maps highlighting significance of the ANOVA results in second row, while the last row of graphs shows significance for individual subjects (labelled S1 through S7). Stance phase is located to the left of the black vertical dotted line and swing phase to the right. Perturbation occurs at the location of the cyan vertical dot-dashed line. Statistically significant differences were observed in the knee and ankle joints after the perturbation

The perturbed leg lowers itself during the terminal swing phase. This ipsilateral leg lowering is shown as events (a) to (c) in Fig. 9, and also in the first recording in Additional file 2. Perturbation occurs at approximately $77\%$ of the gait cycle, which translate to a reduction in knee extension and greater dorsiflexion during the mid to late swing phase (Fig. 10). The ankle experienced significantly higher dorsiflexion than in normal walking, starting approximately at $86\%$ of the gait cycle. There is no significant difference observed in the perturbed hip joint between NW, SW and TS.

#### Right leg (terminal swing trip)

The main purpose of the unperturbed leg during a forward trip is for recovery, to extend the BoS and eliminate majority of the angular momentum [37]. In this work, the right leg is always the unperturbed leg.

**Fig. 11:**
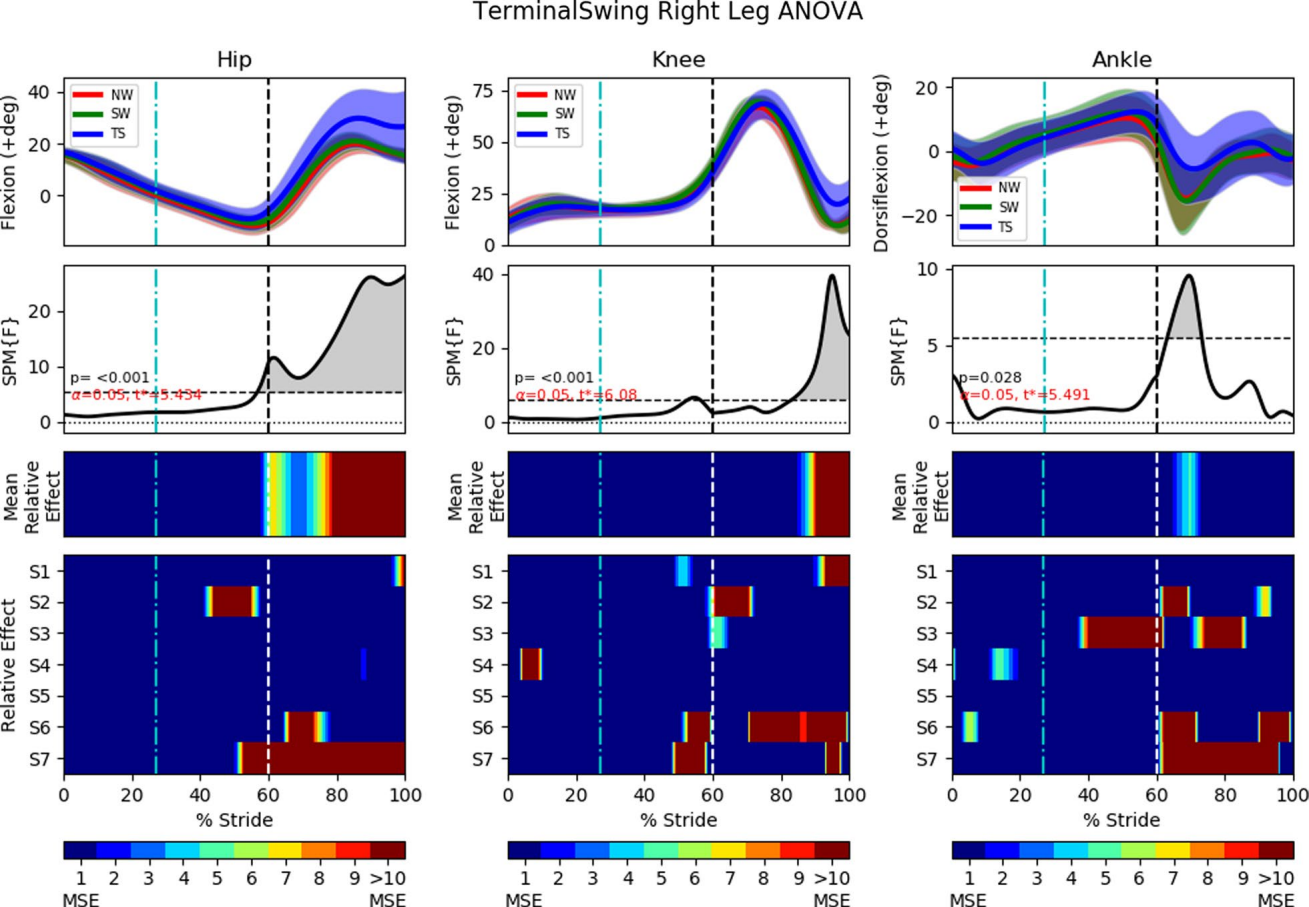
One-way repeated measure ANOVA comparison of NormalWalking (NW) vs StrapWalking (SW) vs TerminalSwing (TS) trials for the right leg with SPM1D. No ankle straps were worn on the right leg. The top row of graphs shows the mean and the standard deviation clouds for the hip, knee and ankle flexion angles for all subjects; mean (± st.dev). The second row of graphs plots the results of the ANOVA test for the comparison in the top row. Any grey shaded area above the dotted horizontal line indicates significant differences. The third row contains colour maps highlighting significance of the ANOVA results in second row, while the last row of graphs shows significance for individual subjects (labelled S1 through S7). Stance phase is located to the left of the black vertical dotted line and swing phase to the right. Perturbation occurs at the location of the cyan vertical dot-dashed line. The most pronounced difference between NW and SW was found in the hip joint. The knee joint saw greater flexion angles at the terminal swing phase, while the ankle joint saw greater dorsiflexion at the early to mid swing phase

The contralateral right leg switches from stance to swing phase immediately after the perturbed leg has touchdown. This phase transition is seen in events (b) to (c) in Fig. 9, and also in the first recording in Additional file 2. Thereafter, the contralateral leg reaches out to widen the BoS, as seen in events (c) to (d). The quicker temporal transitioning of stance to swing gait phases cannot be observed in Fig. 11 as the stance and swing phase has been time normalized. However, the rapid extension of the contralateral leg is easily observed in the hip kinematics, as significant increase in hip flexion occur as soon as swing phase has started ($60\%$ of gait cycle). The rapid forward roll of the subject’s body also induced higher hip flexion to occur before the transition to swing phase (from approximately $56\%$ to $60\%$ of the gait cycle).

The knee also experienced significant differences in their flexion angle as the hip widens to create a larger BoS. The knee joint has higher flexion angle at the mid-end swing phase (starting from $83\%$ of gait cycle).

Interestingly, the ankle showed significant increase in dorsiflexion only during the early to mid swing phase ($63\%$ to $73\%$ of gait cycle), while the hip and knee manifested differences until the end of swing phase.

### FIMP mid swing tripping effectiveness

This section examines the effect of mid swing trips on the ipsilateral leg (perturbed left leg) and on the contralateral leg (right leg). The brake’s activation timing was calibrated to arrest the leg’s kinematics for the majority of the early-mid swing phase (250*ms*). Results are shown in Figs. 13 and 14.

For each leg, only the gait cycles that contain the instances of perturbations were averaged and analysed. Similarly, as per the previous analyses, ANOVA with SPM1D analysis were applied with an alpha of 0.05.

**Fig. 12:**
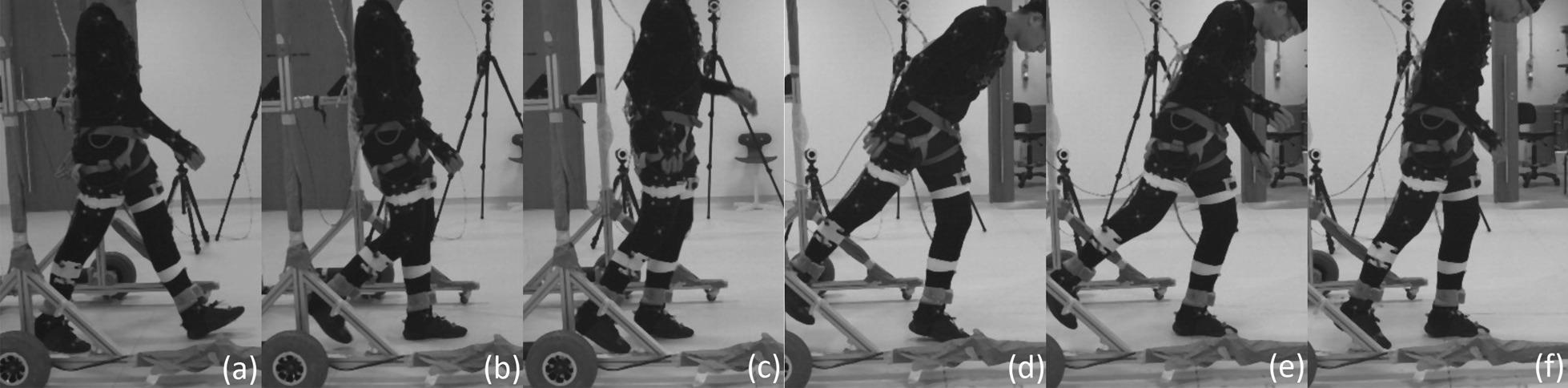
Mid swing fall sequence (running from left to right) induced by the brake attached to the subject’s left ankle (closer to the reader) via the ankle cable wire rope. Series of events: **a** Subject’s left leg is starting early swing phase; **b** left leg encounters perturbation and the body rolls forward rapidly; **c** subject tries to lower the left leg, but it barely touches the floor; **d** contralateral right leg skips forward to form large BoS; **e** subject tries to prevent further forward rotation; **f** subject stops descending and is recovering to a standing posture

#### Left leg (mid swing trip)

**Fig. 13:**
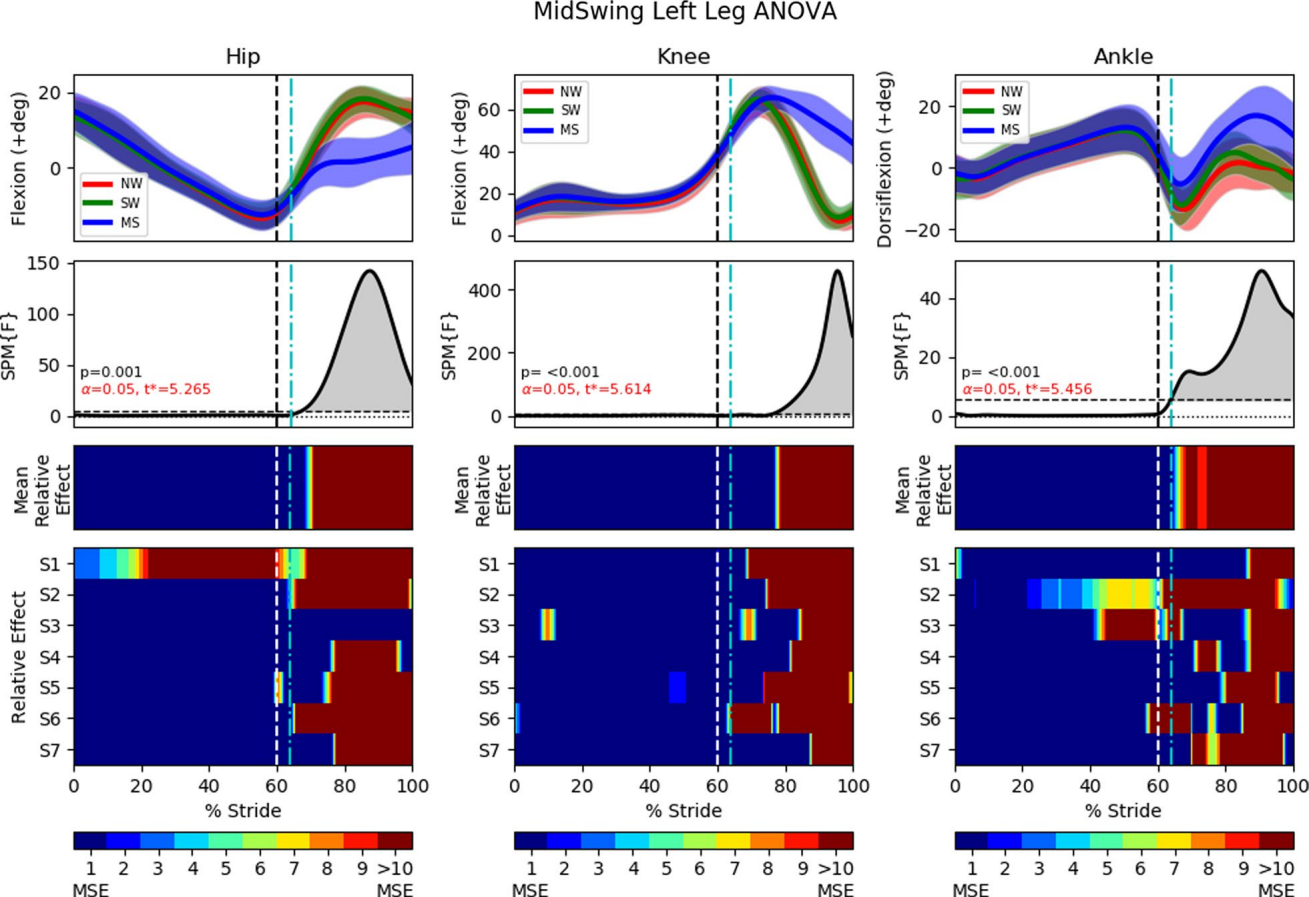
One-way repeated measure ANOVA comparison of NormalWalking (NW) vs StrapWalking (SW) vs MidSwing (MS) trials for the left leg with SPM1D. The ankle strap, connected to the electromagnetic brake via the ankle cable wire rope, is worn on the left leg. The top row of graphs shows the mean and the standard deviation clouds for the hip, knee and ankle flexion angles for all subjects; mean (± st.dev). The second row of graphs plots the results of the ANOVA test for the comparison in the top row. Any grey shaded area above the dotted horizontal line indicates significant differences. The third row contains colour maps highlighting significance of the ANOVA results in second row, while the last row of graphs shows significance for individual subjects (labelled S1 through S7). Stance phase is located to the left of the black vertical dotted line and swing phase to the right. Perturbation occurs at the location of the cyan vertical dot-dashed line. Significant differences were observed in the knee and ankle joints near the terminal swing phase

Leg lowering can be seen in events (a) through (c) in . 12, and also in the second recording of Additional file 2. The perturbation occurred at approximately $64\%$ in the gait cycle, at the start of the early to mid swing phase. This early perturbation prevents the hip joint from fully flexing as normal, with the difference visible in Fig. 13. This difference extends until the end of the swing phase with the hip joint never reaching the same angle of flexion as in the NW and SW trials.

The knee joint saw much higher flexion at the end of the swing phase, most likely due to the reduction in swing duration. Interestingly, the knee joint only differed significantly from the NW and SW trials starting at approximately $77\%$ of the gait cycle even though the perturbation started at $64\%$ of the gait cycle.

Unlike the knee joint, the ankle joint deviated from the NW and SW trials as soon as perturbation is applied. A more elaborate dorsiflexion strategy was observed in contrast to the maintenance of a fixed orientation in the ipsilateral leg of the lowering strategy.

#### Right leg (mid swing trip)

**Fig. 14:**
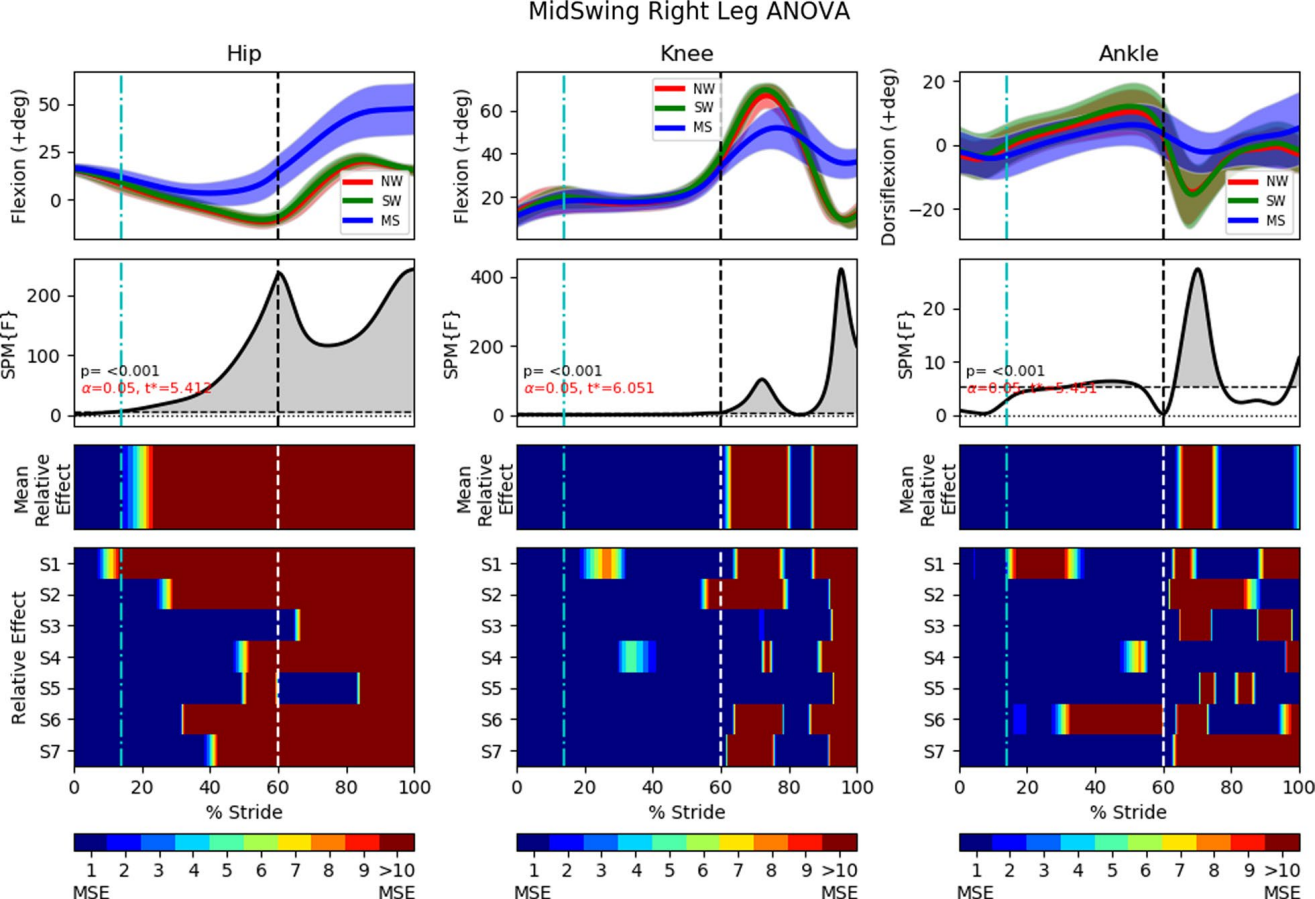
One-way repeated measure ANOVA comparison of NormalWalking (NW) vs StrapWalking (SW) vs MidSwing (MS) trials for the right leg with SPM1D. No ankle straps were worn on the right leg. The top row of graphs shows the mean and the standard deviation clouds for the hip, knee and ankle flexion angles for all subjects; mean (± st.dev). The second row of graphs plots the results of the ANOVA test for the comparison in the top row. Any grey shaded area above the dotted horizontal line indicates significant differences. The third row contains colour maps highlighting significance of the ANOVA results in second row, while the last row of graphs shows significance for individual subjects (labelled S1 through S7). Stance phase is located to the left of the black vertical dotted line and swing phase to the right. Perturbation occurs at the location of the cyan vertical dot-dashed line. Significant differences were observed in the knee and ankle joints near the terminal swing phase

The contralateral leg transitions to the swing phase before the perturbed leg has touchdown. The rapid transition from stance to swing in the skipping strategy is shown in events (c) through (e) in Fig. 12 and the second recording in Additional file 2. In addition, SPM and ANOVA analyses of the MS trial in Fig. 14 show that hip kinematics deviate from NW and SW trials rapidly post-perturbation, whereas significant kinematics deviation only occur during the swing phase for the lowering strategy (Fig. 11).

Knee kinematics deviated from the NW and SW trials as soon as the swing phase starts at $60\%$ of the gait cycle. It has a smoother trajectory with less flexion throughout the entire swing phase, reaching a maximum of $52.0\deg$ versus $66.8\deg$ and $69.5\deg$ for normal walking and strap walking, respectively.

The ankle joint saw elevated plantar flexion at the mid to late stance phase ($29.8\%$ to $53.5\%$) as the propulsive force for push-off was generated. In the subsequent swing phase, all subjects maintained a near constant ankle flexion, similar to the right ankle of the TS trials (Fig. 11).

### FIMP slipping effectiveness

This section examines slip induced recovery kinematics on the ipsilateral leg (perturbed left leg) and on the contralateral leg (right leg). The slip’s motor was activated for 250*ms* at the beginning of the the stance phase. Analyses of the slip perturbations excludes this first conditioning trial. As per the previous analyses, ANOVA with SPM1D analysis were applied with an alpha of 0.05. Results are shown in Figs. 16 and 17.

**Fig. 15:**
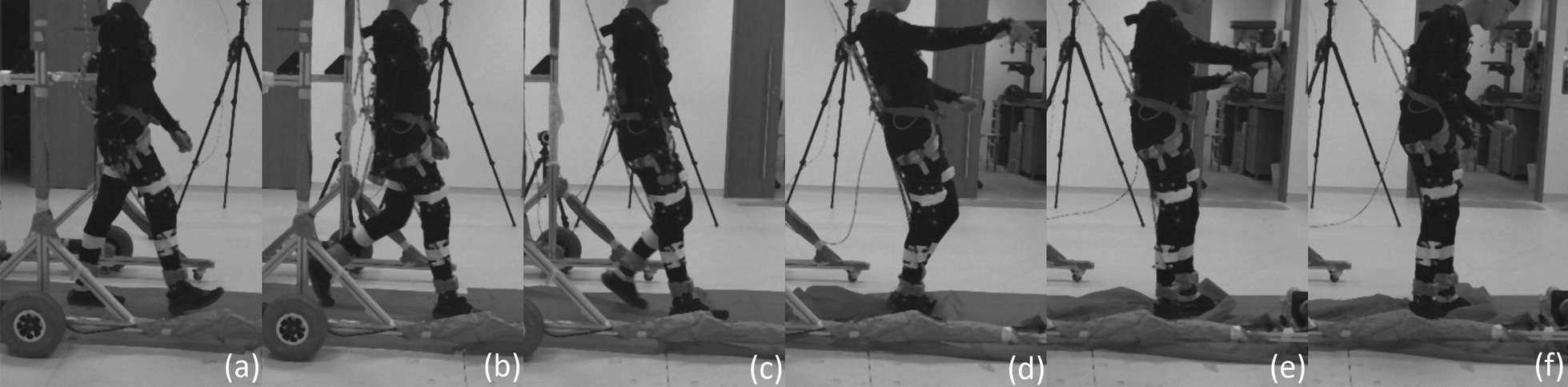
Slip fall sequence (running from left to right) induced by the motor attached to the subject’s ankle via the ankle cable wire rope. A low friction sliding sheet was placed on the ground to reduce the friction between the foot and the ground. Series of events: **a** left foot approaching the end of swing phase; **b** left foot enters the stance phase and perturbation is applied (motor starts pulling); **c** perturbed left foot slides forward and the right legs straightens for touchdown; **d** right foot touches down and slide forward together with the left foot; **e** both legs slide forward together; **f** sliding stops and subject is recovering to a standing posture

#### Left leg (slip)

**Fig. 16:**
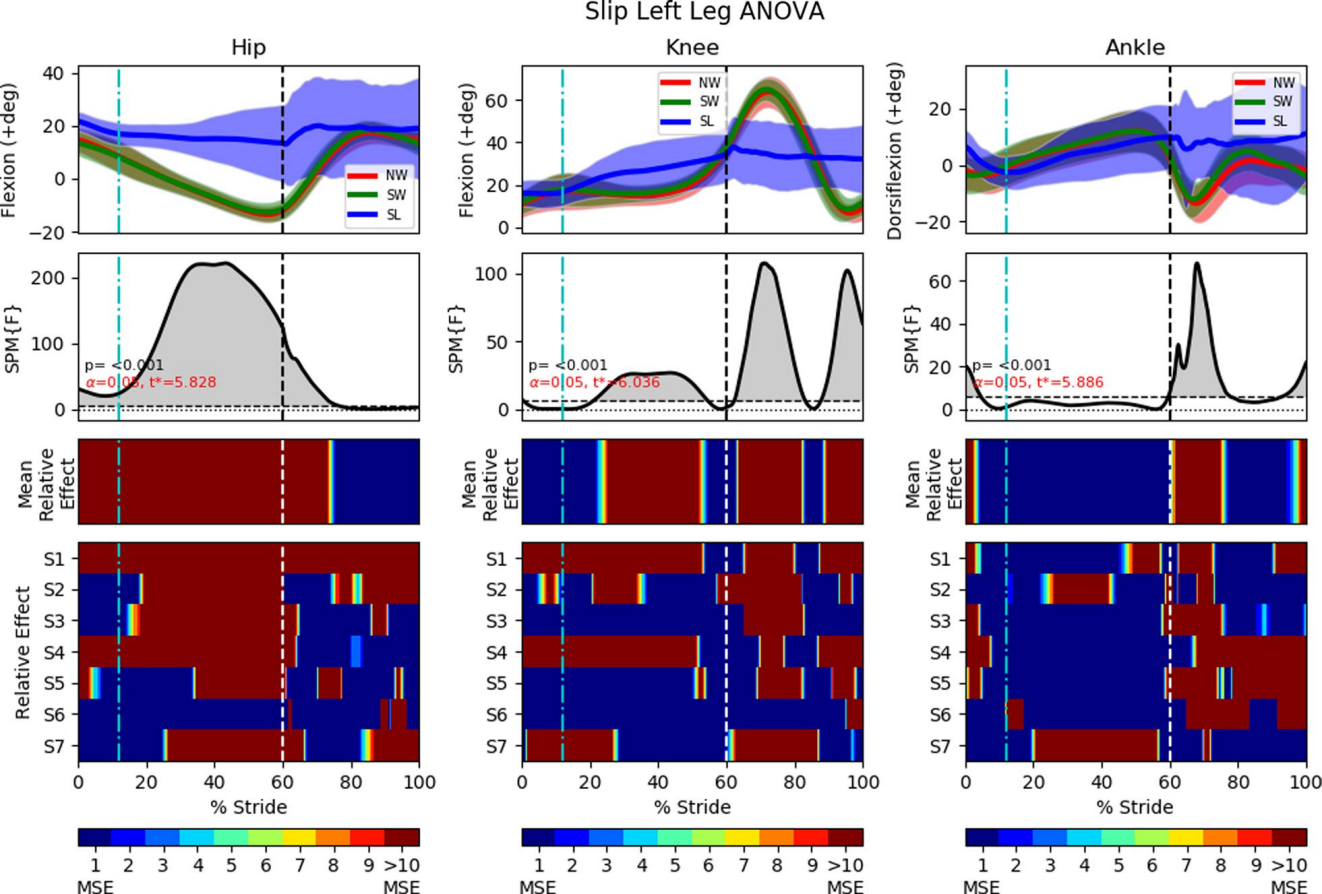
One-way repeated measure ANOVA comparison of NormalWalking (NW) vs StrapWalking (SW) vs Slip (SL) trials for the left leg with SPM1D. The ankle strap, connected to the DC motor via the ankle cable wire rope, is worn on the left leg. The top row of graphs shows the mean and the standard deviation clouds for the hip, knee and ankle flexion angles for all subjects; mean (± st.dev). The second row of graphs plots the results of the ANOVA test for the comparison in the top row. Any grey shaded area above the dotted horizontal line indicates significant differences. The third row contains colour maps highlighting significance of the ANOVA results in second row, while the last row of graphs shows significance for individual subjects (labelled S1 through S7). Stance phase is located to the left of the black vertical dotted line and swing phase to the right. Perturbation occurs at the location of the cyan vertical dot-dashed line. All the joint angles were relatively constant as compared to NormalWalking. This indicates that stiffening of joints occur during slips

The perturbed leg slides forward in a flat-footed posture after a slip perturbation [9]. This is shown in events (b) through (f) in Fig. 15 and in the third and fourth recordings in Additional file 2. The slip perturbation starts at approximately $12\%$ of the gait cycle, corresponding to the sharp increase in hip flexion (Fig. 16). The hip, knee and ankle held a steady flexion angle prior to the swing phase after the perturbation.

The adoption of this posture is by itself insufficient to effect complete recovery from a slip, with the body will leaning forward as the slip progresses. At this point, subjects commonly used their upper limbs and body dynamics to generate angular momentum to counter the incipient forward lean (in 17 of 27 slip trials), or take an additional anterior recovery step to eliminate the remaining undesired momentum (in 4 of 27 slip trials). Some subjects found themselves taking a posterior recovery step if their CoM had shifted too far anteriorly (in 6 of 27 slip trials). As the anterior and posterior recovery steps both occurred in the swing phase, adequate analysis cannot be performed without examining these distinct scenarios separately. Further research is required to gather more data so that each scenario can be examined in detail to determine their causes and effects.

A majority of the slip trials (17 out of 27 slip trials) saw successful recovery without the taking of an additional recovery step (posterior or anterior), with most of the undesired momentum being eliminated in the stance phase.

#### Right leg (slip)

The contralateral right leg is observed to perform 2 types of slip recovery responses: (1) lowering of the contralateral leg and sliding forward together with perturbed leg and (2) completion of swing phase by the contralateral leg before sliding forward on both legs. The first response can be seen in events (d) through (f) in Fig. 15 and in the third recording in Additional file 2. In this investigation, it occurred in 10 of 27 SL trials. The second response has the right leg completing the swing phase and placing itself anterior to the left leg. Subsequently, both legs slide forward together until the undesired momentum has been entirely bled off (fourth recording in Additional file 2, occurring in 17 of 27 SL trials). The swing phase as defined in this study includes the kinematics of both recovery responses even though the former utilises a lowered, sliding contralateral leg. This is due to the absence of a heel strike to demarcate the end of the swing phase and start of a stance phase.

Since the knee and ankle joints showed significant kinematic deviations coinciding with the initiation of the slip perturbation, the timing of the perturbation is not a factor in determining the recovery response elicited. The knee adopted a relatively constant flexion angle throughout the entire perturbed swing phase, indicating that subjects were trying to extend their legs towards the floor even if the second recovery response (completing the swing phase before sliding forward) is observed. This resulted in a much shorter step length ($0.26\pm 0.14$ m), as compared to NW ($0.64\pm 0.24$ m, $p<0.0001$) and SW ($0.65\pm 0.19$ m, $p<0.0001$). The ankle by and large conforms to this general trend of motion for the NW and SW trials with the exception of slightly higher dorsiflexion. This is likely due to the swing phase terminating earlier than the NW and SW, indicated by the shorter step length.

**Fig. 17:**
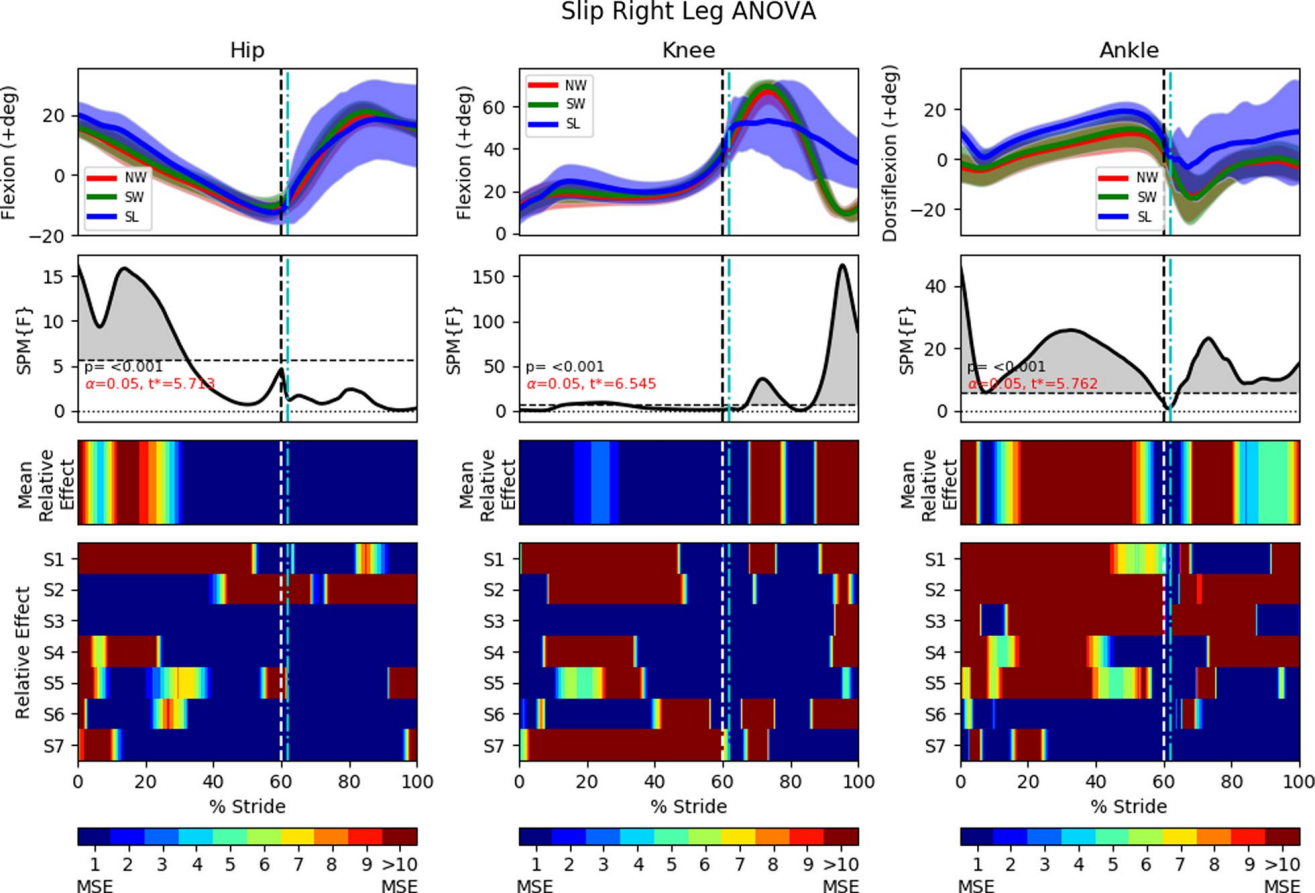
One-way repeated measure ANOVA comparison of NormalWalking (NW) vs StrapWalking (SW) vs Slip (SL) trials for the right leg with SPM1D. No ankle straps were worn on the right leg. The top row of graphs shows the mean and the standard deviation clouds for the hip, knee and ankle flexion angles for all subjects; mean (± st.dev). The second row of graphs plots the results of the ANOVA test for the comparison in the top row. Any grey shaded area above the dotted horizontal line indicates significant differences. The third row contains colour maps highlighting significance of the ANOVA results in second row, while the last row of graphs shows significance for individual subjects (labelled S1 through S7). Stance phase is located to the left of the black vertical dotted line and swing phase to the right. Perturbation occurs at the location of the cyan vertical dot-dashed line. The right knee and ankle maintained a constant joint angle throughout the swing phase, indicating the adoption of the surfing strategy (keeping foot flat and close to ground)

## Discussion

The FIMP system presented here provides a realistic simulation of trips and slips caused by external factors. FIMP differs from other fall inducing systems [12, 29] in that it applies perturbation forces on the ankle to replicate obstacle collision and slippery floor sliding while performing overground walking. The perturbation forces are transmitted via cables attached to the ankle from a posteriorly mounted electromagnetic brake for trips and an anteriorly mounted DC motor for slip. Overground walking allows true recovery responses which is not possible with treadmill walking as it is highly improbably that the treadmill can perfectly track the recovery limb to avoid artificially widening or narrowing of subject’s BoS. The minimal difference between walking with and without FIMP (Additional file 1) strongly increase the credibility of conducting fall studies with FIMP.

### Individual gait variance

Figures 10, 11, 13, 14, 16, 17 show the significance of lower limbs recovery kinematics for all subjects combined (top 3 rows) and also the individual subjects (fourth row). It is observed that certain regions which show significant differences for the combined trial analysis did not appear when comparing individually.

An example would be the right hip kinematics for the terminal swing trips (Fig. 11). Significant differences were seen on the combined trials for the entire swing phase, but only subject 7 showed significant difference when compared individually. Subjects 1 through 6 could be observed to show differences between their swing phases in TS trials and NW and TS trials (pages 26 to 31 in Additional file 4). Yet, no significance was found because of high variance that exists between the limited number of falls recorded (minimum of 3 falls).

Another example is shown in the left hip kinematics for mid swing trips (Fig. 13). All subjects except subject 3 were showing significant different in their left hip during the middle of the swing phase. However, focusing on subject’s 3 kinematics (p. 36 in Additional file 4), it is seen that similar kinematic deviations of the left hip is occurring, but significance was not detected because of the large variation between trials.

The proposed solution will be to increase the number of fall recordings, However, a balance must be found between the need to maintain the element of surprise (falls are induced randomly between normal walking trials) and the physical/mental exertion of the subjects. In this single day study, a minimum of 9 falls (TS, MS, SL trials) were induced between a minimum of 27 normal walking trials, and subjects feedback that they were feeling tired. Alternatively, each fall types can be conducted on a separate day for more fall trials.

### Terminal swing trip

Perturbations during the terminal swing gait phase is reported to elicit the lowering recovery strategy [6], which corresponds to the active lowering of the perturbed leg followed by the contralateral leg to overcome the obstacle (Fig. 9 and Additional file 2). Contrary to the reported leg lowering, our data suggest that the perturbed leg did not actively lower itself (no significance in hip kinematics). Instead, post-perturbation stiffened knee joint (reflex action [10]) and increased forward body roll (due to gain in unwanted angular momentum) created the illusion of active leg lowering.

The kinematics of the contralateral leg suggests that it is performing an elaborate swing phase to overcome the *obstacle* simulated by FIMP. This is observable from the significant increase in hip flexion that generally widens the BoS. An increase in knee flexion was also observed at the mid-end swing phase ($83\%$ of gait cycle). This may have occurred due to 2 factors: (1) the lowering of the body height during trip and the gain of unwanted momentum reduces the duration for knee extension to occur and, (2) rapid hip flexion imparting additional rotational speed to the shank (similar to a double pendulum) creating larger knee flexion. Unlike the contralateral hip and knee, the contralateral ankle has increased flexion (dorsiflexion) only during the early swing phase ($63\%$ to $73\%$ of gait cycle). This be may the effect of subjects attempting to increase toe-clearance during the early swing and relaxing the ankle after overcoming the *obstacle*.

Overall, FIMP’s terminal swing trip mechanism elicited a recovery strategy that agrees with the widely reported lowering strategy. The range of fall recovery kinematics shown in Figs. 10 and 11 indicate that subjects’ recovery kinematics are repeatable to a certain extent across multiple trials. This suggests that FIMP is capable of inducing ecologically valid terminal swing trips. The post-hoc pairwise comparison between NW, SW and TS trials are shown in Additional file 3.

### Mid swing trip

The elevating recovery strategy is typically reported for mid swing gait phase perturbations [6]. However, this is only valid for falls that are easy to overcome by raising the perturbed leg over the obstacle. When this is not possible, the skipping strategy [8] is elicited instead. The skipping strategy performs like a rapid execution of the lowering strategy with larger undesired momentum. The large undesired momentum is gained from arresting the leg when the projected BoS is small (i.e. during swing phase when legs are in-line with each other in sagittal plane). Subjects must rapidly widen their legs to capture the undesired momentum.

As the body rolls forward about the contralateral ankle after the perturbation, the ipsilateral leg tries to widen the BoS but is kinematically constrained by the trip mechanism. Therefore, as Fig. 13 shows, the hip and knee flexes slightly, post perturbation, before the knees extend for touchdown. Since the hip joint angle is calculated as the angle between the torso and thigh, the forward body roll gradually increases the hip flexion angle even as the ipsilateral leg straightens for touchdown. This flexing of hip and knee before straightening for touchdown is similar to the delayed lowering strategy reported by other fall studies [34, 38]. The contralateral ankle in terminal swing trips and the ipsilateral ankle in mid swing trips share the pattern of an increased dorsiflexion.

Unlike in TS trials, our data show that significant differences occur in the stance phase of the contralateral leg soon after perturbation. The hip experienced a rapid increase in flexion after the ipsilateral leg perturbation to bring the leg forward. Additionally, the ankle joint saw elevated plantar flexion at the mid to late stance phase ($29.8\%$ to $53.5\%$) as the propulsive force for push-off was generated. The contralateral right knee adopted an extended configuration in the swing phase which imparted benefits such as a greater reserve of joint torque and angle to absorb the greater impact when landing in the skipping strategy.

Generally, the MS trials generated kinematics that matched that reported by other fall studies, including the skipping strategy [8] and the delayed lowering strategy [34, 38]. The post-hoc pairwise comparison between NW, SW and MS trials are shown in Additional file 3.

### Slip

Slip recovery requires subjects to stiffen their hip joint muscles [23, 35] while adopting a flat-footed posture to reduce the push-off forward velocity [9]. This posture creates a torque to oppose the forward linear and rotational momentum resulting from the slip.

However, it was observed that this stiffening and flat-footed response was insufficient for full slip recovery. Our data reveal that the left leg uses 3 different responses to the slip perturbation:

1. Continuous slip before coming to a stop (17 out of 27 trials)
2. Slip with an additional anterior recovery step (4 out of 27 trials)
3. Slip with an additional posterior recovery step (6 out of 27 trials)

The existence of 3 distinct responses complicated the analysis of the swing phase for the perturbed leg. Nevertheless, the stance phase in which the subject slides along the floor on their perturbed foot remains common across all trials. This common posture suggests that a majority of the undesired momentum was eliminated in this phase with the stiffening of the hip, knee, and ankle joints.

Similarly, the right leg was observed to have 2 different slip recovery responses:

1. Lowering of the contralateral leg and sliding forward together with perturbed leg (10 out of 27 trials)
2. Anterior swing of the contralateral leg before foot touchdown and sliding forward with the perturbed leg (17 out of 27 trials)

As the slip perturbation happened during the initial swing phase of the right leg, its hip and ankle kinematics did not deviate much from the general trend of the NW and SW trials. The lowering of the right leg to increase ground-foot friction in a replication of the flat-footed surfing strategy was reflected in an elevated right knee extension in both types of recovery responses. The post-hoc pairwise comparison between NW, SW and SL trials are shown in Additional file 3.

## Limitations

The major limitation of this study is the lack of force and electromyography (EMG) analysis. These additional information would provide critical clues that can determine the causes of some observations highlighted in the Discussion section, such as the increased ipsilateral knee flexion for TS trials. The overground nature of FIMP meant that large and sensitive equipment such as force plate and EMG amplifiers cannot be easily used as they are sensitive to motion artifact. Taking into account the cost and setup time, these sensors are very costly investments that are only justifiable if FIMP does indeed induce kinematically realistic falls. Hence, the decision was made to forego the collection of these data in this pilot study. Given the positive results with respect to the induction of realistic falls from this study, future studies may look to incorporate such measurements by conducting the experiment over a pressure measuring walkway with subjects wearing wireless EMG sensors.

This study also limited in the types of fall induced and the methods of inducing falls. Firstly, this study only examines externally induced falls, when a significant portion of all falls are due to internal factors [5]. Externally induced falls which elicit the elevating strategy are also not studied. Secondly, it was assumed that the minor, though significant, kinematic difference observed in the left ankle during system transparency test can be neglected. Thirdly, the symmetricity of fall recovery motions are assumed as only the left leg is perturbed. Influence of dominant leg on recovery motions were also not considered. Lastly, we assumed that the change in gait pattern after repeated exposure to trips and slips will not be significant. Future studies are planned to verify these assumptions and to examine more fall types with greater number of falls.

## Conclusion

A novel fall inducing movable platform (FIMP) system was developed and shown to to be capable of effectively inducing both slips and trips during overground walking. The system does not limit its subjects to a constant heading angle, walking velocity or gait pattern, a limitation inherent in treadmill-walking. Additionally, the safety harness’ being anchored to the top crossbeam of the FIMP and moving in sync with the subject, enabled by the subject follower algorithm, greatly reduced any inhibitory effects the mandatory safety feature had on their individualised movement pattern.

Trips and slips can be induced via FIMP’s fall mechanisms attached to the ankle. These mechanisms were shown to have minimally effects on subjects’ normal walking gait. FIMP can thus be employed in fall studies without reservations. Terminal swing trips which induce the lowering strategy can be reproduced, with the characteristic leg lowering and rapid rise of the contralateral leg to regain balance readily observed. Skipping strategies were induced via mid swing trips, where the perturbed leg works to return to normal kinematics while the hip joint is arrested. Since the perturbed leg cannot reach the ground sufficiently rapidly, the contralateral leg needs to swing forward to widen the BoS. Slips were induced with FIMP with the help of sliding sheets to reduce ground-foot friction. Slip trials presented much higher kinematics variability as a number of recovery strategies were employed. Common across them was the stiffening of the perturbed leg while the recovery leg attempted to lower itself to impose a flat-foot configuration. Overall, FIMP has proven to be capable of inducing ecologically valid overground walking gait and falls similar to those reported in the literature [6–9, 39, 40].

The usage of SPM1D as an analysis tool allowed researchers, for the first time ever, to perform exploratory time varying analyses of trip and slip reactive kinematics. SPM1D has also proven to be an invaluable statistical tool for visualising the changes in time-varying joint kinematics during various fall scenarios, and may make it possible to pinpoint the true cause of deficiency in balance-impaired subjects.

## Supplementary information

**Additional file 1.** FIMP transparency analyses. Analysis of FIMP transparency which concludes that the ankle strap has minimal impact on the individual's normal walking gait.

**Additional file 2.** Fall Compilation video. A compilation video of different subjects being induced with terminal swing trip, mid swing trip and slip. All subjects were walking at their preferred speed and were instructed to stand straight immediately after their recovery.

**Additional file 3.** Post Hoc analysis. Post hoc analysis of terminal swing (TS), mid swing (MS) and slip (SL) versus normal walking (NW) and strap walking (SW). These post hoc analyses were conducted after performing the ANOVA as shown in Figs. 10, 11, 13, 14, 16, 17. Bonferroni correction was used to adjust the alpha value for the multiple comparisons.

**Additional file 4.** Individual kinematic analysis. Individual kinematic analysis of the transparency and fall trials. These individual results are summarised in the fourth and last row of Figs. 10, 11, 13, 14, 16, 17.

## Data Availability

The dataset used and/or analysed during the current study are available from the corresponding author upon reasonable request.

## Abbreviations

ANOVA: Analysis of variance;
BoS: Base of support;
CoM: Center of mass;
FIMP: Fall inducing movable platform;
IMU: Inertial measurement unit;
MS: MidSwing trip;
MSE: Mean square of error;
NW: Normal walking;
PD: Proportional derivative;
SL: Slip;
SPM: Statistical parametric mapping;
SW: Strap walking;
TS: TerminalSwing trip
