# Supplementary material for: Fall inducing movable platform (FIMP) for overground trips and slips
Source: J Neuroeng Rehabil. 2020 Dec 3;17:161. doi: 10.1186/s12984-020-00785-0 (PMC7713354; doi:10.1186/s12984-020-00785-0)
Supplement: Supplementary file 1 — Additional file 1. FIMP transparency analyses. Analysis of FIMP transparency which concludes that the ankle strap has minimal impact on the individual's normal walking gait. [file 12984_2020_785_MOESM1_ESM.pdf]

Fall Inducing Movable Platform (FIMP) Transparency

This section investigates the transparency of the FIMP for normal walking gait. An ideal fall inducing system should not affect the normal walking gait of the subject under any conditions. The FIMP requires subjects to be attached to its fall inducing mechanisms via an ankle strap connected to ankle cables, which may affect their normal walking gait. The impact of the ankle strap's pulling forces during normal walking is evaluated as FIMP transparency.

The normal walking (NW, no ankle straps and no safety harness) trials were compared to the strap walking (SW, walking with straps and safety harness) trials in order to discern the influence, if any, the FIMP may have on the subject's walking gait. For this analysis, time varying sagittal plane hip, knee and ankle joint kinematics were compared using 1-Dimensional Statistical Parametric Mapping (SPM1D).

Two sample t-tests were conducted with SPM1D analysis with an alpha threshold of 0.05. The level of significance found was separated into intervals of 1 standard deviation as t-tests calculate the relative difference between 2 groups over their combined standard error of mean. The mean relative effect (combined from all trials) and the individual relative effect of NW versus SW SPM1D{t} are also presented in the form of colour maps to visualise the regions where statistical differences were found to have occurred (SFig. 1 and SFig. 2). A rainbow scale was used, with redder colours denoting more significant differences.

Left Leg (Transparency Analyses)

For the left leg, results indicate little to no difference in the kinematics of hip and knee flexion between NW and SW (SFig. 1). This implies that the ankle straps and safety harness did not affect the movement of the thigh and shank in a meaningful way. For ankle plantar/ dorsi flexion kinematics, significant differences were found, though the magnitude of the effect was not consistent across all subjects. The excess dorsi flexion and was found to have occurred during mid-swing and hence should not generate appreciable internal or external forces or torques on the subject, and should have minimal effect on balance recovery during fall events. A minor side-effect may be a slight increase in fatigue on the muscles responsible for dorsiflexion (e.g. tibialis anterior).

Nevertheless, it will still be ideal to achieve greater FIMP transparency and hence a greater resemblance to normal walking. Several factors may contribute to the observed differences in ankle plantar/ dorsiflexion. First, the act of attaching the ankle strap requires interaction with the reflective markers on the ankle which may have affected their calibrated alignment. Second, the ankle strap is liable to move with the plantar/ dorsi flexion during the swing phase, and may thus have affected the position of the malleolus markers which were used to define the position of the ankle joint centre. Third, the tension force on the ankle strap may have required larger dorsi flexion to maintain a certain toe clearance height. Finally, the tightness of the ankle straps could have prevented the normal functioning of the ankle. Further work is required to determine the causal factors underpinning the observed differences. This may include engineering work into improving the force characteristics of the power springs, and the use of better methods to control the tightness of the ankle straps.

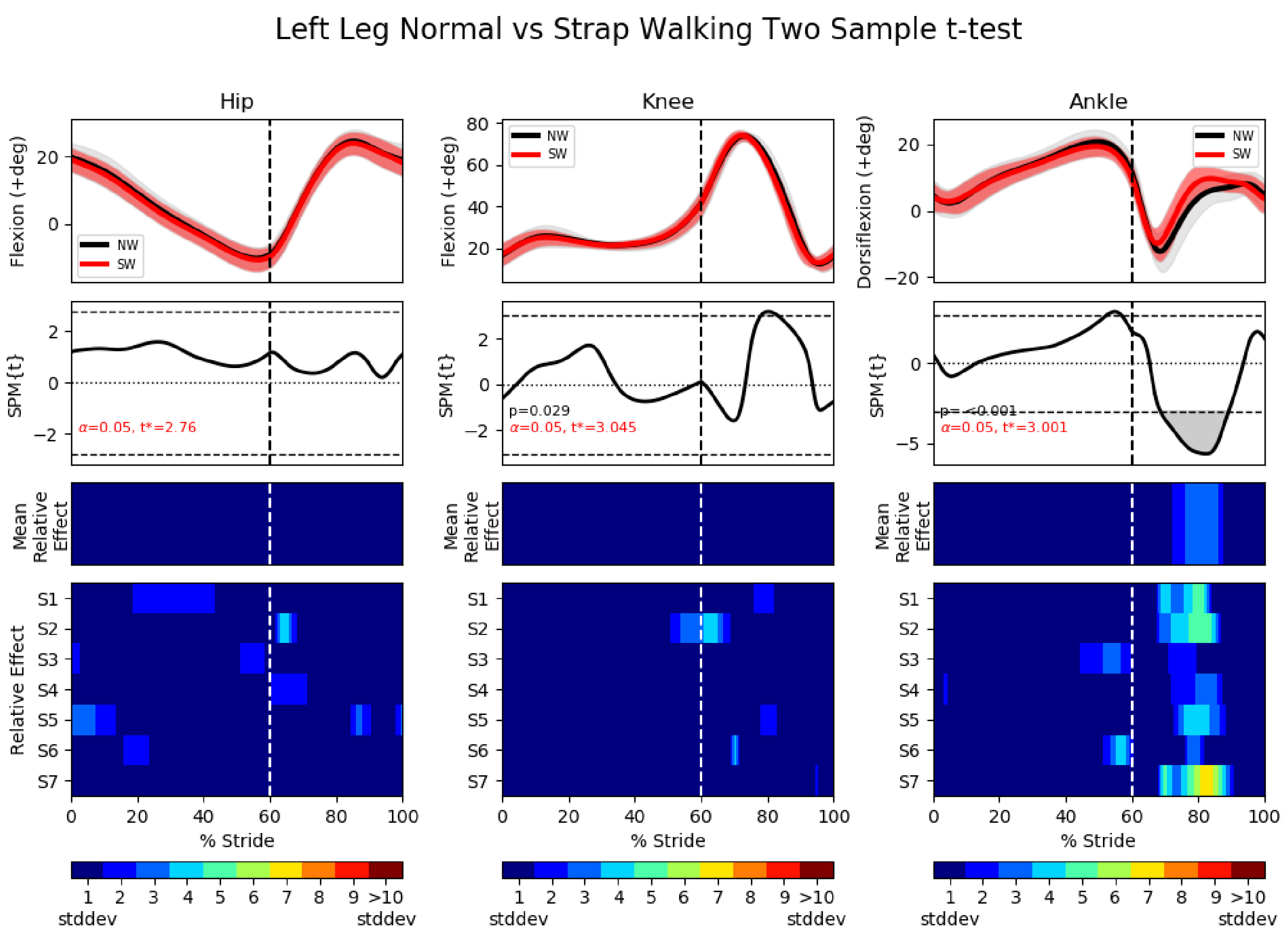

Supplementary Figure 1 Comparison of NormalWalking (NW) vs StrapWalking (SW) trials for the left leg using SPM1D and t-test. The ankle strap was worn on the left leg for fall induction. The top row of graphs show the mean and standard deviation clouds for the hip, knee and ankle flexion angles for all subjects; mean ($\pm$ st.dev). The second row of graphs plot the two-sample t-test values for the comparison in the top row. The third and last rows show colour maps highlighting the significance of the t-test results for all subjects combined, and for individual subjects (labelled S1 through S7). Stance phase is located to the left of the black vertical dotted line and swing phase to the right. Significant differences can be seen in the ankle joint during the mid-swing phase for a majority of the subjects. Differences were also found in the knee during the mid-swing phase, but less consistently across all of the subjects.

Right Leg (Transparency Analyses)

For the right leg which did not have any straps attached, no significant differences were found between NW and SW for all lower limb joints (SFig. 2). This implies that the use of the safety harness anchored at the top crossbeam on the FIMP did not affect the gait of the subject, and the method of using subject follower algorithm to reduce harness inertia is considered a success.

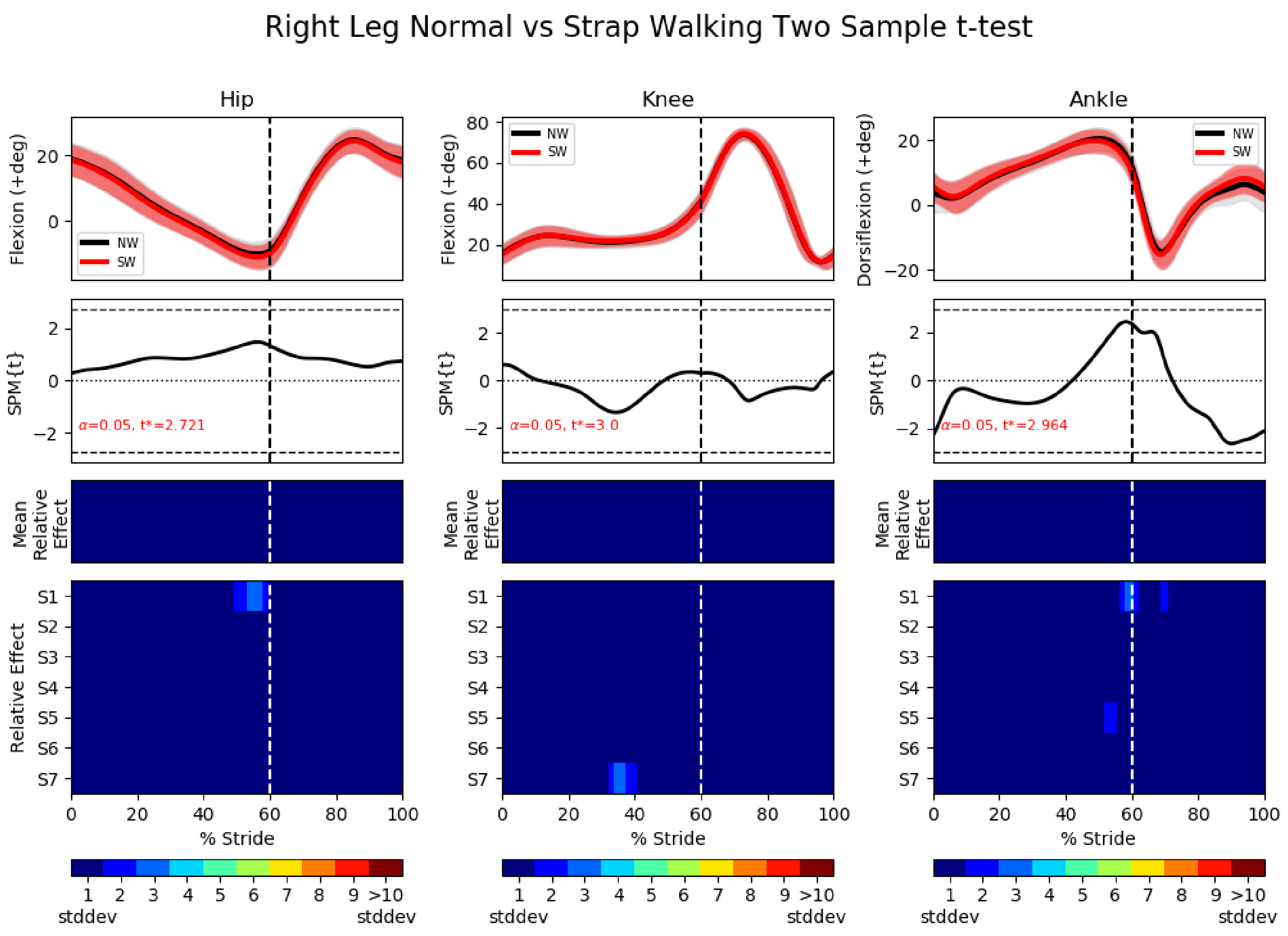

Supplementary Figure 2 Comparison of NormalWalking (NW) vs StrapWalking (SW) trials for the right leg using SPM1D and t-test. No ankle straps were worn on the right leg. The top row of graphs show the mean and the standard deviation clouds for the hip, knee and ankle flexion angles for all subjects; mean ($\pm$ st.dev). The second row of graphs plot the two-sample t-test values for the comparison in the top row. The third and last rows show colour maps highlighting the significance of the t-test results for all subjects combined, and for individual subjects (labelled S1 through S7). Stance phase is located to the left of the black vertical dotted line and swing phase to the right. No significant differences were found in any of the joints for the majority of the subjects.
