# Supplementary material for: Fall inducing movable platform (FIMP) for overground trips and slips
Source: J Neuroeng Rehabil. 2020 Dec 3;17:161. doi: 10.1186/s12984-020-00785-0 (PMC7713354; doi:10.1186/s12984-020-00785-0)

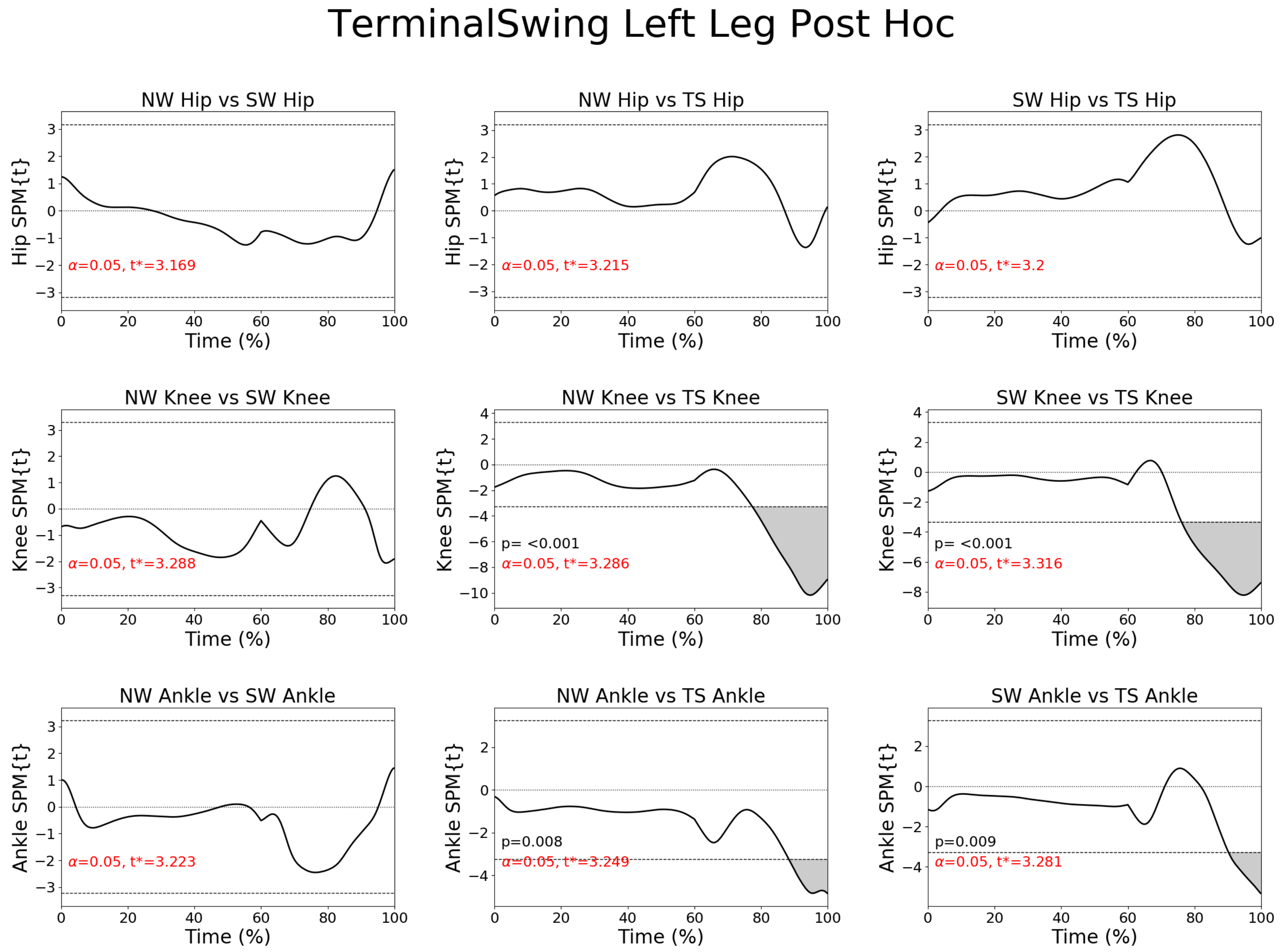

TerminalSwing Left Leg Post Hoc
NW Hip vs SW Hip
α=0.05, t*=3.169
NW Hip vs TS Hip
α=0.05, t*=3.215
SW Hip vs TS Hip
α=0.05, t*=3.2
NW Knee vs SW Knee
α=0.05, t*=3.288
NW Knee vs TS Knee
p= <0.001
α=0.05, t*=3.286
SW Knee vs TS Knee
p= <0.001
α=0.05, t*=3.316
NW Ankle vs SW Ankle
α=0.05, t*=3.223
NW Ankle vs TS Ankle
p=0.008
α=0.05, t*=3.249
SW Ankle vs TS Ankle
p=0.009
α=0.05, t*=3.281
Hip SPM{t}
Knee SPM{t}
Ankle SPM{t}
Time (%)

TerminalSwing Right Leg Post Hoc

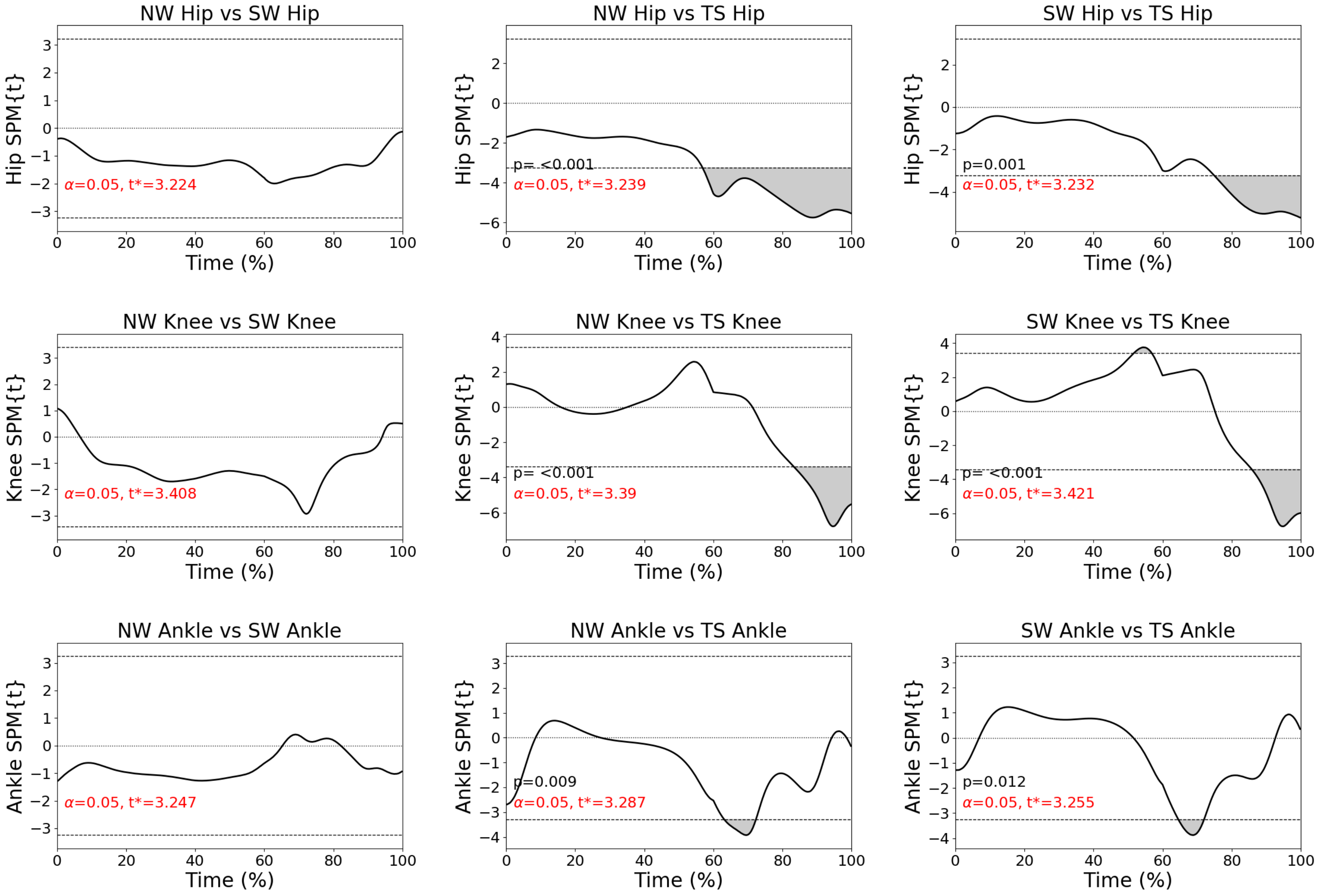

MidSwing Left Leg Post Hoc

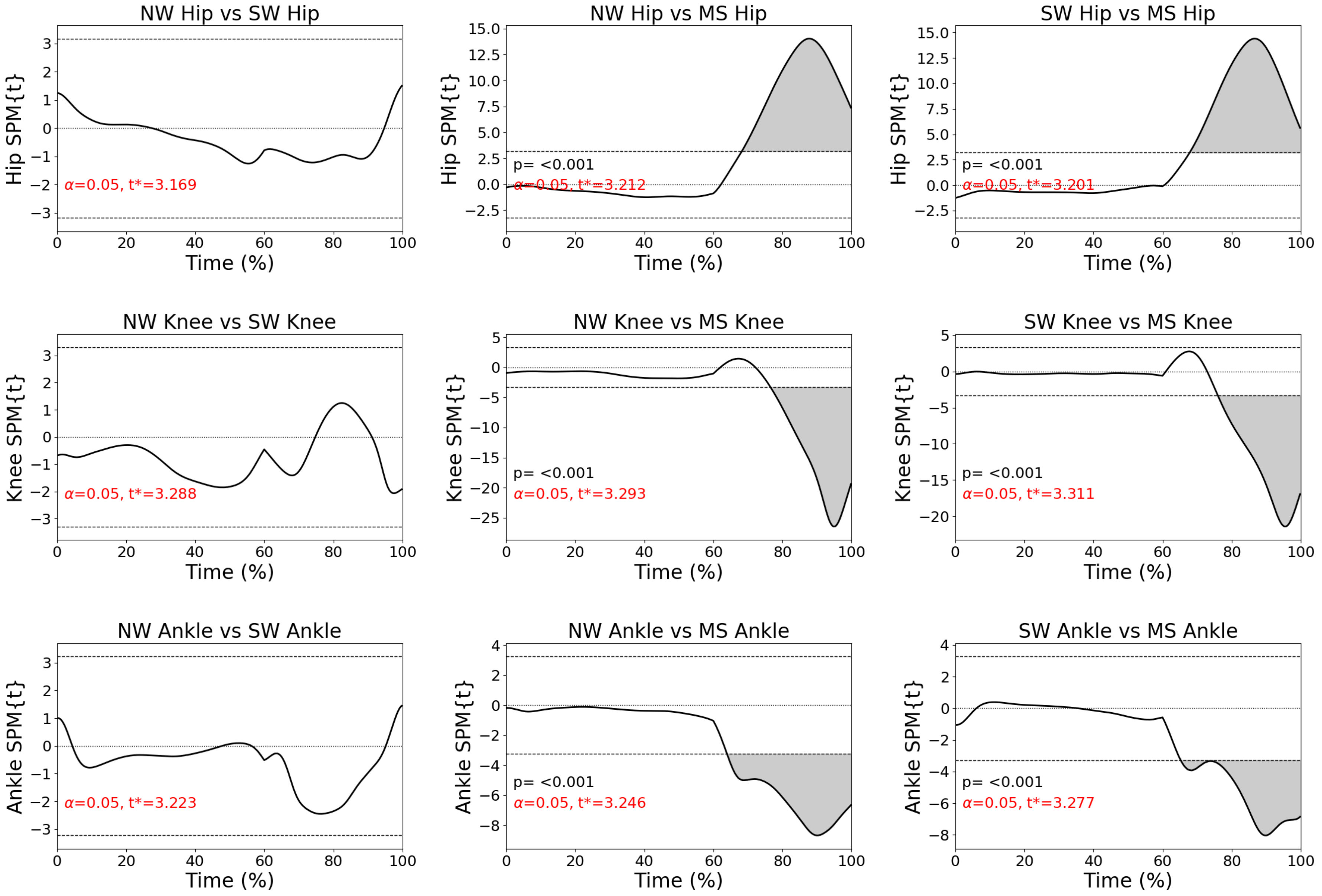

MidSwing Right Leg Post Hoc

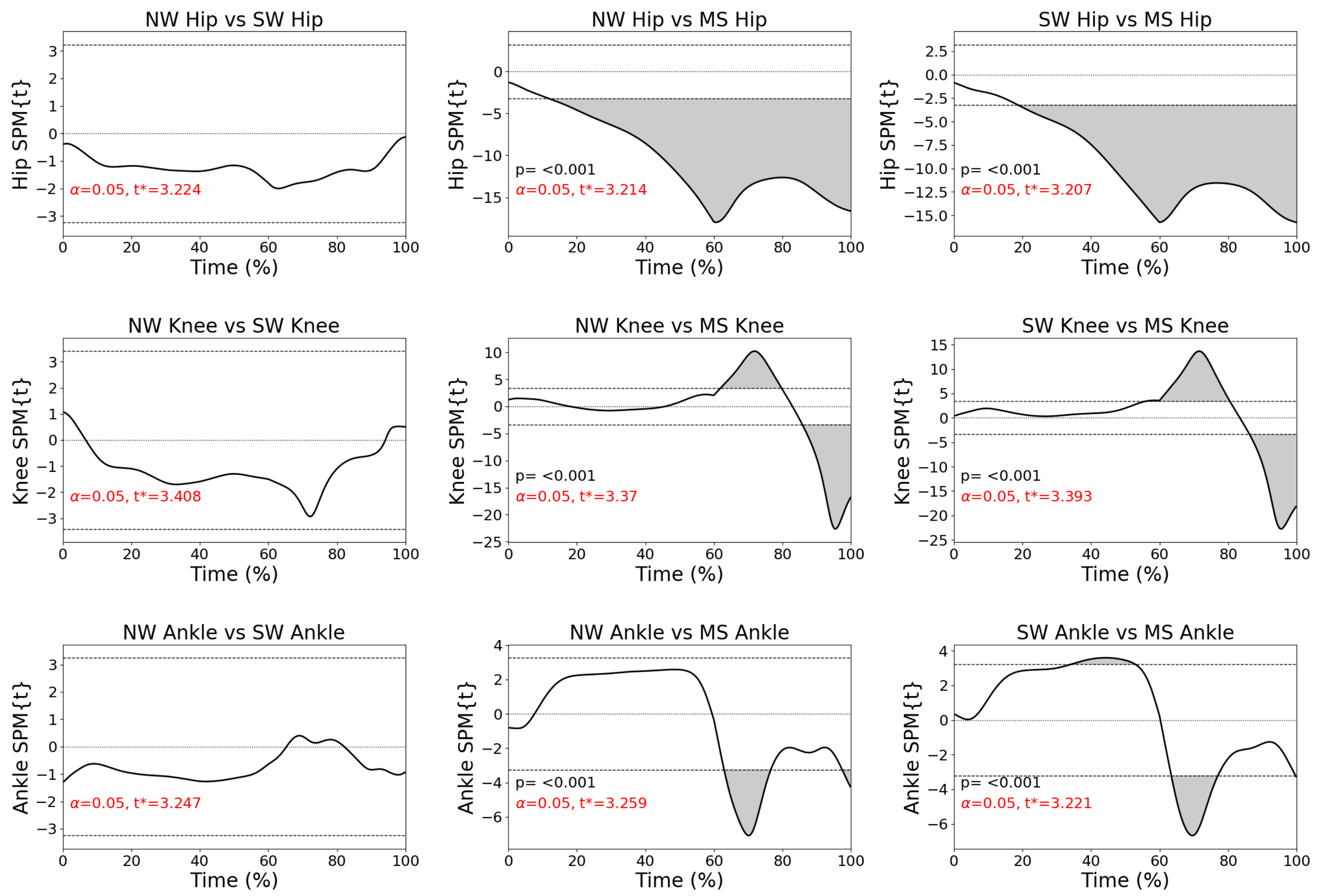

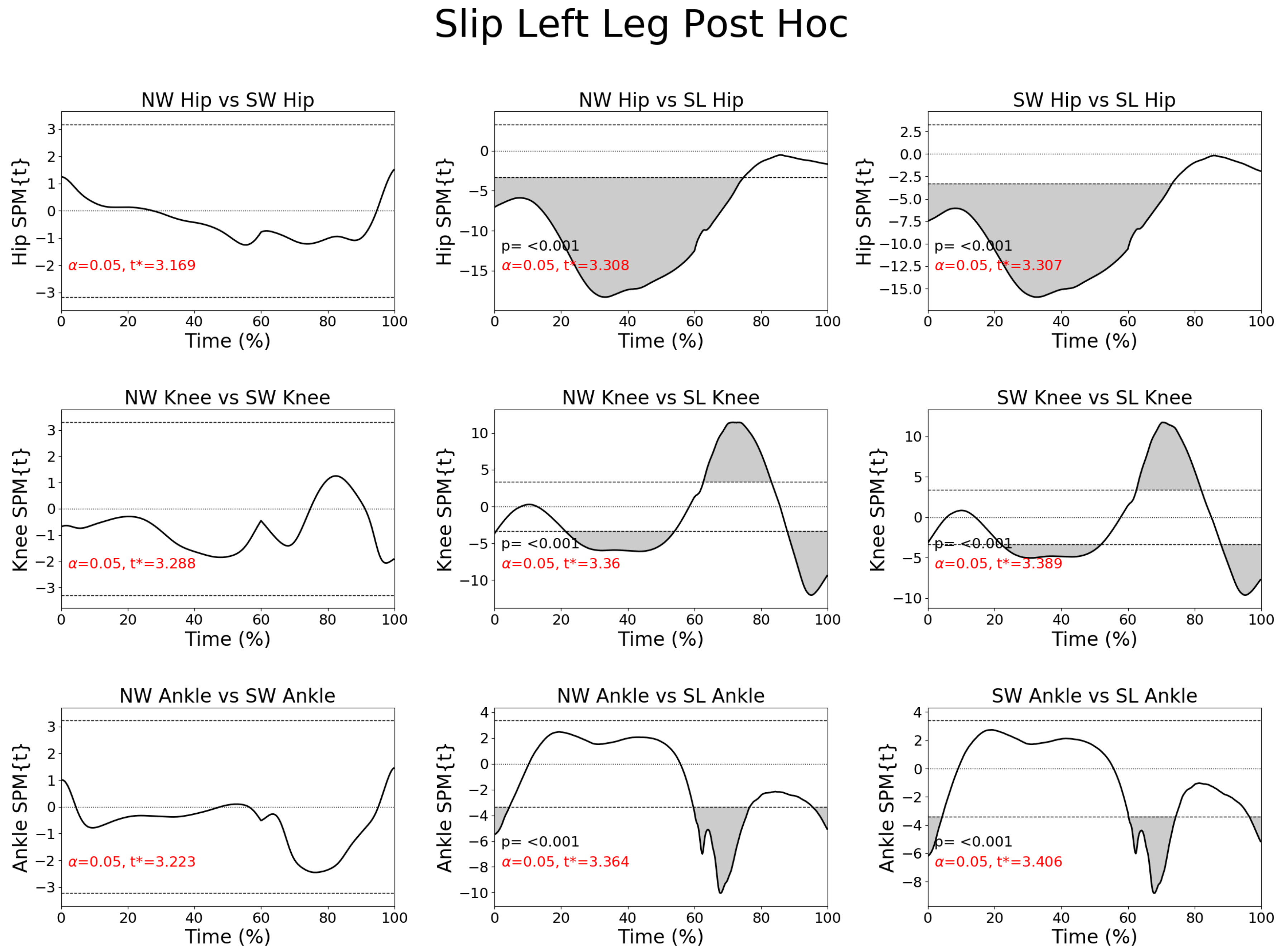

Slip Left Leg Post Hoc
NW Hip vs SW Hip
α=0.05, t*=3.169
NW Hip vs SL Hip
p= <0.001
α=0.05, t*=3.308
SW Hip vs SL Hip
p= <0.001
α=0.05, t*=3.307
Hip SPM{t}
NW Knee vs SW Knee
α=0.05, t*=3.288
NW Knee vs SL Knee
p= <0.001
α=0.05, t*=3.36
SW Knee vs SL Knee
p= <0.001
α=0.05, t*=3.389
Knee SPM{t}
NW Ankle vs SW Ankle
α=0.05, t*=3.223
NW Ankle vs SL Ankle
p= <0.001
α=0.05, t*=3.364
SW Ankle vs SL Ankle
p= <0.001
α=0.05, t*=3.406
Ankle SPM{t}
Time (%)

Slip Right Leg Post Hoc

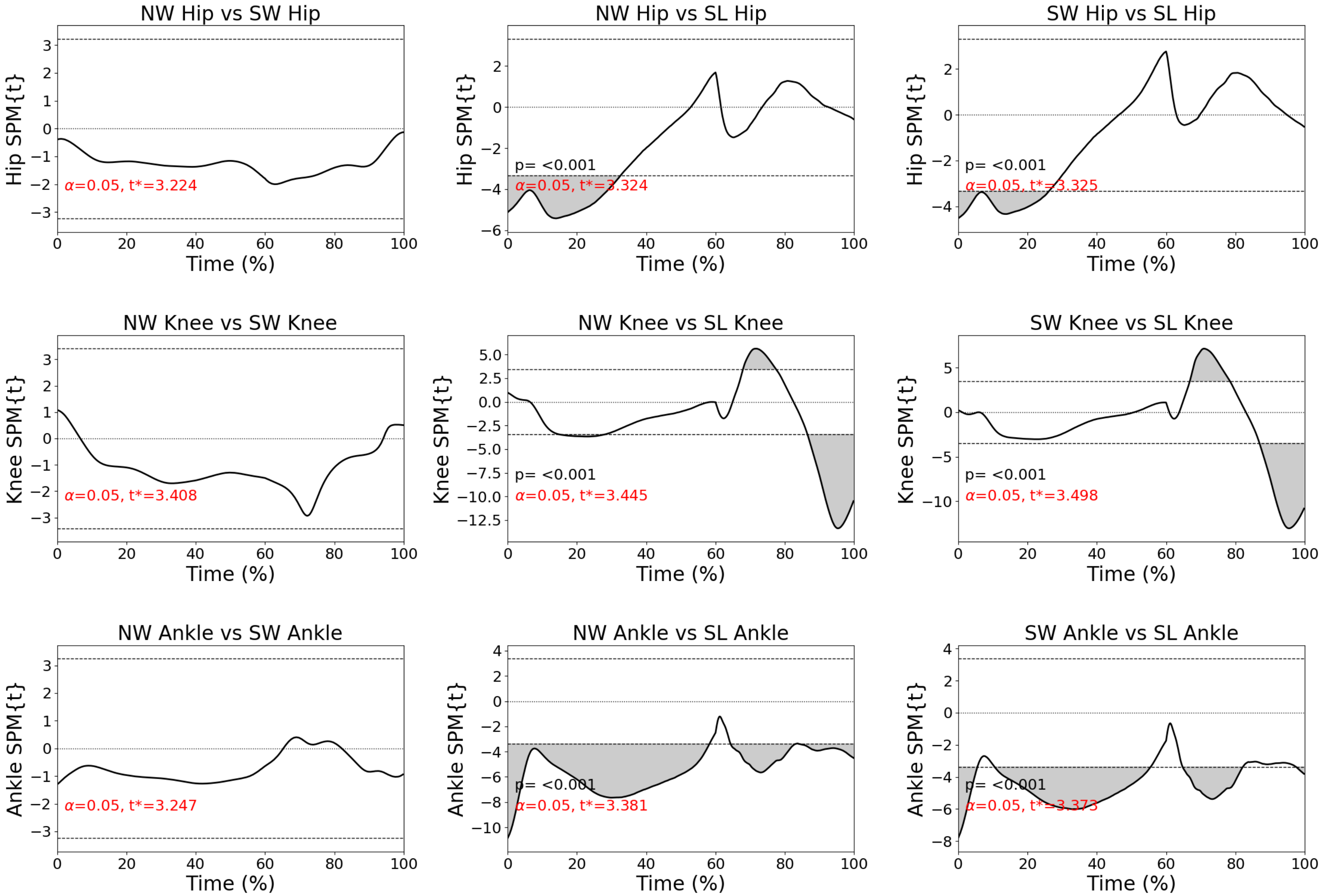

Supplement: Supplementary file 3 — Additional file 3. Post Hoc analysis. Post hoc analysis of terminal swing (TS), mid swing (MS) and slip (SL) versus normal walking (NW) and strap walking (SW). These post hoc analyses were conducted after performing the ANOVA as shown in Figs. 10, 11, 13, 14, 16, 17. Bonferroni correction was used to adjust the alpha value for the multiple comparisons. [file 12984_2020_785_MOESM3_ESM.pdf]
