# Supplementary material for: Fall inducing movable platform (FIMP) for overground trips and slips
Source: J Neuroeng Rehabil. 2020 Dec 3;17:161. doi: 10.1186/s12984-020-00785-0 (PMC7713354; doi:10.1186/s12984-020-00785-0)
Supplement: Supplementary file 4 — Additional file 4. Individual kinematic analysis. Individual kinematic analysis of the transparency and fall trials. These individual results are summarised in the fourth and last row of Figs. 10, 11, 13, 14, 16, 17. [file 12984_2020_785_MOESM4_ESM.pdf]

FIMP Transparency (Left leg)

Subject 1 to 7

Comparing normal walking without ankle strap (NW) to walking with ankle strap attached to fall mechanism (SW)

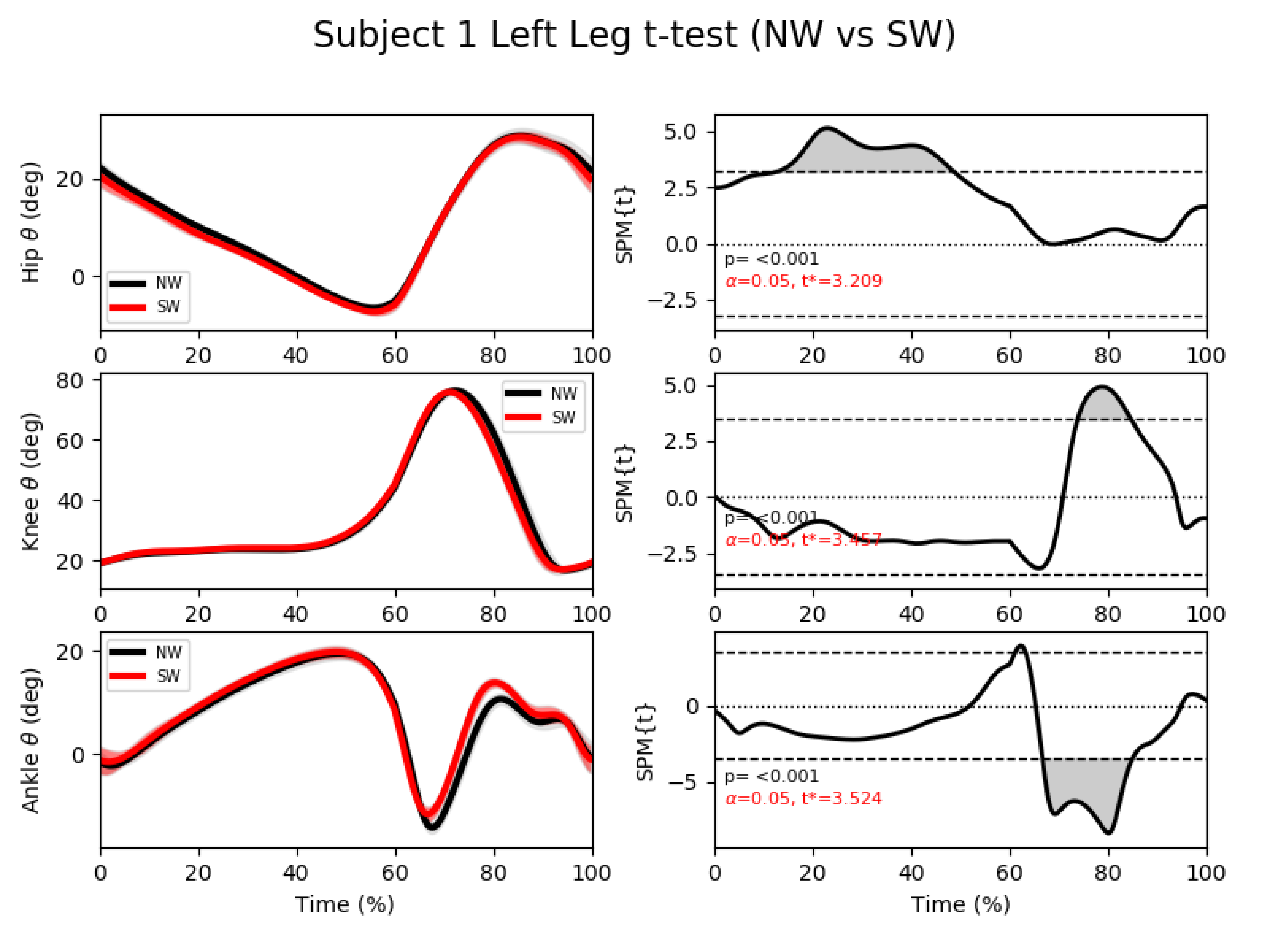
Subject 1 Left Leg t-test (NW vs SW)
Hip θ (deg)
Knee θ (deg)
Ankle θ (deg)
NW
SW
SPM{t}
p= <0.001
α=0.05, t*=3.209
α=0.05, t*=3.457
α=0.05, t*=3.524
Time (%)

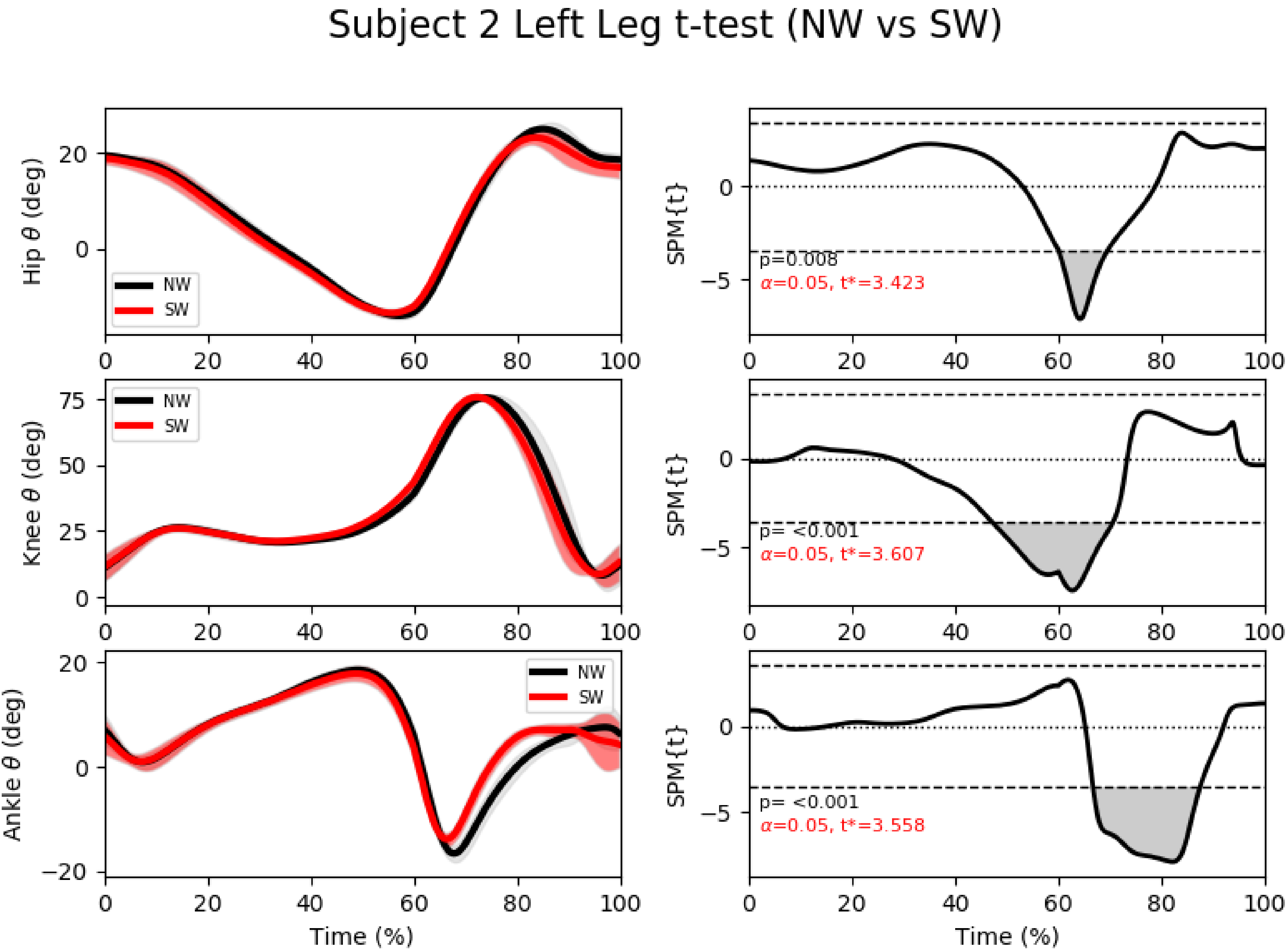
Subject 2 Left Leg t-test (NW vs SW)
Hip θ (deg)
Knee θ (deg)
Ankle θ (deg)
NW
SW
SPM{t}
p=0.008
α=0.05, t*=3.423
p= <0.001
α=0.05, t*=3.607
p= <0.001
α=0.05, t*=3.558
Time (%)

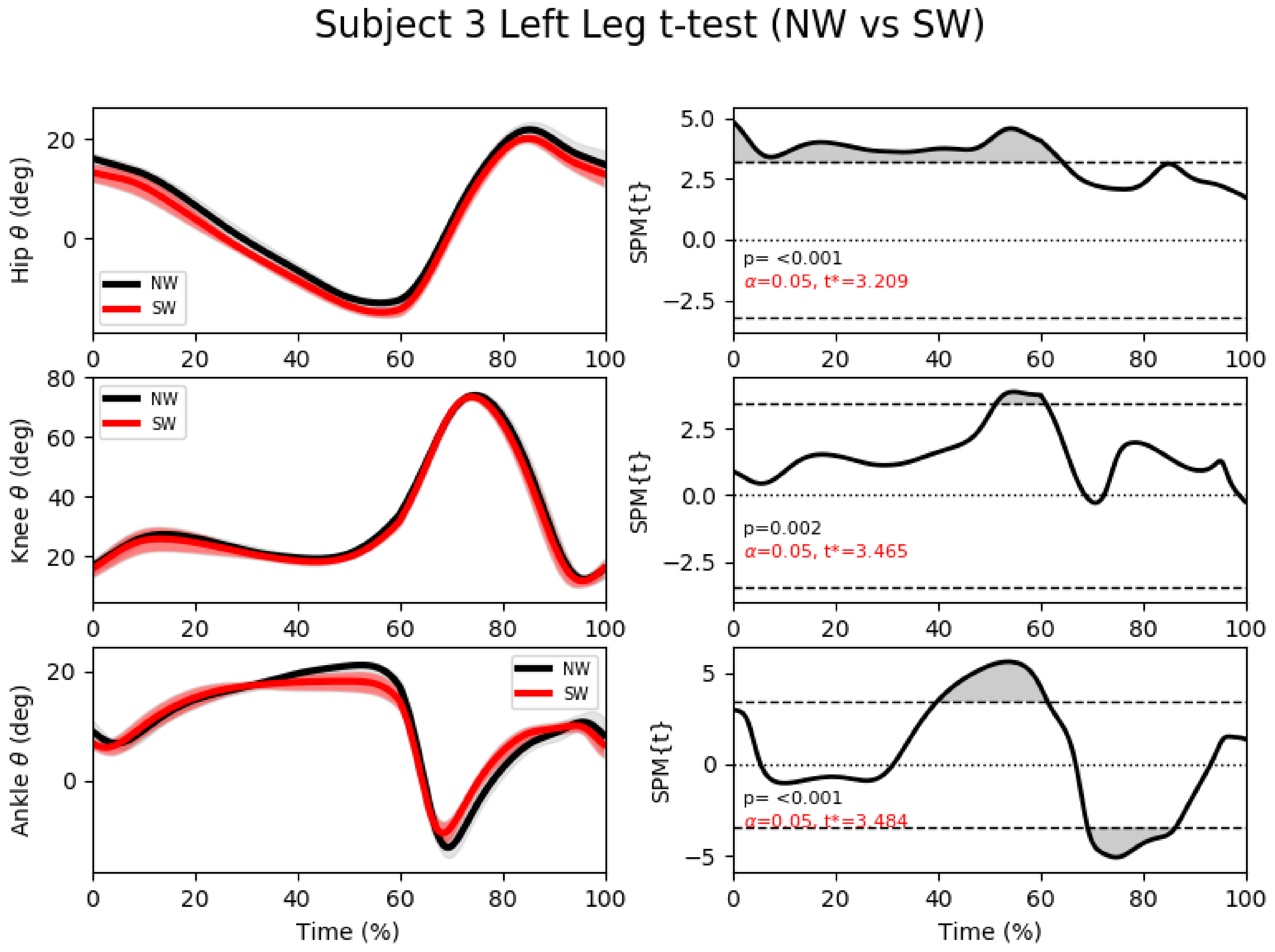
Subject 3 Left Leg t-test (NW vs SW)
Hip θ (deg)
NW
SW
SPM{t}
p= <0.001
α=0.05, t*=3.209
Knee θ (deg)
p=0.002
α=0.05, t*=3.465
Ankle θ (deg)
p= <0.001
α=0.05, t*=3.484
Time (%)

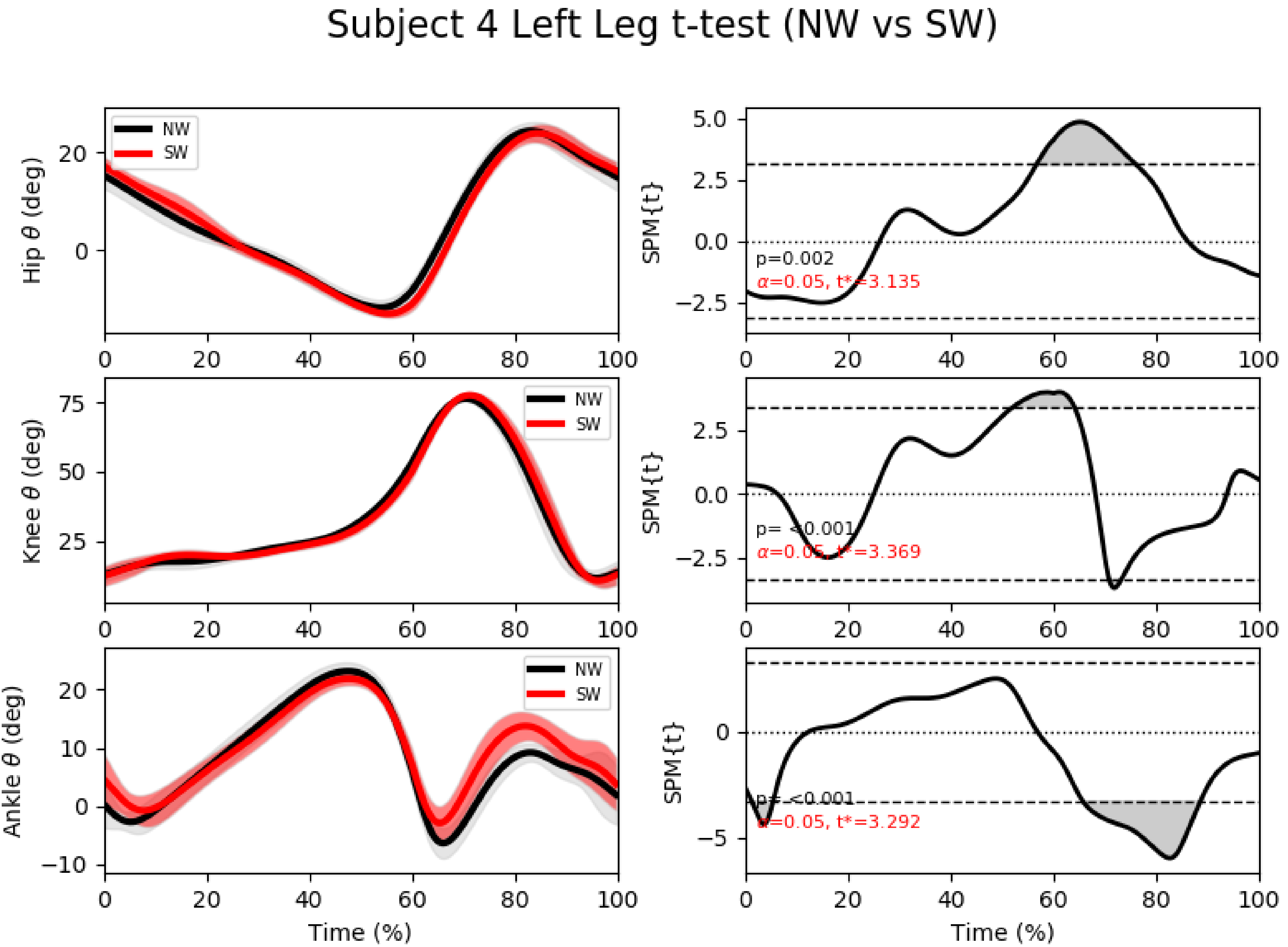
Subject 4 Left Leg t-test (NW vs SW)
NW
SW
Hip θ (deg)
SPM{t}
p=0.002
α=0.05, t*=3.135
Knee θ (deg)
p= <0.001
α=0.05, t*=3.369
Ankle θ (deg)
p= <0.001
α=0.05, t*=3.292
Time (%)
Time (%)

Subject 5 Left Leg t-test (NW vs SW)

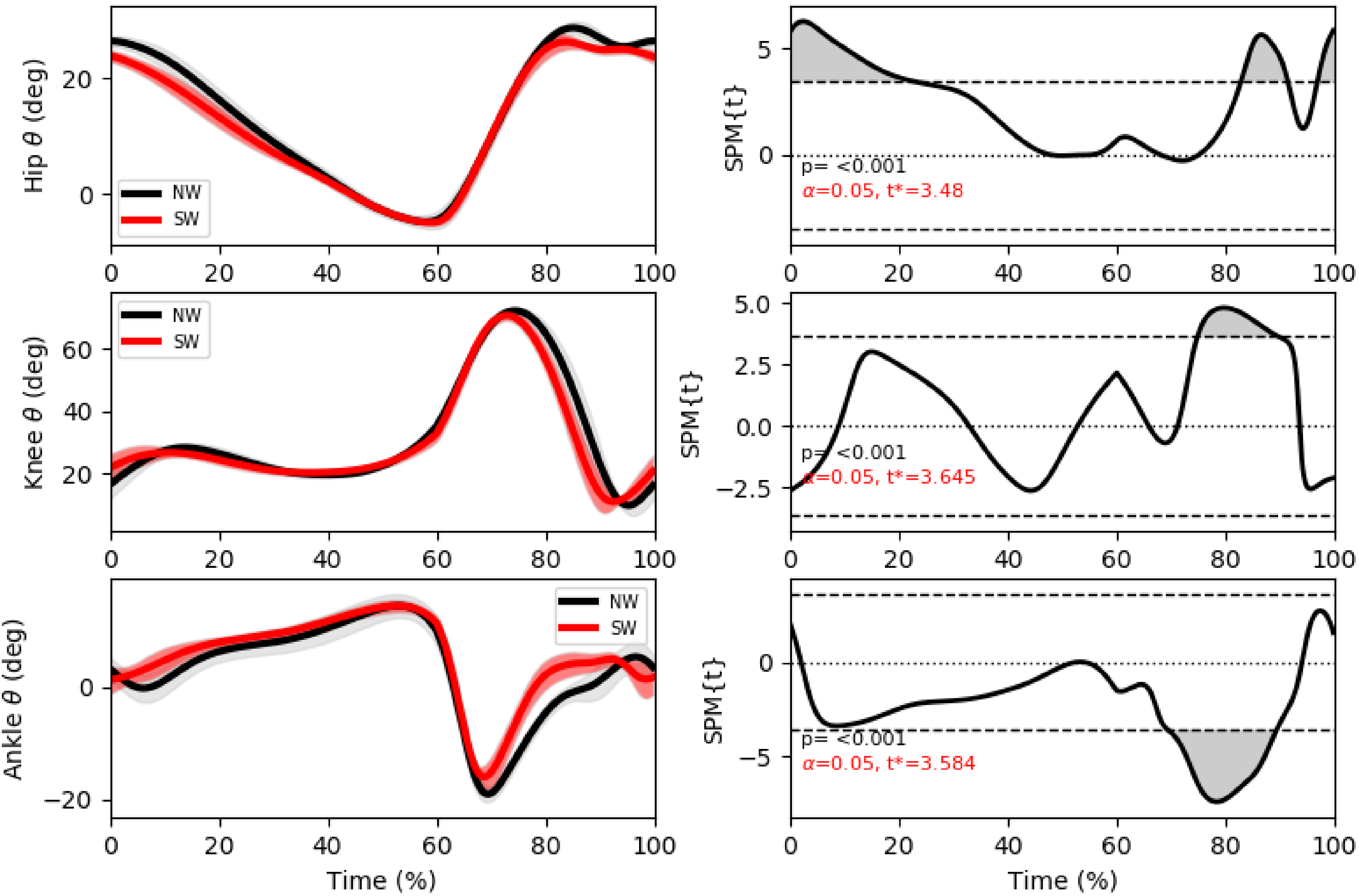

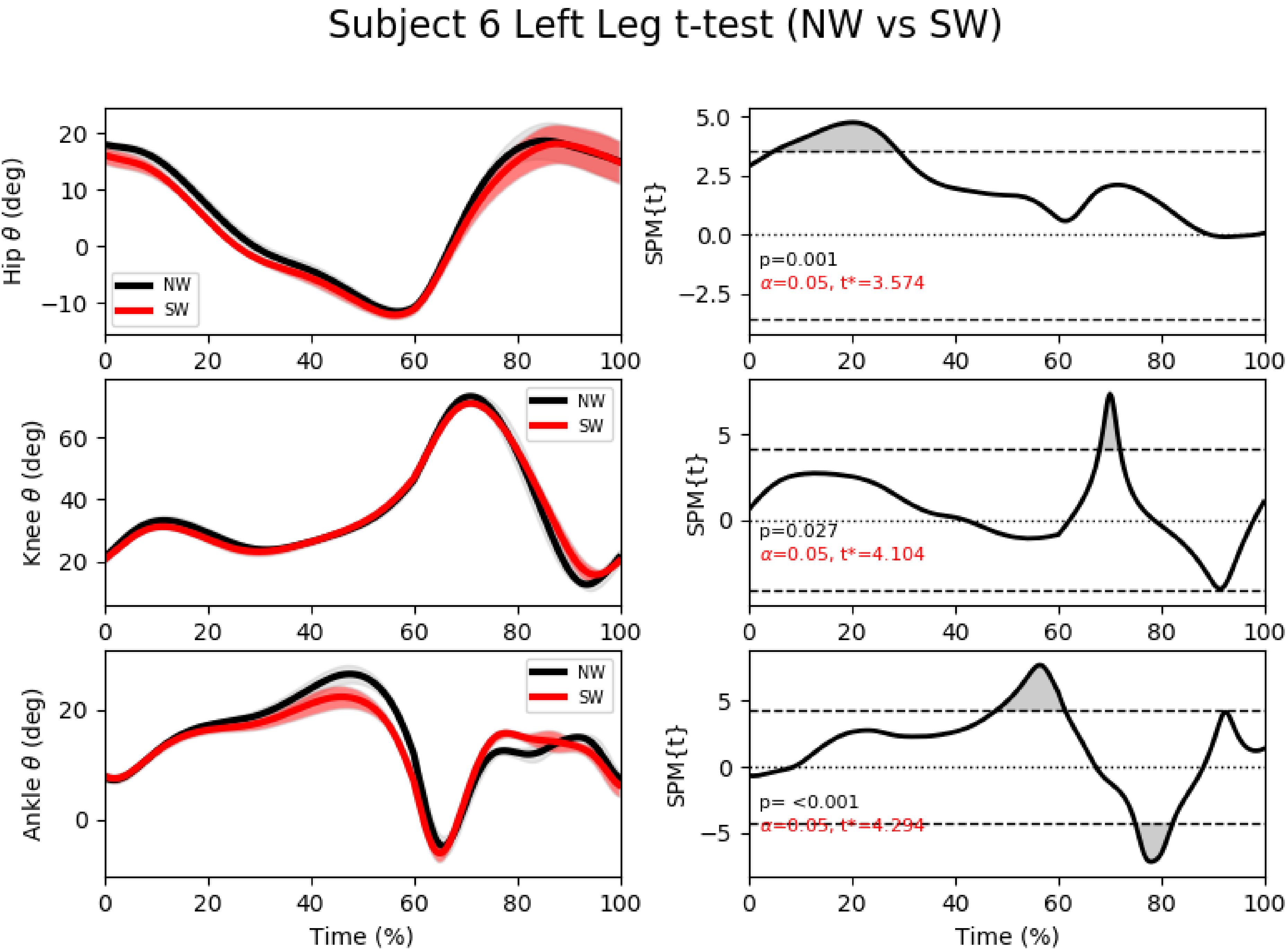
Subject 6 Left Leg t-test (NW vs SW)
Hip θ (deg)
NW
SW
SPM{t}
p=0.001
α=0.05, t*=3.574
Knee θ (deg)
p=0.027
α=0.05, t*=4.104
Ankle θ (deg)
p= <0.001
α=0.05, t*=4.294
Time (%)
Time (%)

Subject 7 Left Leg t-test (NW vs SW)

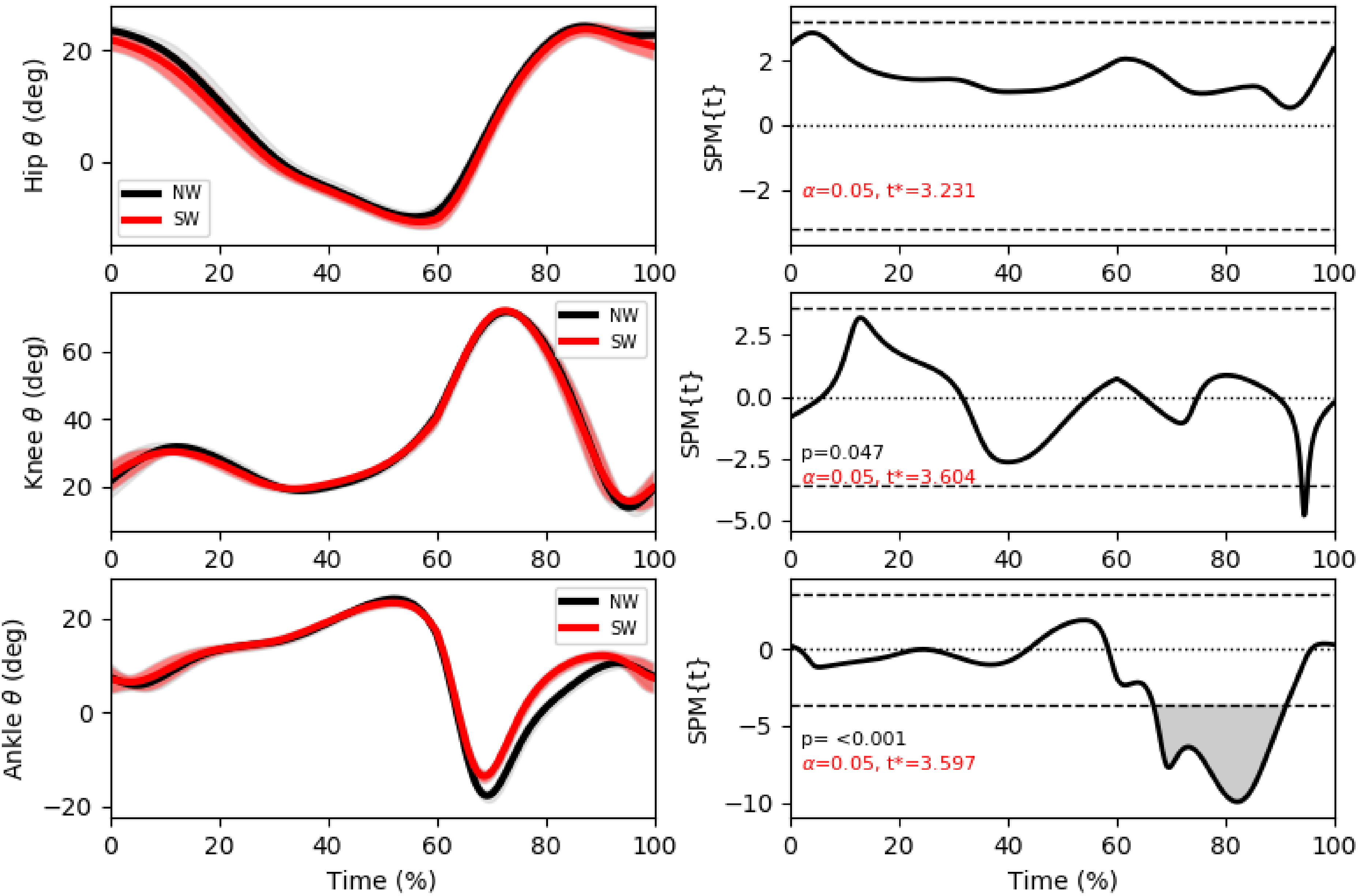

FIMP Transparency (Right leg)

Subject 1 to 7

Comparing normal walking without ankle strap (NW) to walking with ankle strap attached to fall mechanism (SW)

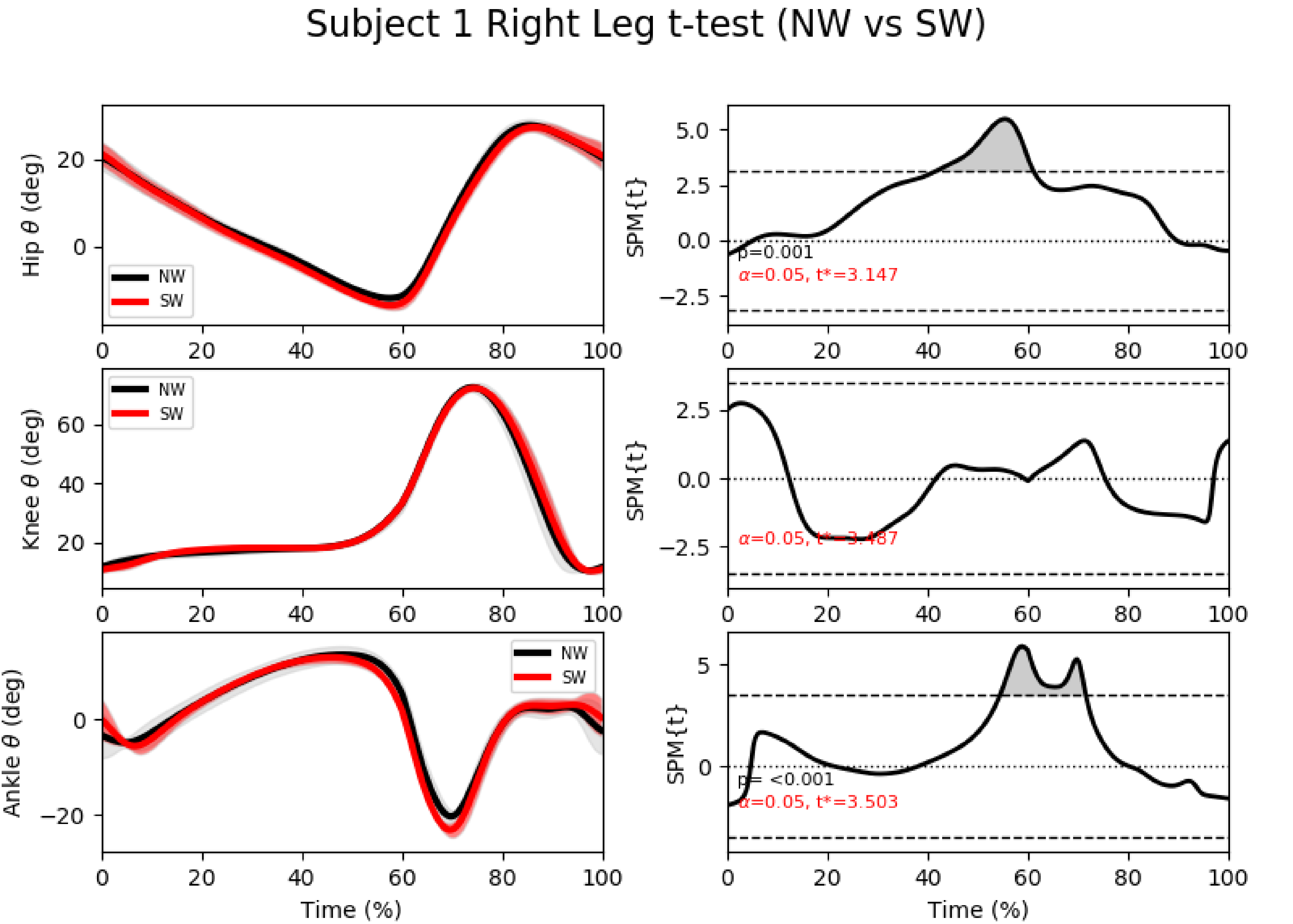
Subject 1 Right Leg t-test (NW vs SW)
Hip θ (deg)
NW
SW
SPM{t}
p=0.001
α=0.05, t*=3.147
Knee θ (deg)
α=0.05, t*=3.487
Ankle θ (deg)
p= <0.001
α=0.05, t*=3.503
Time (%)
Time (%)

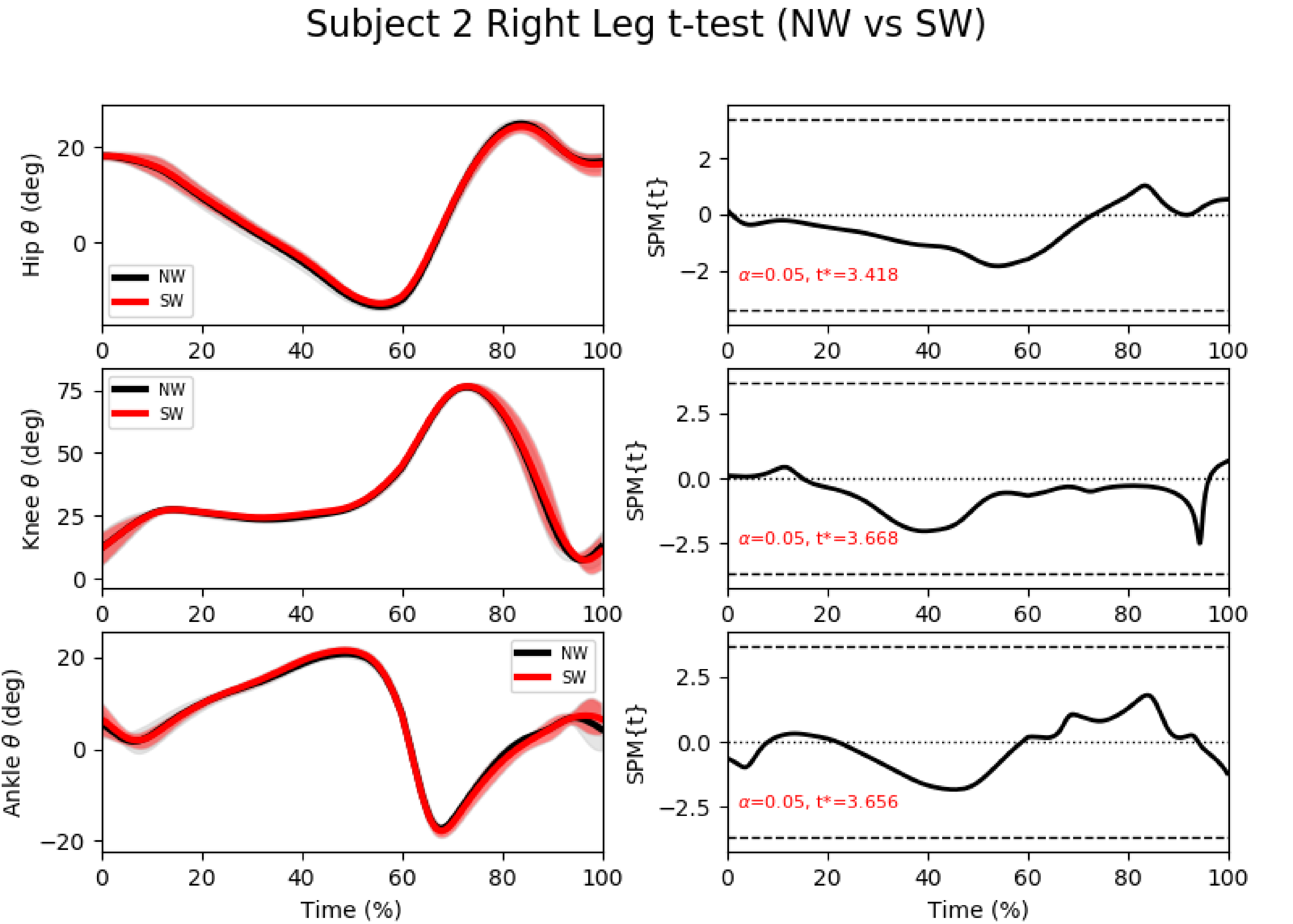
Subject 2 Right Leg t-test (NW vs SW)
Hip θ (deg)
NW
SW
SPM{t}
α=0.05, t*=3.418
Knee θ (deg)
α=0.05, t*=3.668
Ankle θ (deg)
α=0.05, t*=3.656
Time (%)
Time (%)

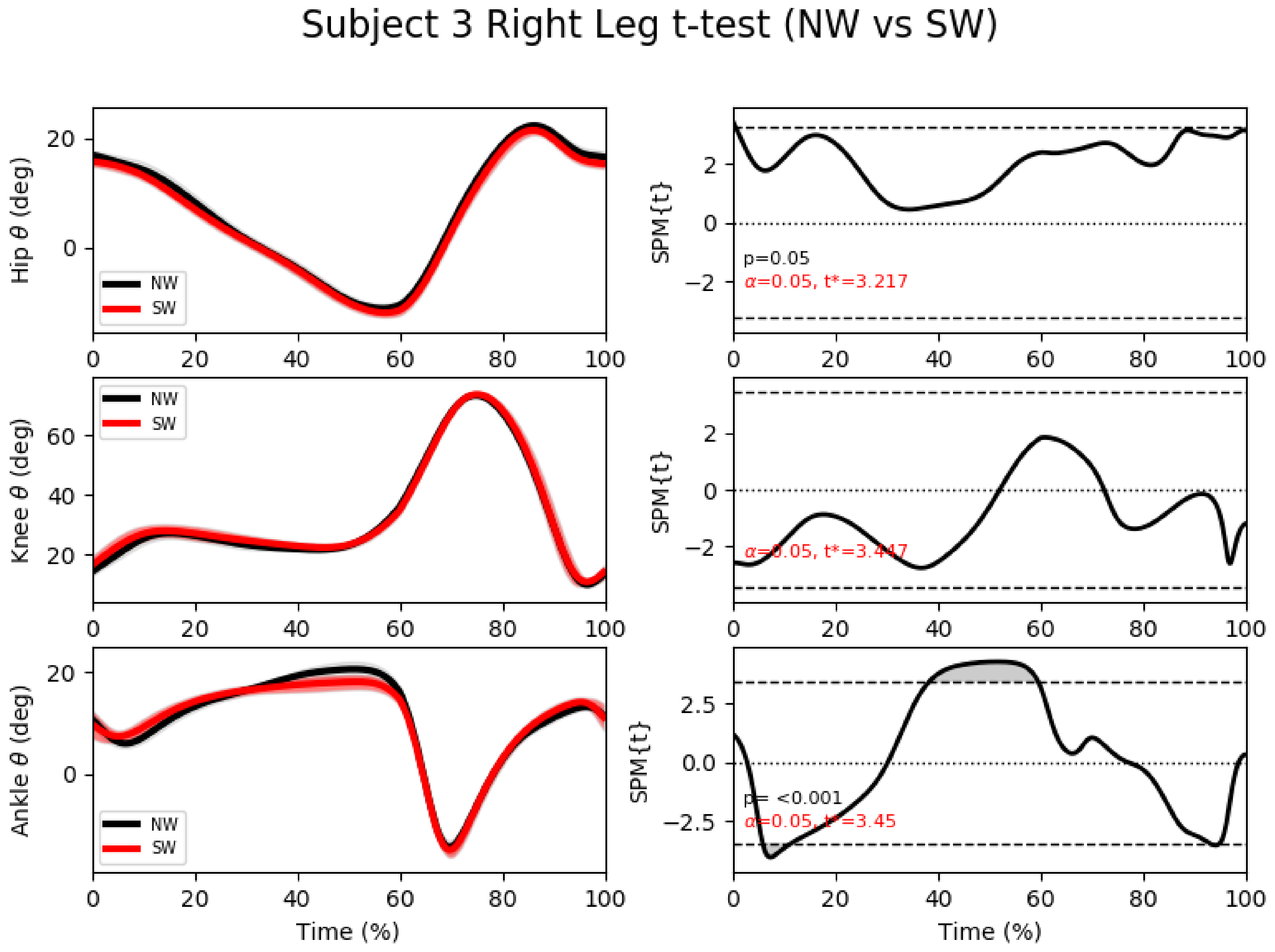

Subject 3 Right Leg t-test (NW vs SW)
Hip θ (deg)
Knee θ (deg)
Ankle θ (deg)
NW
SW
SPM{t}
p=0.05
α=0.05, t*=3.217
α=0.05, t*=3.447
p= <0.001
α=0.05, t*=3.45
Time (%)

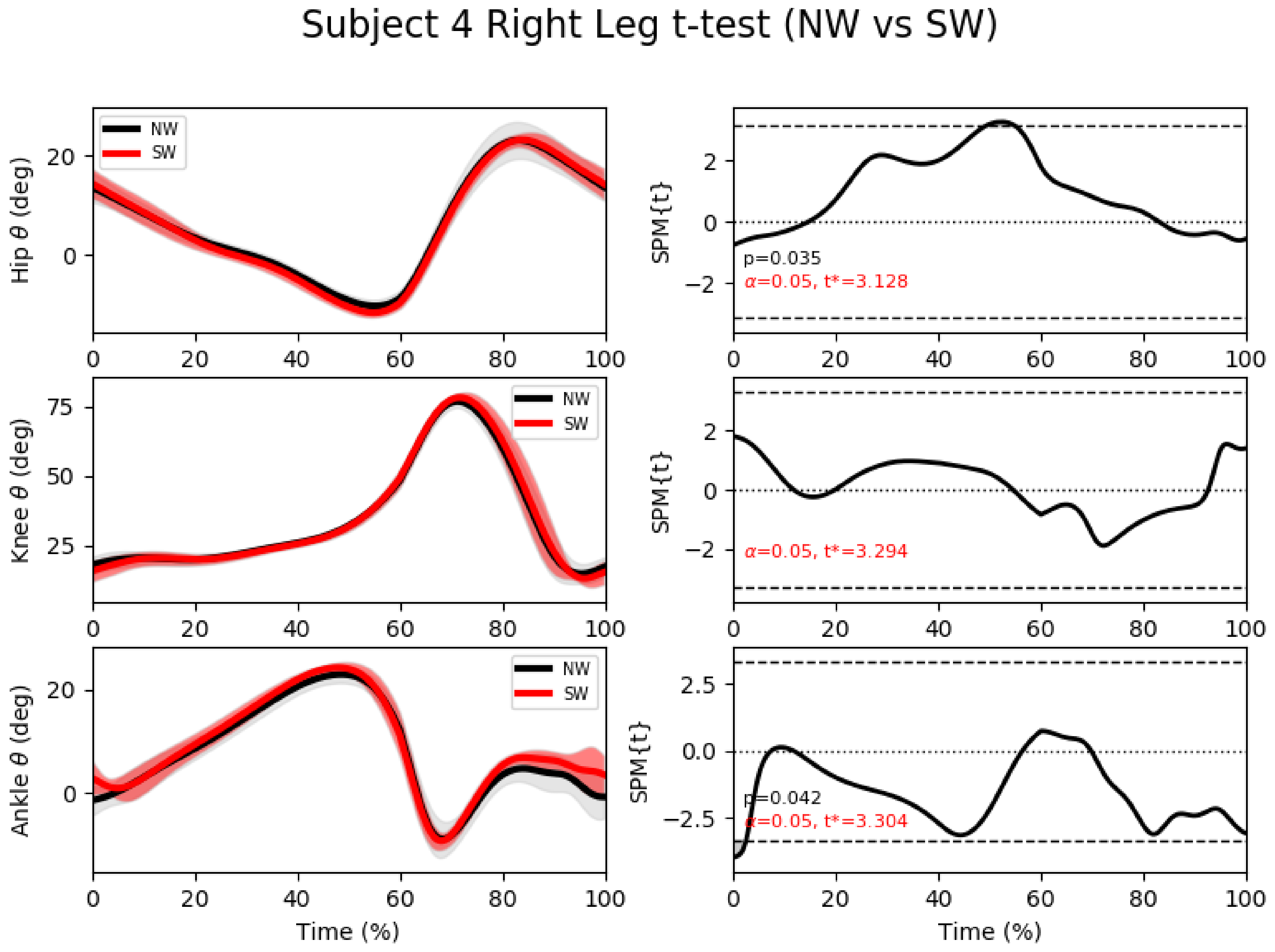

Subject 4 Right Leg t-test (NW vs SW)
Hip θ (deg)
Knee θ (deg)
Ankle θ (deg)
NW
SW
SPM{t}
p=0.035
α=0.05, t*=3.128
α=0.05, t*=3.294
p=0.042
α=0.05, t*=3.304
Time (%)

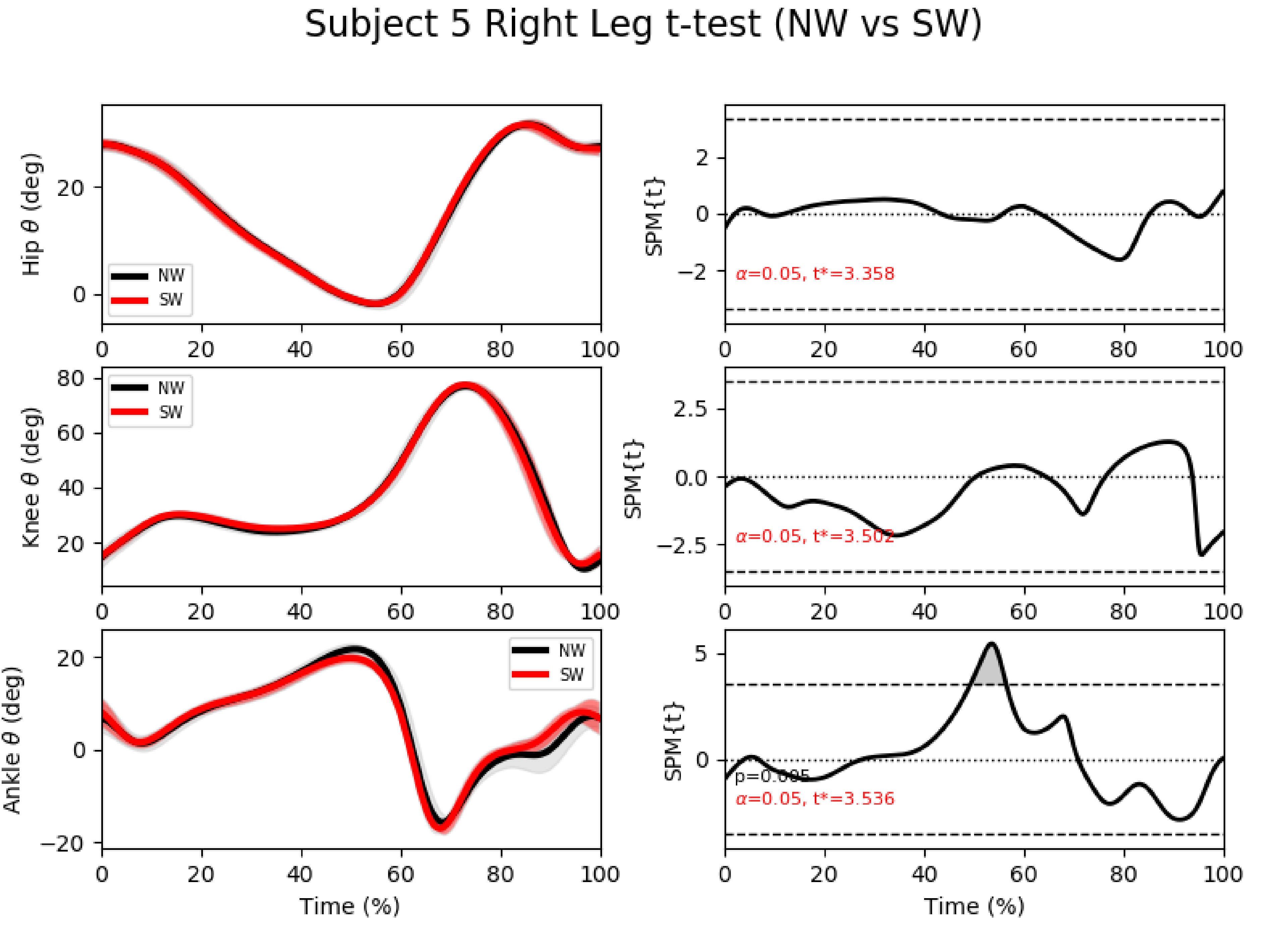

Subject 5 Right Leg t-test (NW vs SW)
Hip θ (deg)
NW
SW
SPM{t}
α=0.05, t*=3.358
Knee θ (deg)
NW
SW
SPM{t}
α=0.05, t*=3.502
Ankle θ (deg)
NW
SW
SPM{t}
p=0.005
α=0.05, t*=3.536
Time (%)
Time (%)

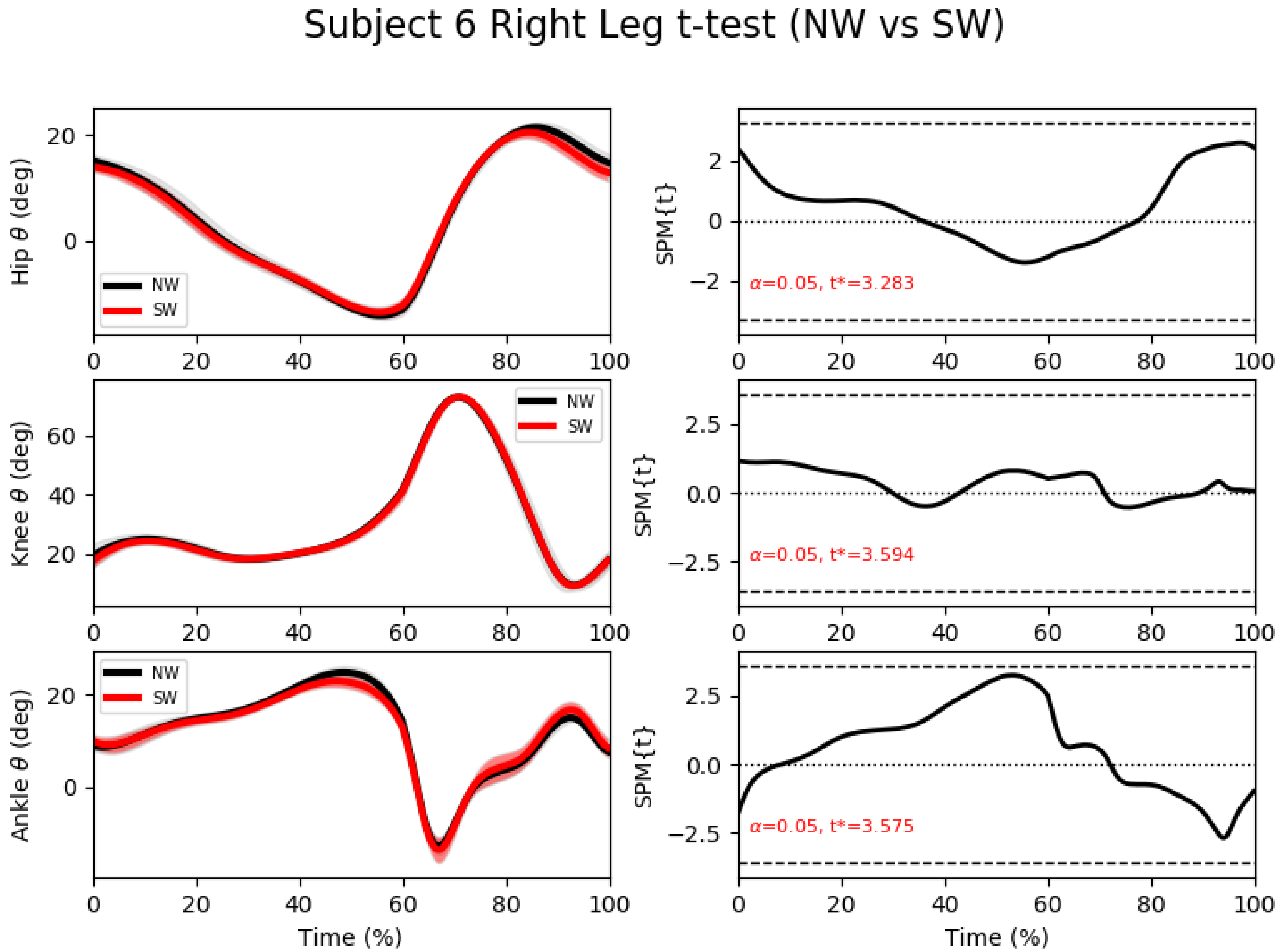

Subject 6 Right Leg t-test (NW vs SW)
Hip θ (deg)
Knee θ (deg)
Ankle θ (deg)
NW
SW
SPM{t}
α=0.05, t*=3.283
α=0.05, t*=3.594
α=0.05, t*=3.575
Time (%)

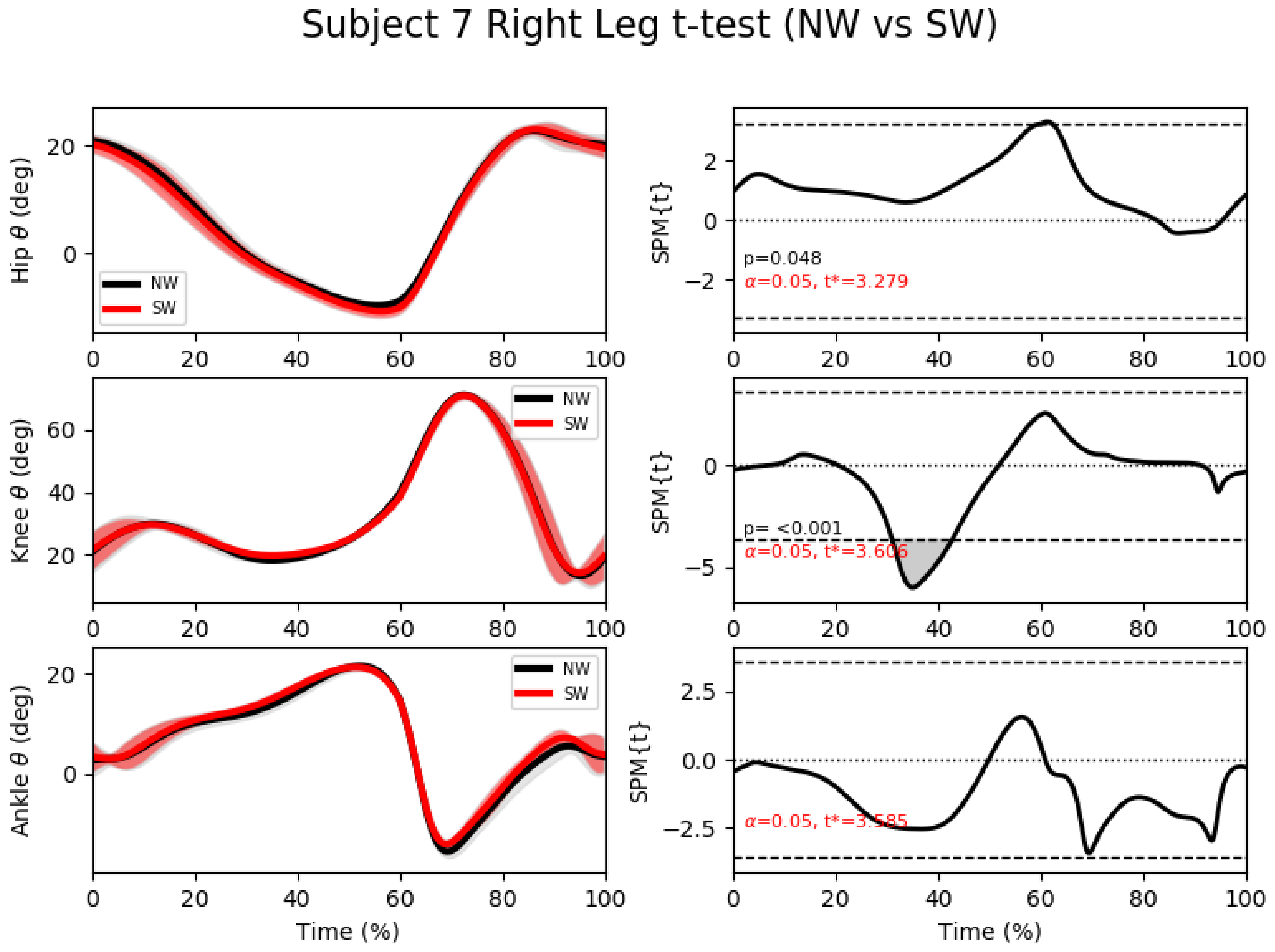

Subject 7 Right Leg t-test (NW vs SW)
Hip θ (deg)
Knee θ (deg)
Ankle θ (deg)
NW
SW
SPM{t}
p=0.048
α=0.05, t*=3.279
p= <0.001
α=0.05, t*=3.606
α=0.05, t*=3.585
Time (%)

TerminalSwing Trip (Left leg)

Subject 1 to 7

Comparing normal walking (NW) to strap walking (SW) to terminal swing trips (TS)

Subject 1 Left Leg ANOVA (NW vs SW vs TS)

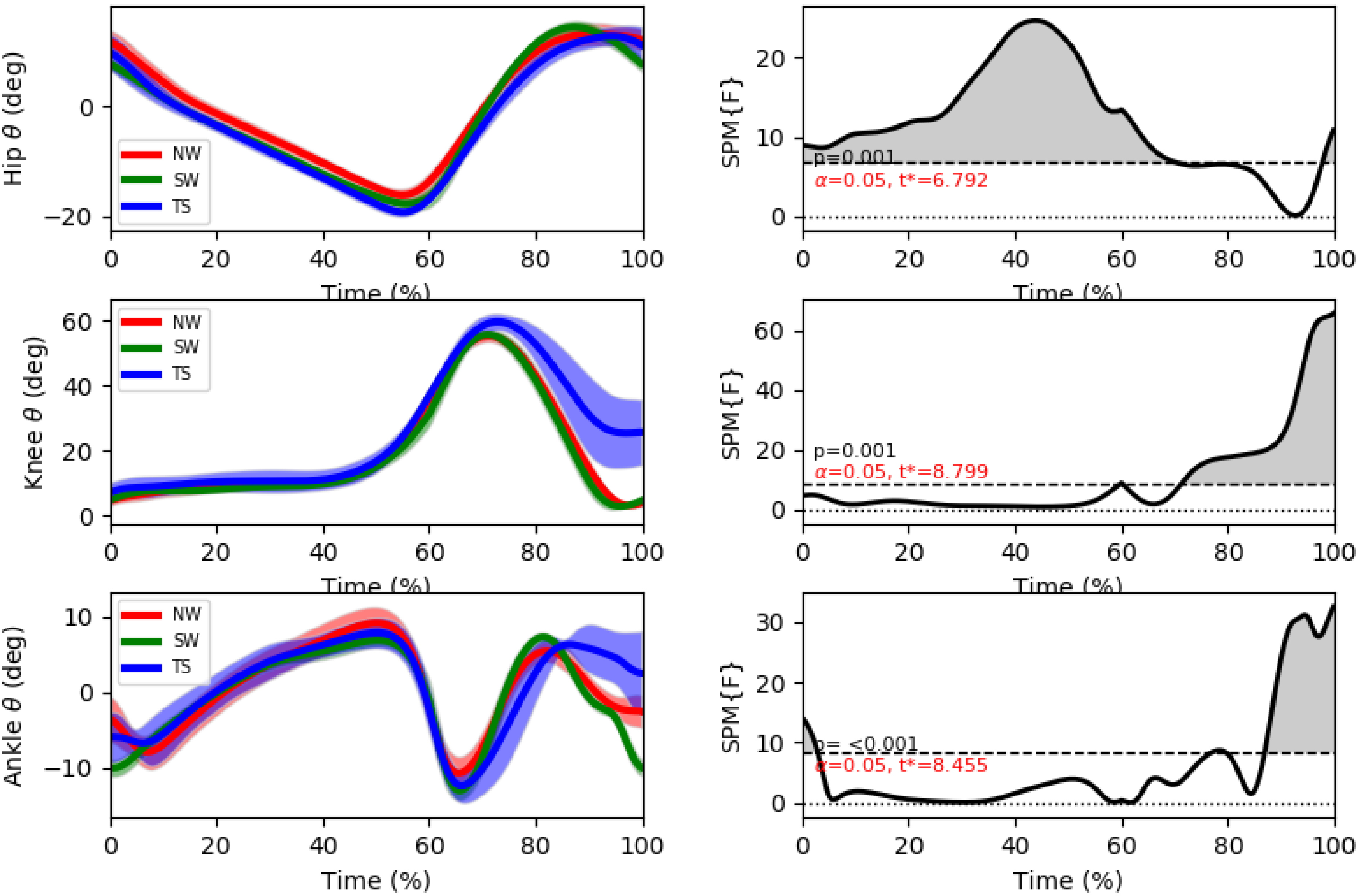

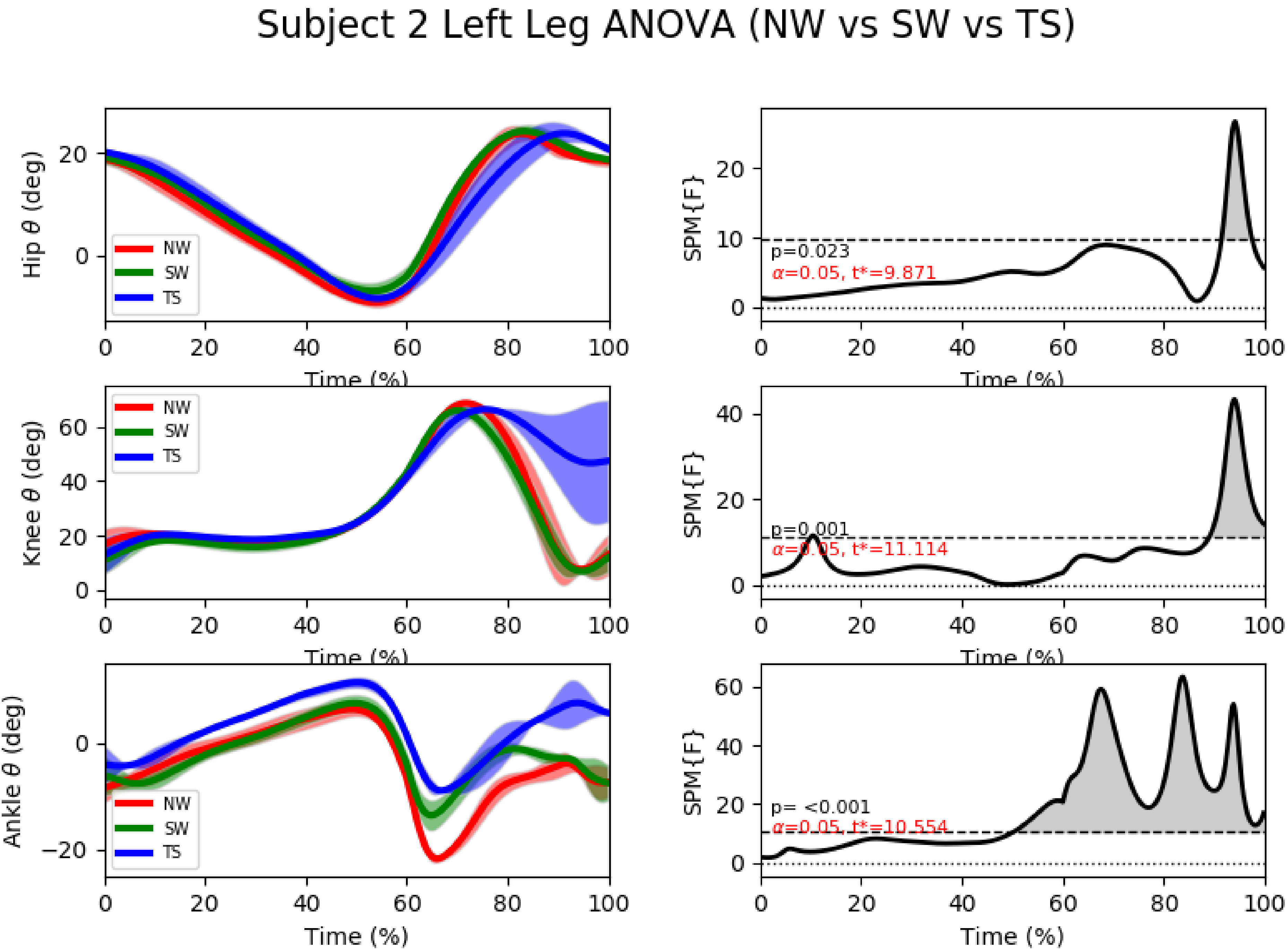

Subject 2 Left Leg ANOVA (NW vs SW vs TS)
Hip θ (deg)
Knee θ (deg)
Ankle θ (deg)
NW
SW
TS
Time (%)
SPM{F}
p=0.023
α=0.05, t*=9.871
p=0.001
α=0.05, t*=11.114
p= <0.001
α=0.05, t*=10.554

Subject 3 Left Leg ANOVA (NW vs SW vs TS)

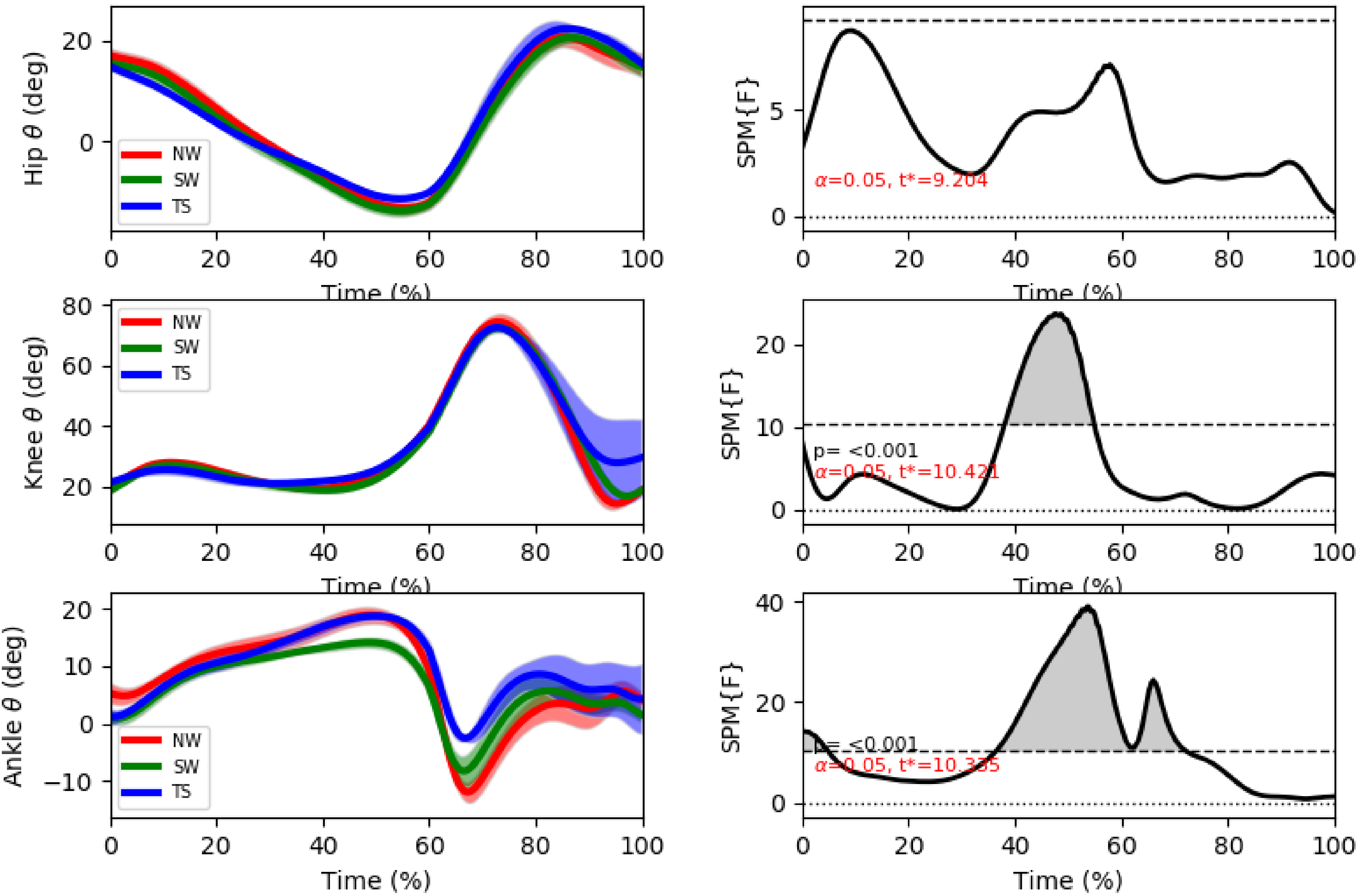

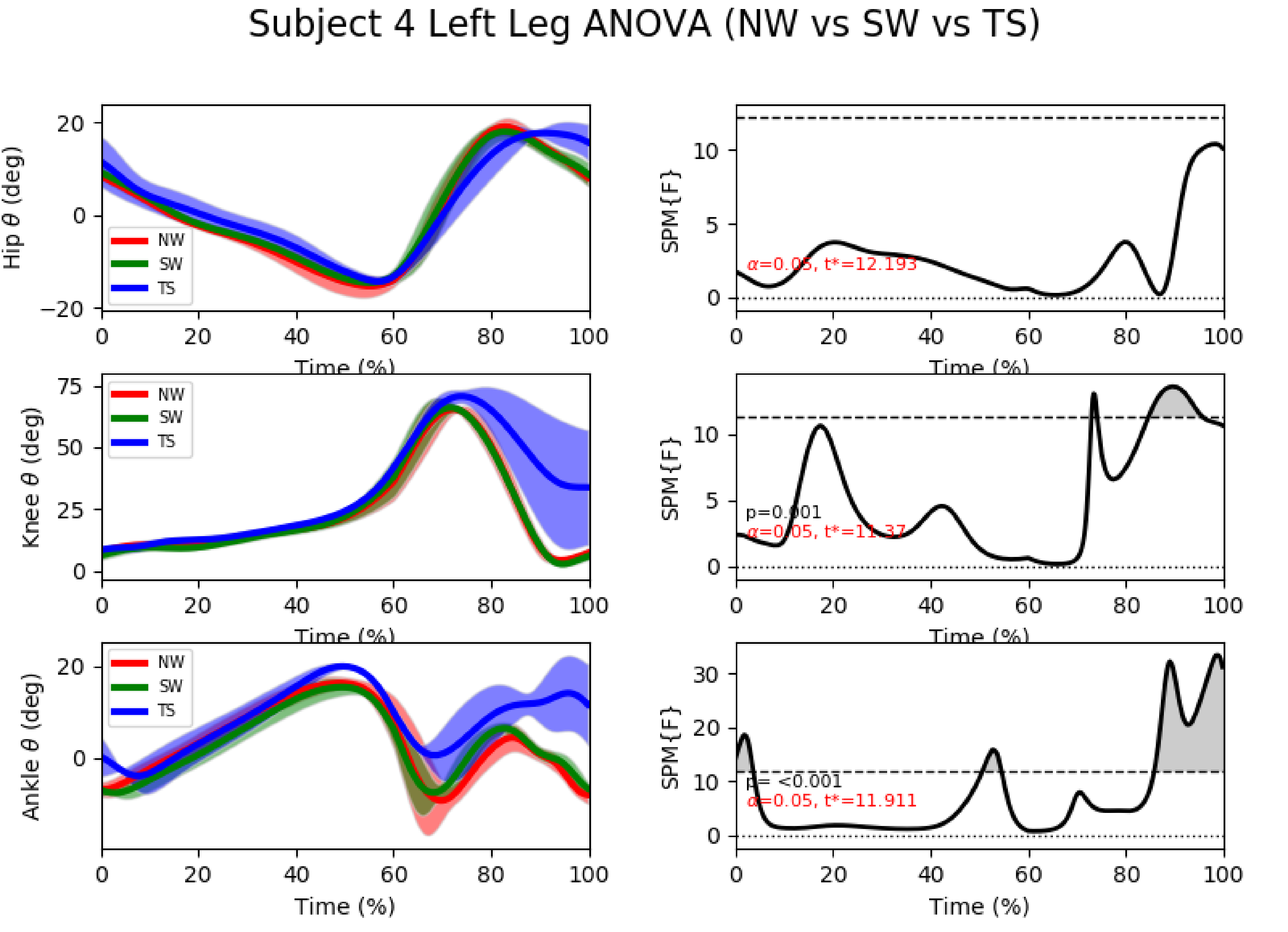
Subject 4 Left Leg ANOVA (NW vs SW vs TS)
Hip θ (deg)
Knee θ (deg)
Ankle θ (deg)
SPM{F}
Time (%)
NW
SW
TS
α=0.05, t*=12.193
p=0.001
α=0.05, t*=11.37
p= <0.001
α=0.05, t*=11.911

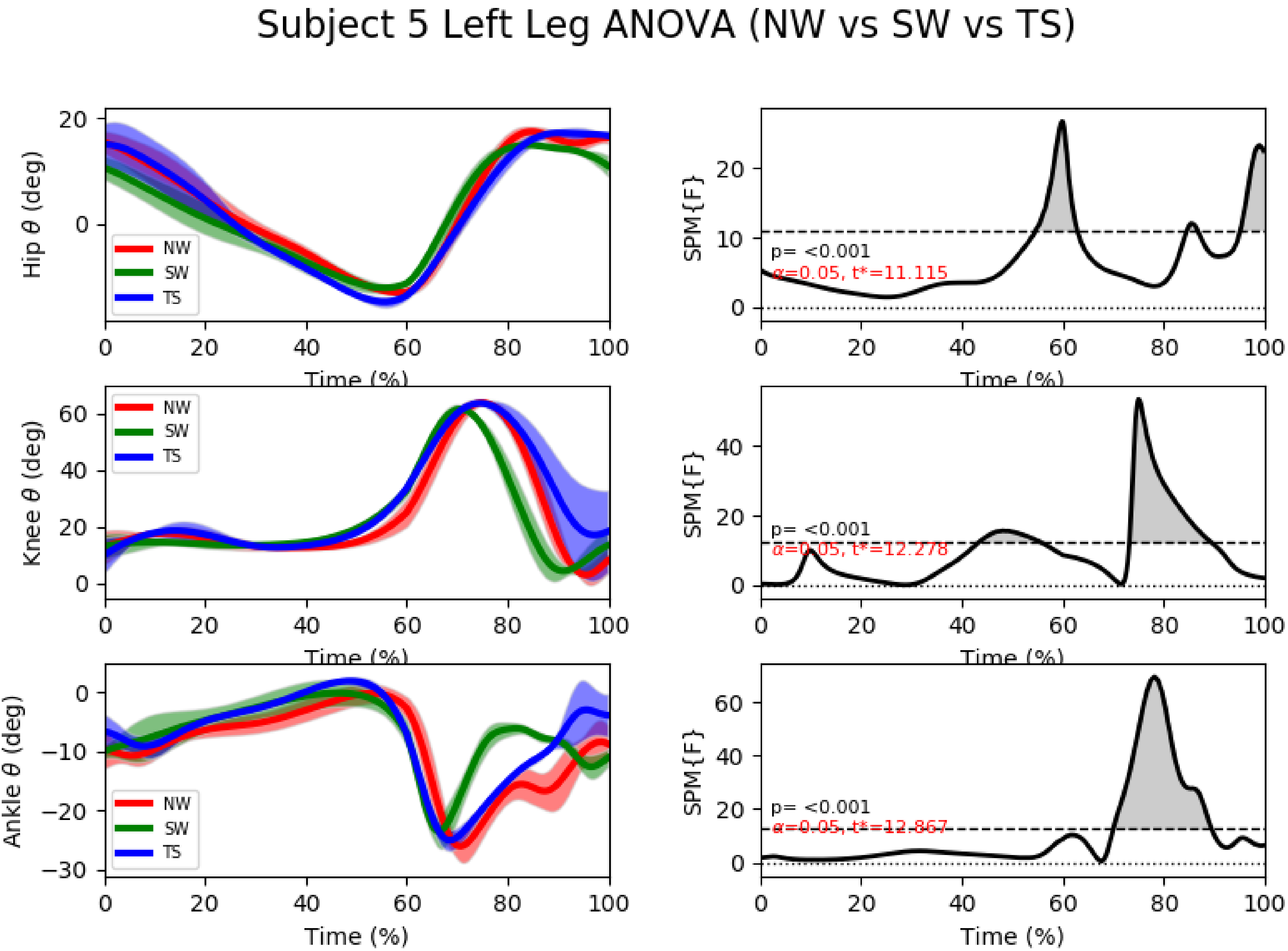

Subject 5 Left Leg ANOVA (NW vs SW vs TS)
Hip θ (deg)
Knee θ (deg)
Ankle θ (deg)
Time (%)
NW
SW
TS
SPM{F}
p= <0.001
α=0.05, t*=11.115
α=0.05, t*=12.278
α=0.05, t*=12.867

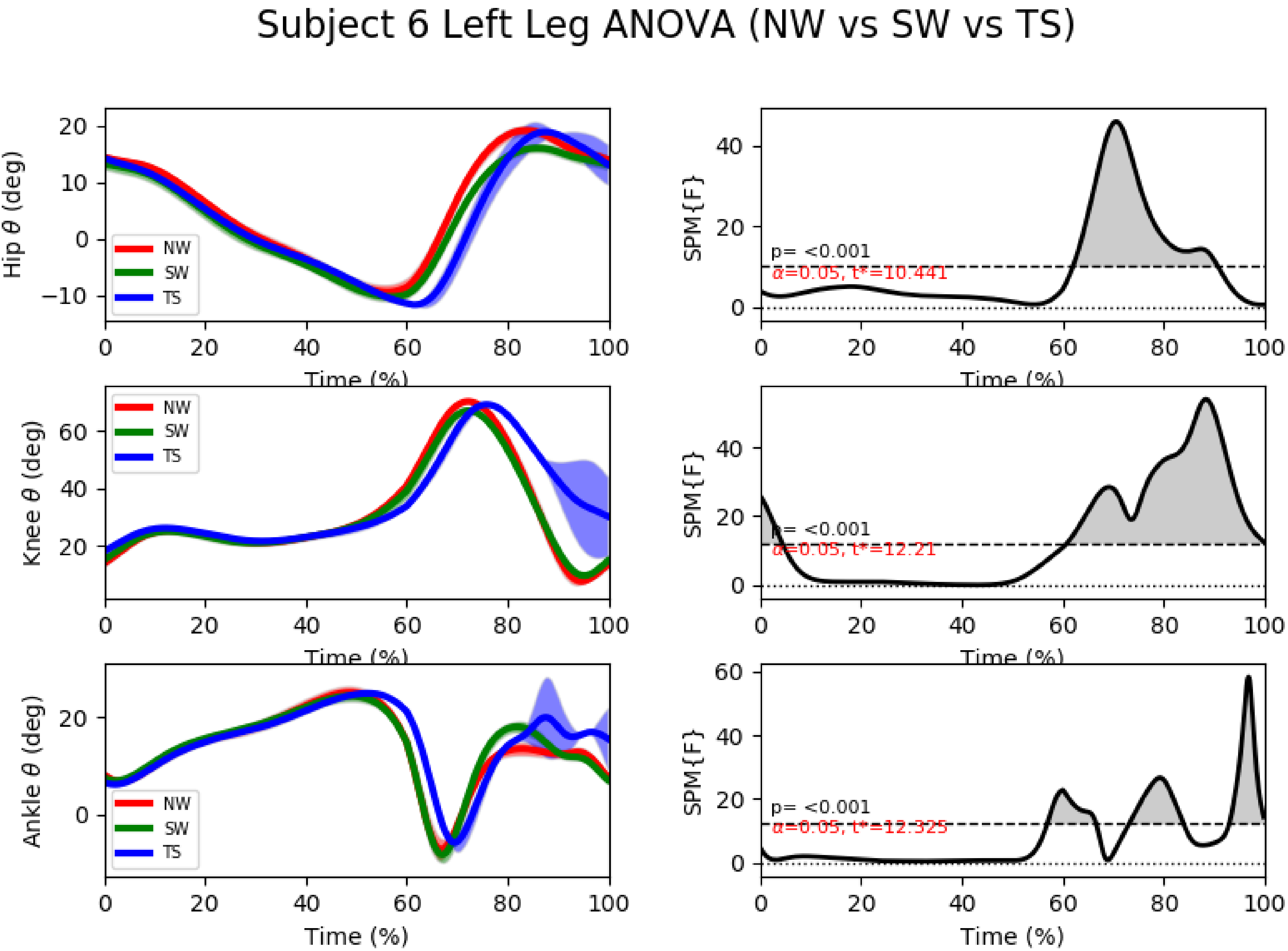
Subject 6 Left Leg ANOVA (NW vs SW vs TS)
Hip θ (deg)
Knee θ (deg)
Ankle θ (deg)
NW
SW
TS
Time (%)
SPM{F}
p= <0.001
α=0.05, t*=10.441
α=0.05, t*=12.21
α=0.05, t*=12.325

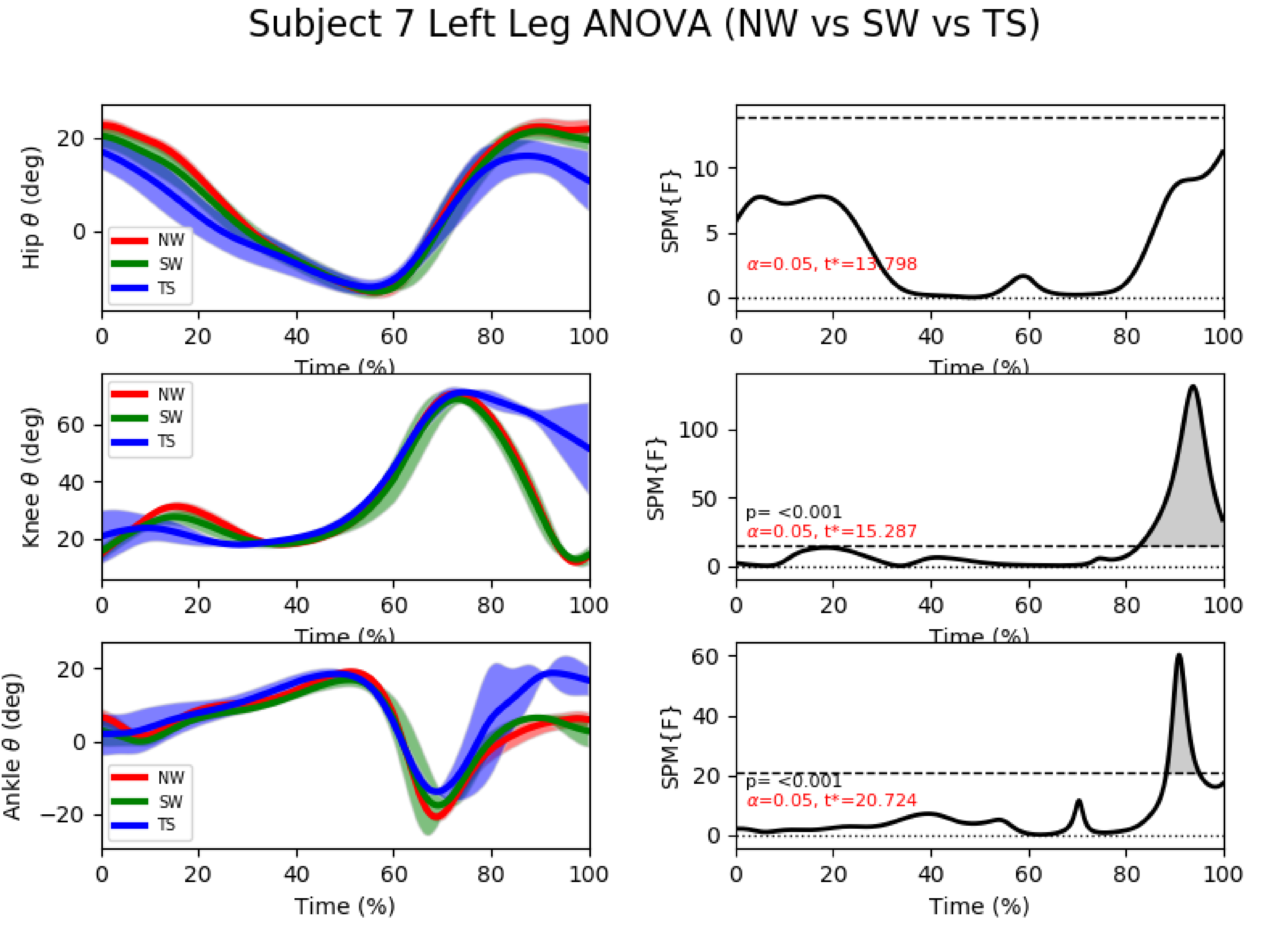

Subject 7 Left Leg ANOVA (NW vs SW vs TS)
Hip θ (deg)
Knee θ (deg)
Ankle θ (deg)
NW
SW
TS
SPM{F}
Time (%)
α=0.05, t*=13.798
p= <0.001
α=0.05, t*=15.287
p= <0.001
α=0.05, t*=20.724

TerminalSwing Trip (Right leg)

Subject 1 to 7

Comparing normal walking (NW) to strap walking (SW) to terminal swing trips (TS)

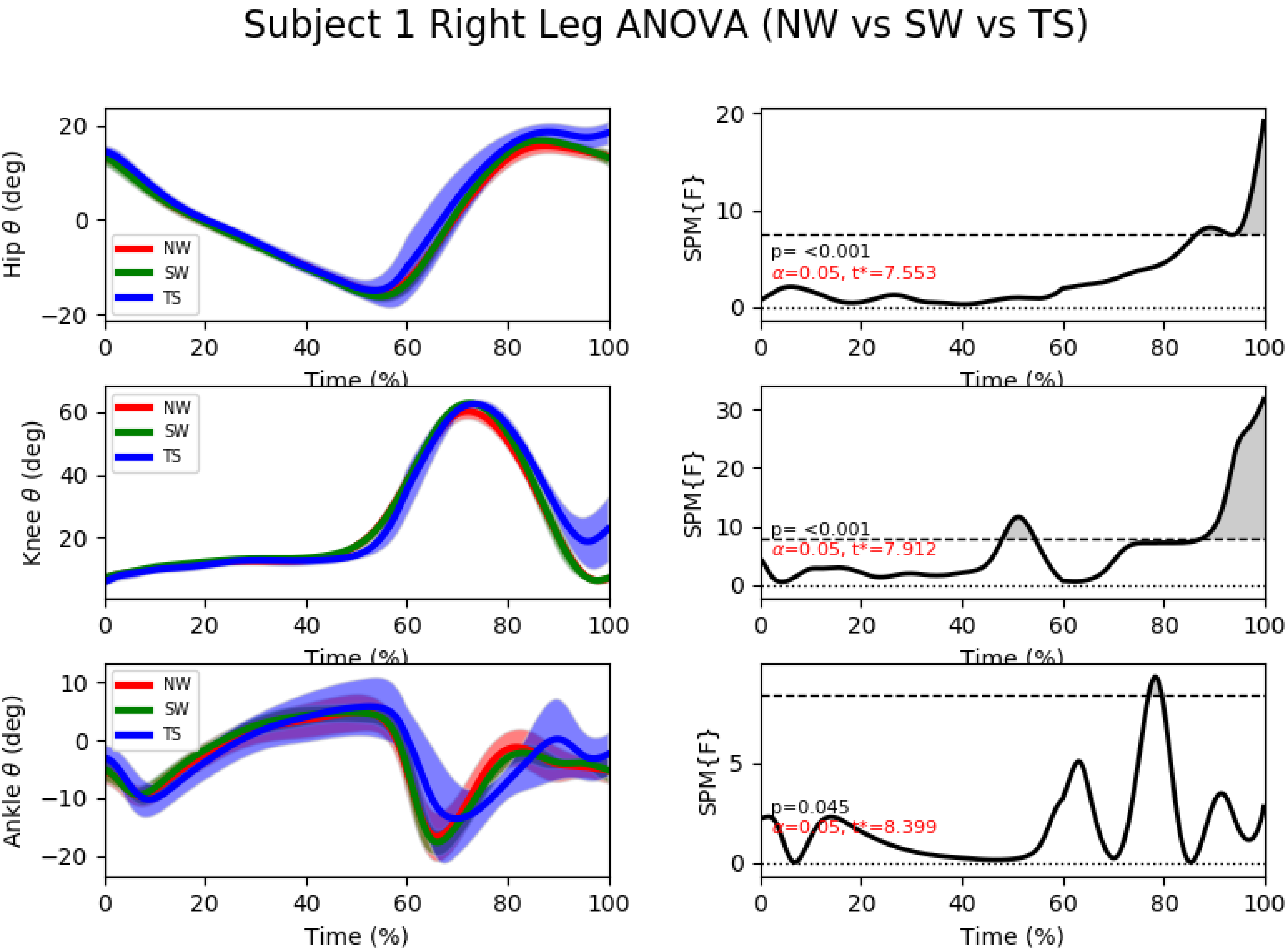

Subject 1 Right Leg ANOVA (NW vs SW vs TS)
Hip θ (deg)
NW
SW
TS
Time (%)
SPM{F}
p= <0.001
α=0.05, t*=7.553
Knee θ (deg)
p= <0.001
α=0.05, t*=7.912
Ankle θ (deg)
p=0.045
α=0.05, t*=8.399

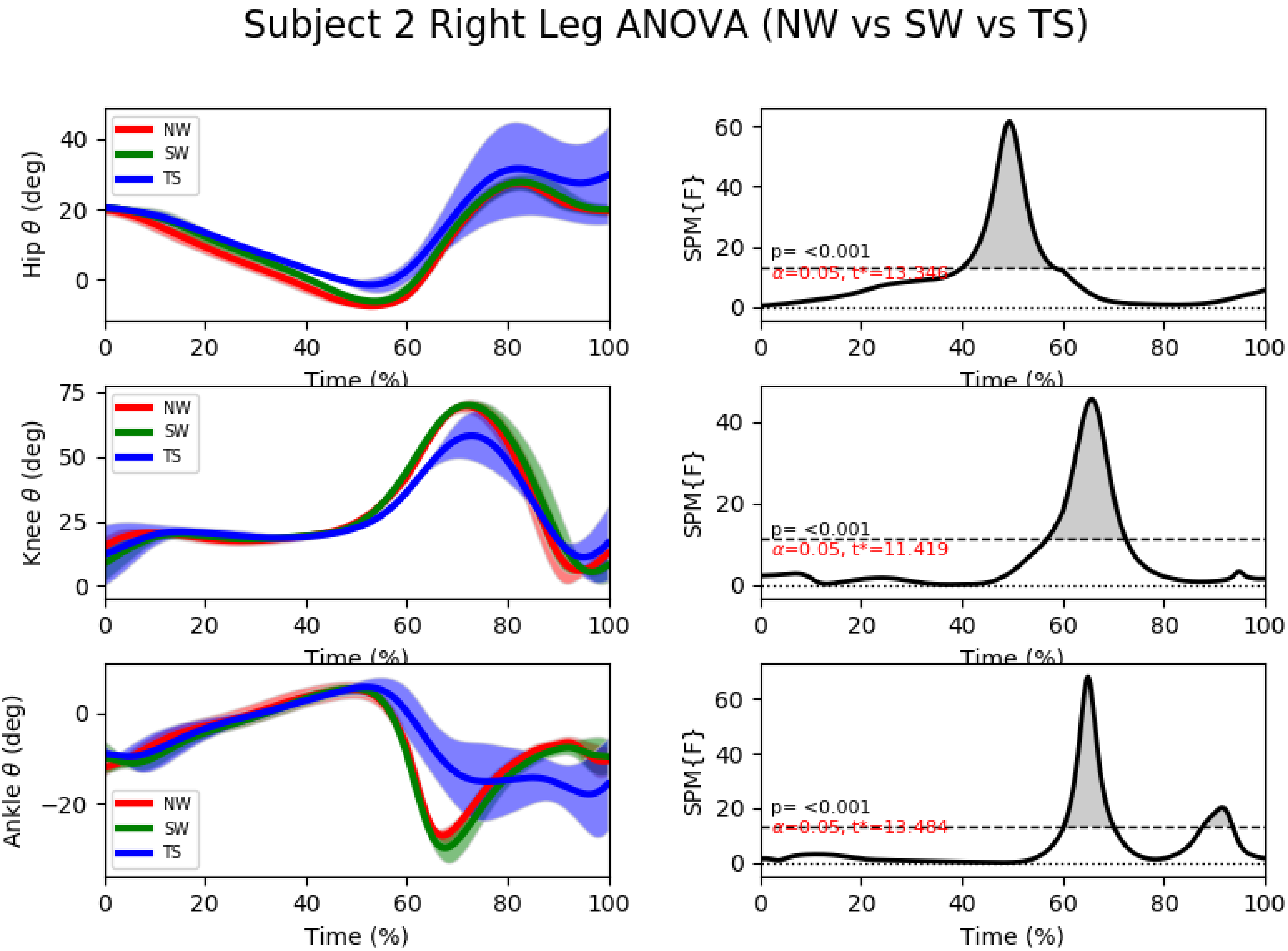
Subject 2 Right Leg ANOVA (NW vs SW vs TS)
NW
SW
TS
Hip θ (deg)
Knee θ (deg)
Ankle θ (deg)
Time (%)
SPM{F}
p= <0.001
α=0.05, t*=13.346
α=0.05, t*=11.419
α=0.05, t*=13.484

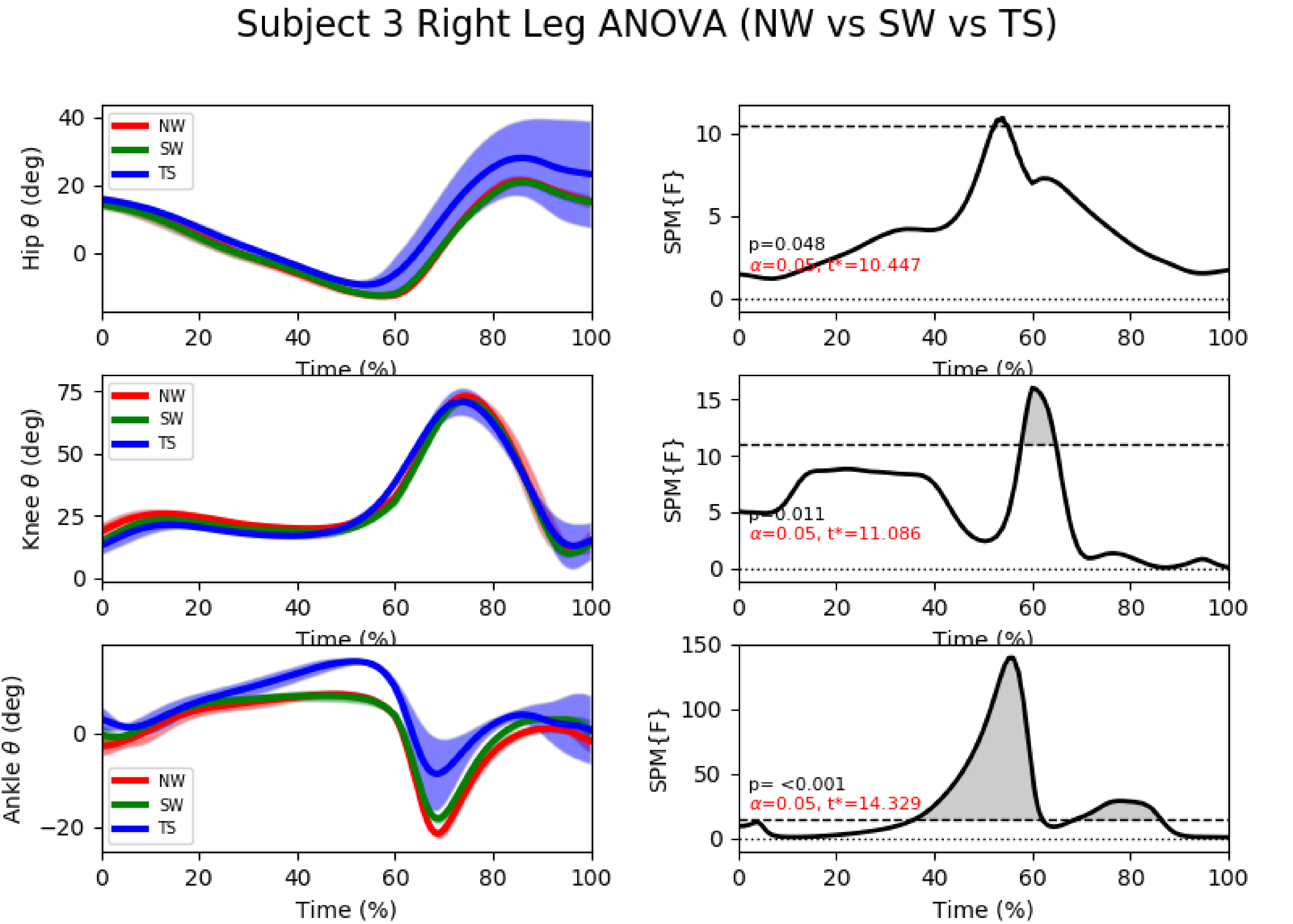
Subject 3 Right Leg ANOVA (NW vs SW vs TS)
NW
SW
TS
Hip θ (deg)
Knee θ (deg)
Ankle θ (deg)
SPM{F}
Time (%)
p=0.048
α=0.05, t*=10.447
p=0.011
α=0.05, t*=11.086
p= <0.001
α=0.05, t*=14.329

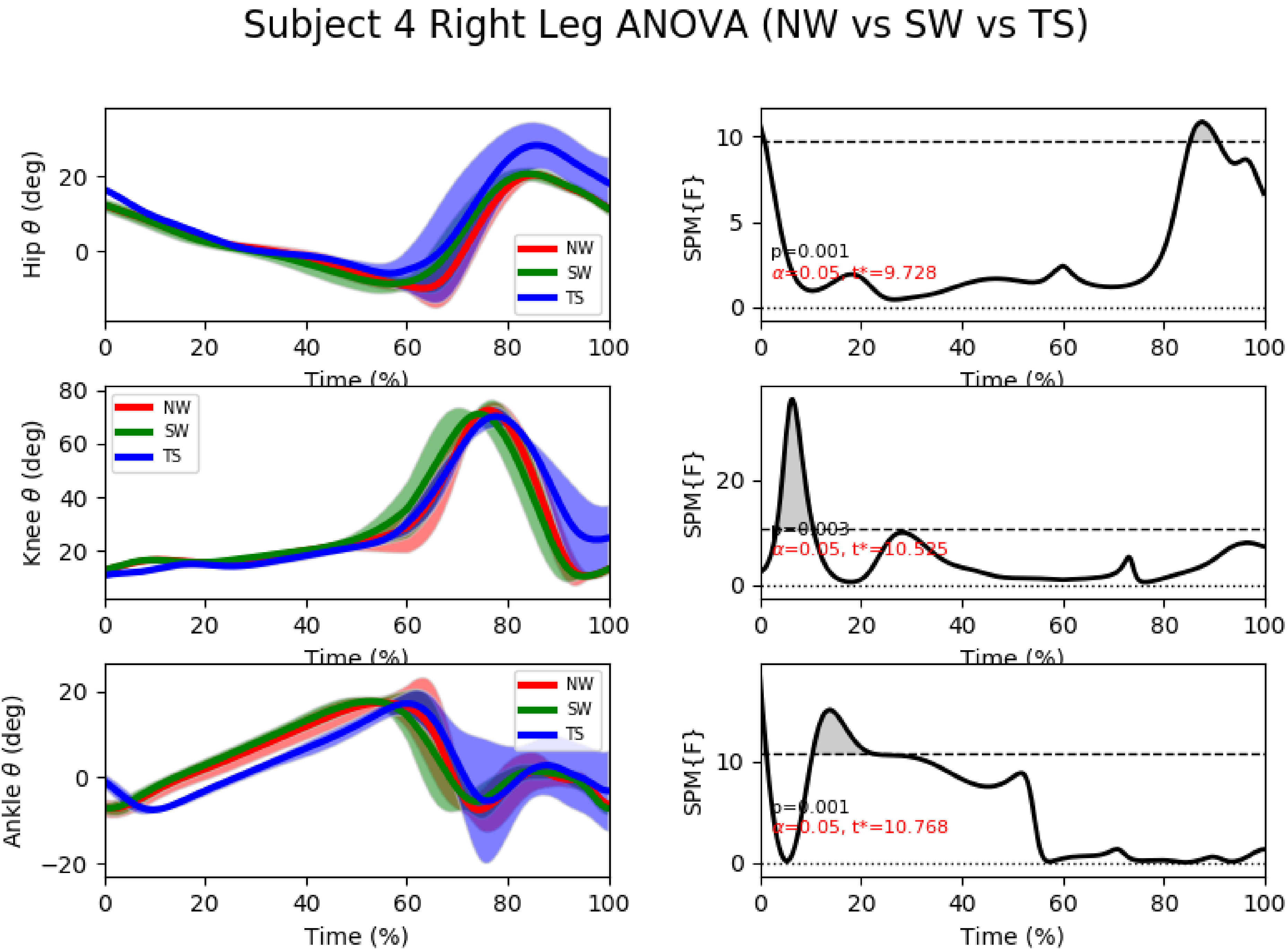
Subject 4 Right Leg ANOVA (NW vs SW vs TS)
Hip θ (deg)
Knee θ (deg)
Ankle θ (deg)
SPM{F}
Time (%)
NW
SW
TS
p=0.001
α=0.05, t*=9.728
p=0.003
α=0.05, t*=10.525
p=0.001
α=0.05, t*=10.768

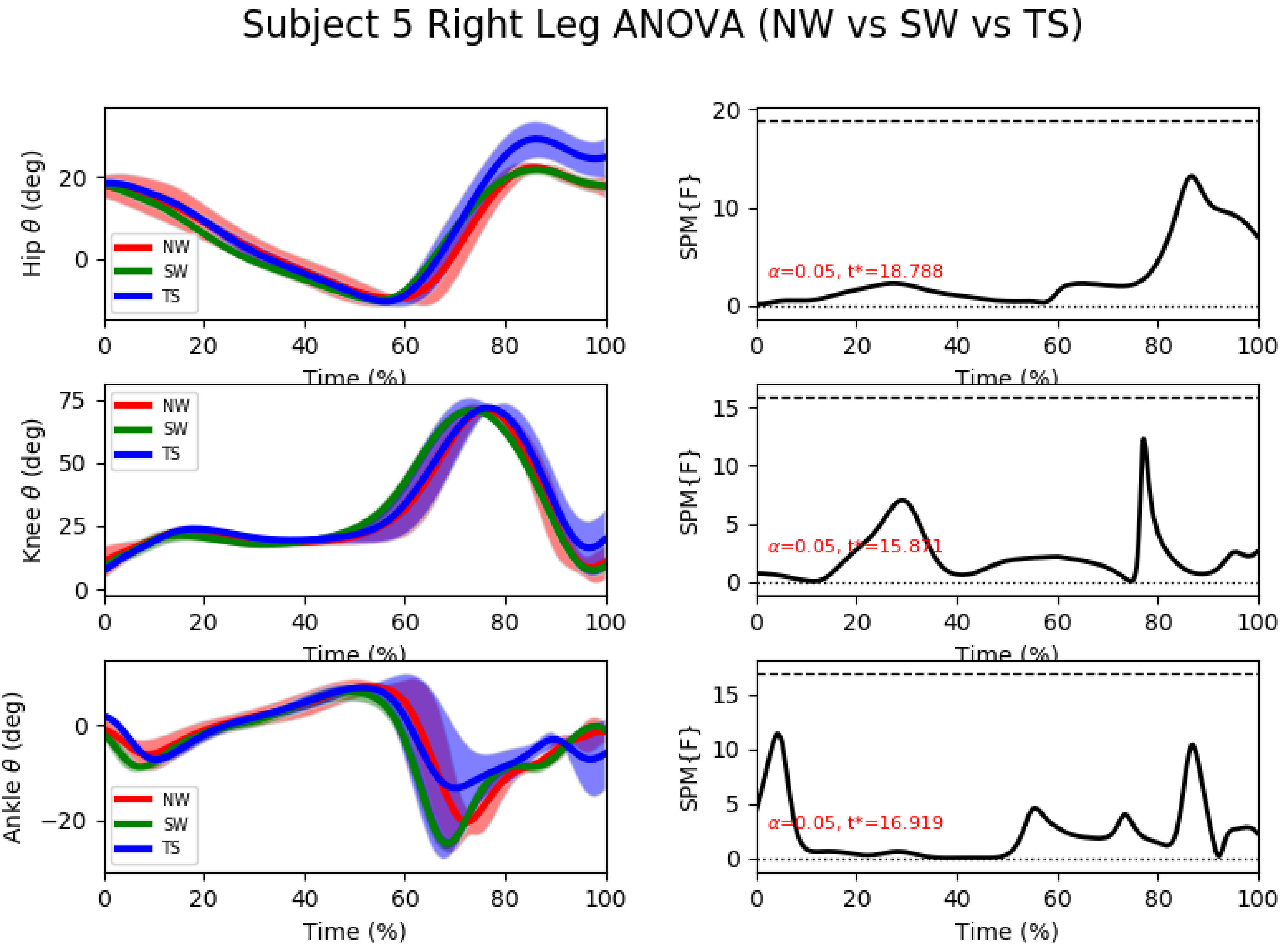
Subject 5 Right Leg ANOVA (NW vs SW vs TS)
Hip θ (deg)
Knee θ (deg)
Ankle θ (deg)
NW
SW
TS
Time (%)
SPM{F}
α=0.05, t*=18.788
α=0.05, t*=15.871
α=0.05, t*=16.919

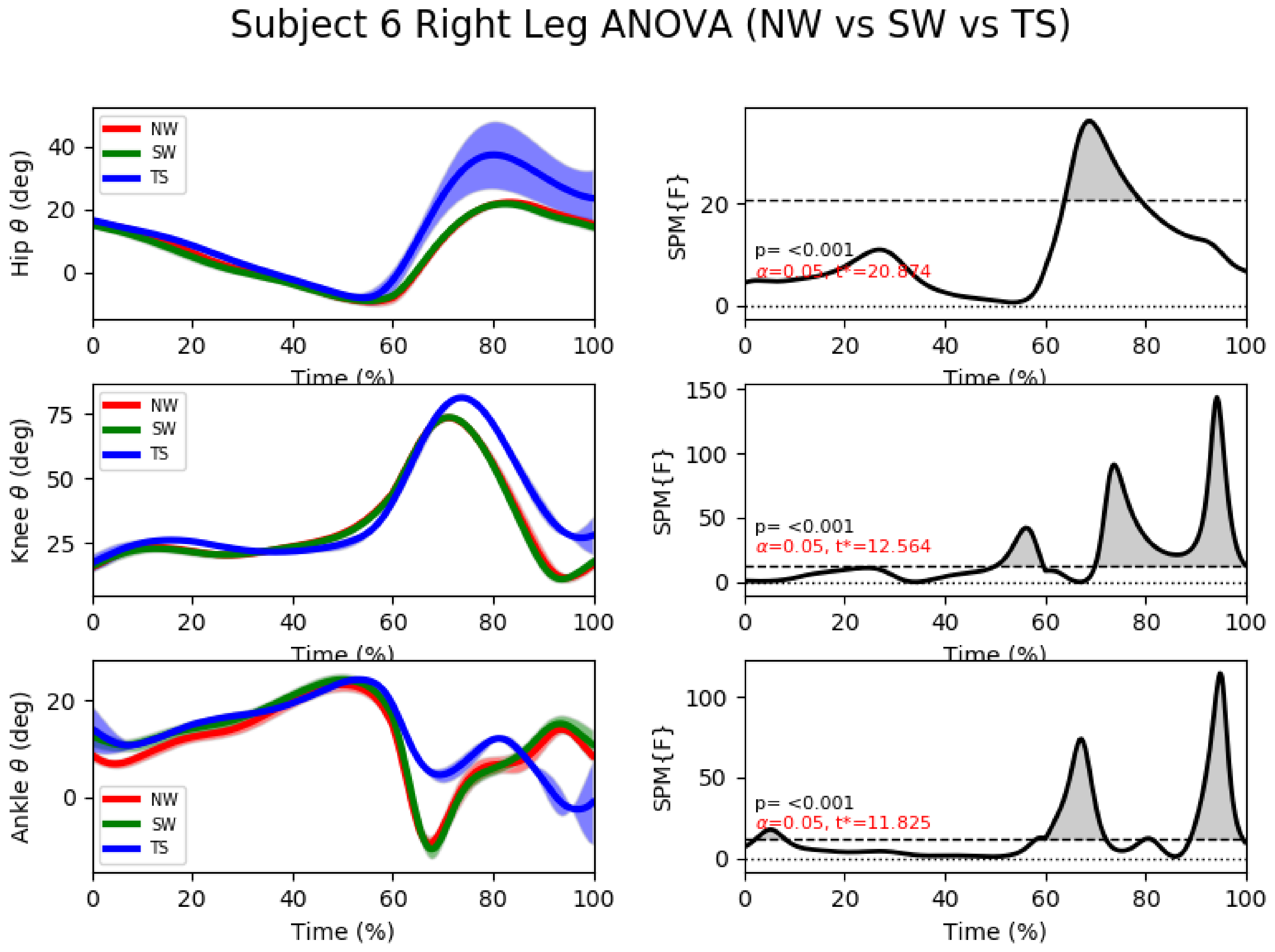

Subject 6 Right Leg ANOVA (NW vs SW vs TS)
NW
SW
TS
Hip θ (deg)
Knee θ (deg)
Ankle θ (deg)
Time (%)
SPM{F}
p= <0.001
α=0.05, t*=20.874
α=0.05, t*=12.564
α=0.05, t*=11.825

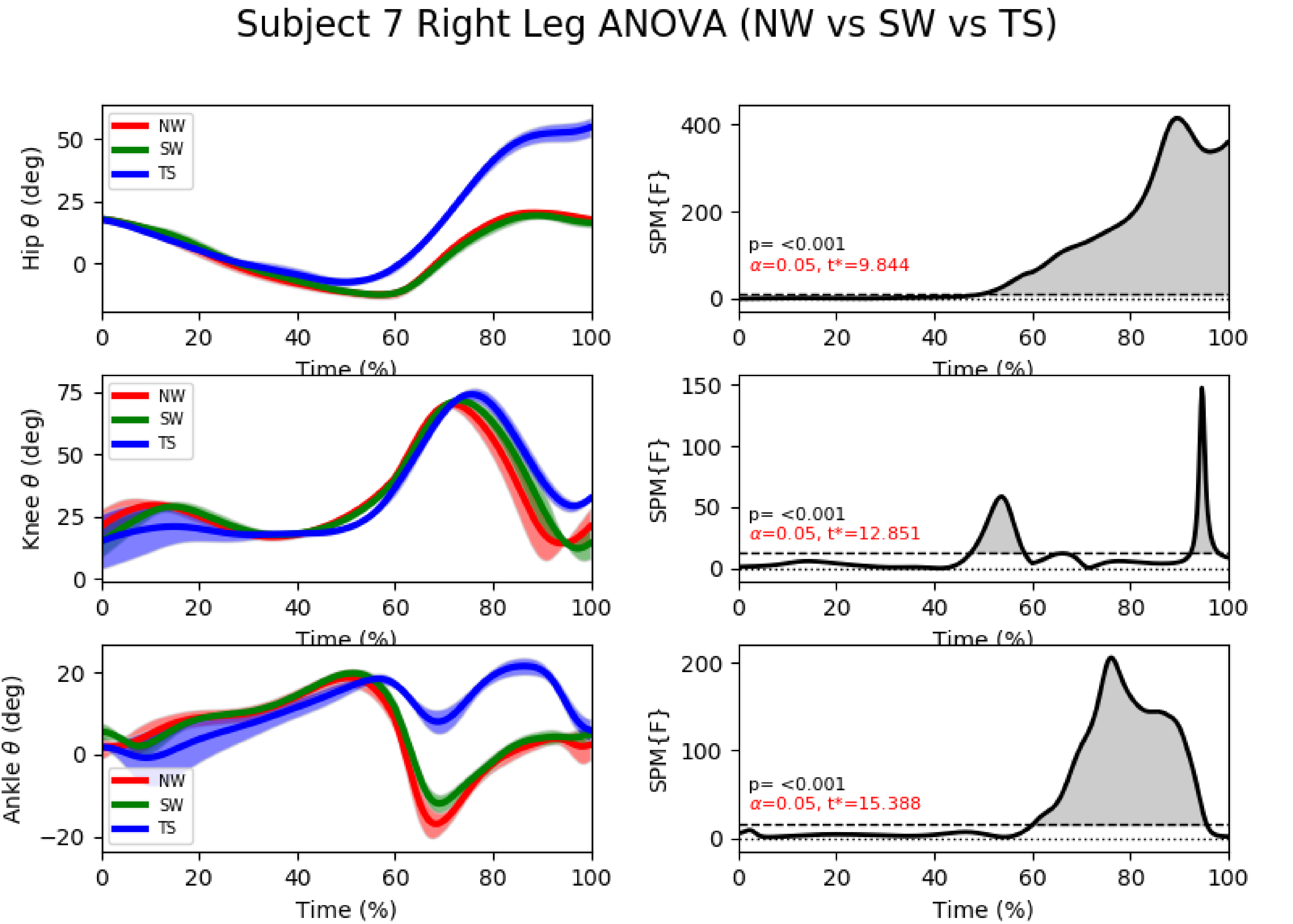
Subject 7 Right Leg ANOVA (NW vs SW vs TS)
NW
SW
TS
Hip θ (deg)
Knee θ (deg)
Ankle θ (deg)
Time (%)
SPM{F}
p= <0.001
α=0.05, t*=9.844
α=0.05, t*=12.851
α=0.05, t*=15.388

MidSwing Trip (Left leg)

Subject 1 to 7

Comparing normal walking (NW) to strap walking (SW) to mid swing trips (MS)

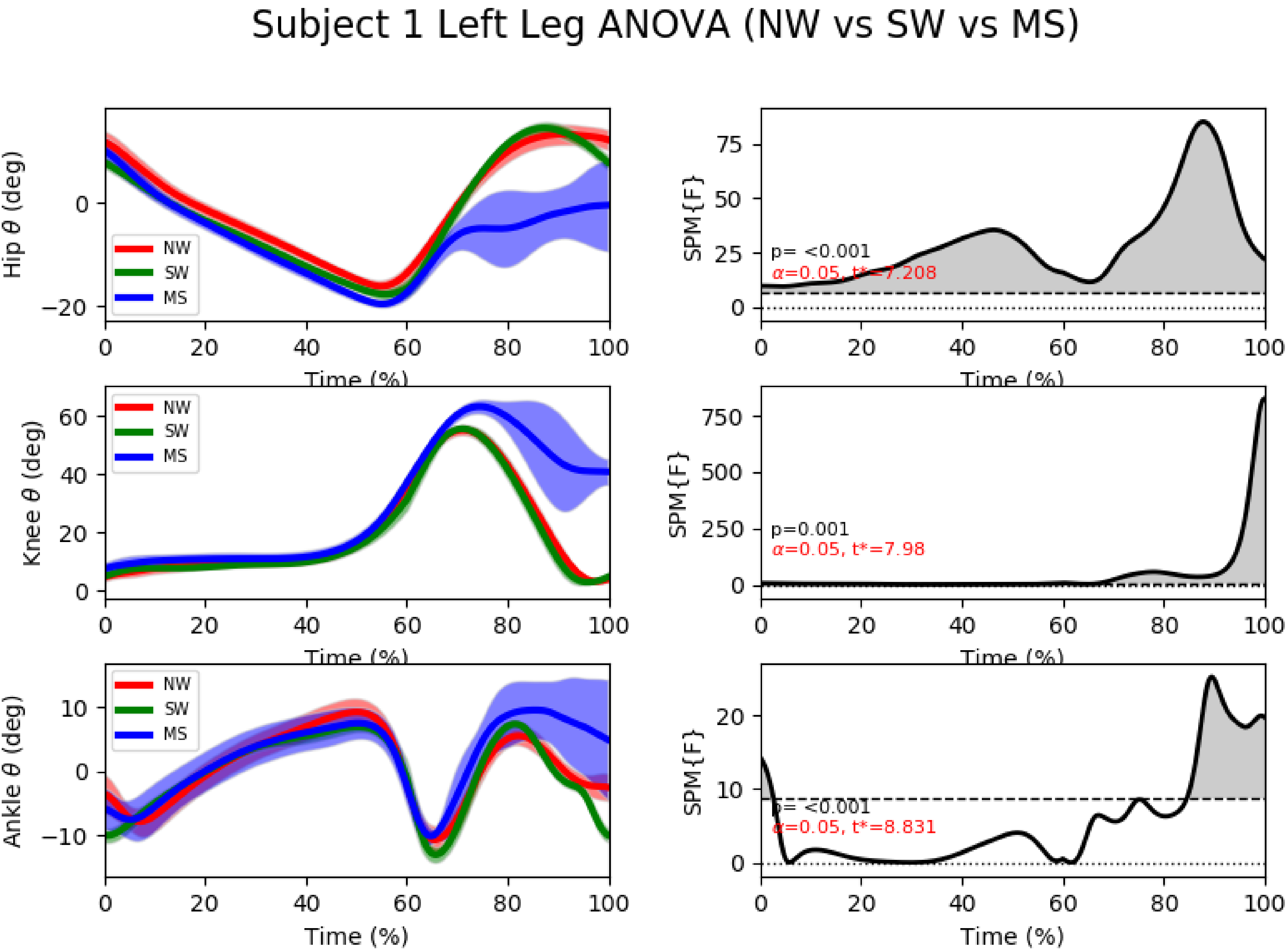
Subject 1 Left Leg ANOVA (NW vs SW vs MS)
Hip θ (deg)
Knee θ (deg)
Ankle θ (deg)
NW
SW
MS
SPM{F}
Time (%)
p= <0.001
α=0.05, t*=7.208
p=0.001
α=0.05, t*=7.98
p= <0.001
α=0.05, t*=8.831

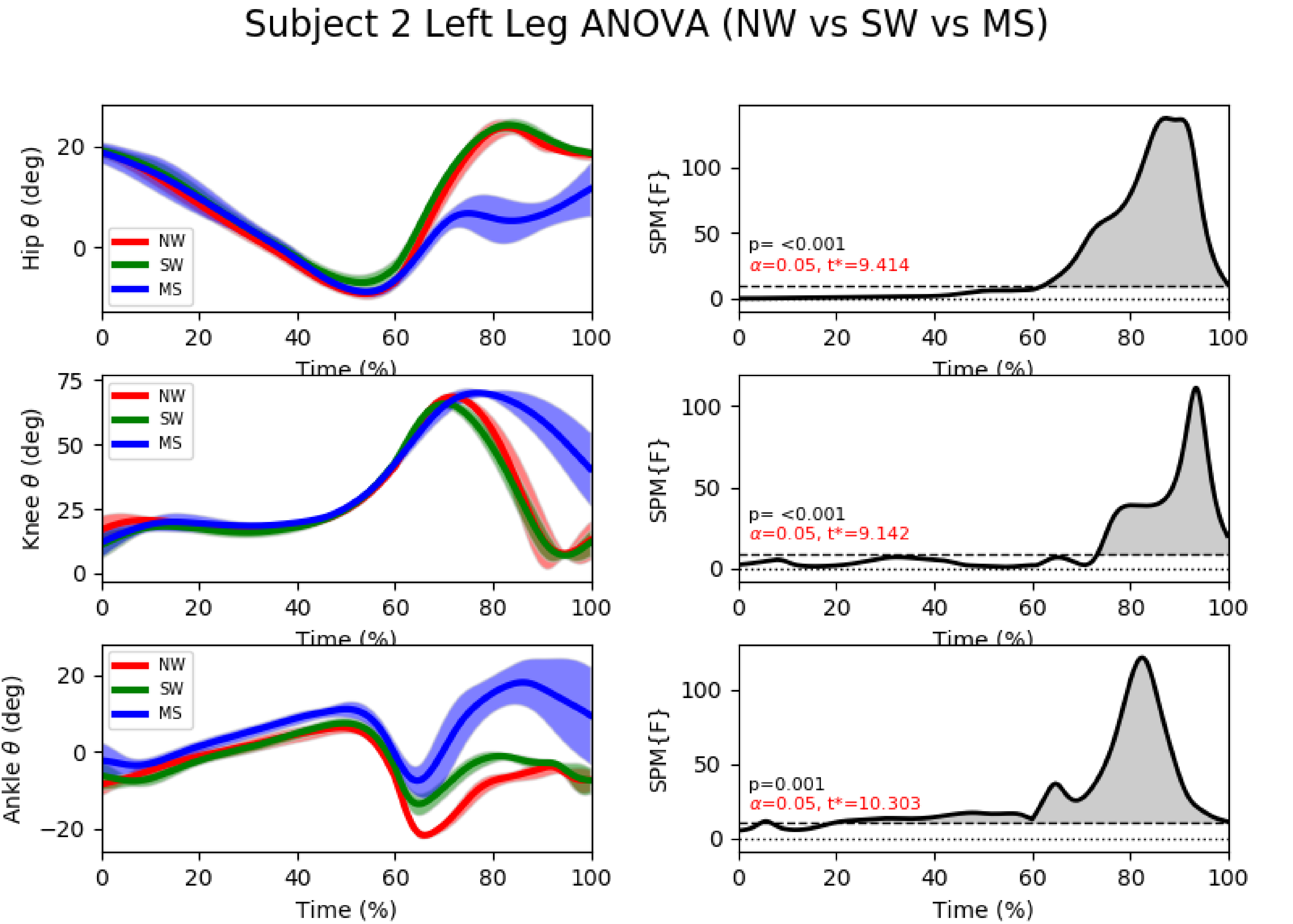

Subject 2 Left Leg ANOVA (NW vs SW vs MS)
Hip θ (deg)
Knee θ (deg)
Ankle θ (deg)
NW
SW
MS
Time (%)
SPM{F}
p= <0.001
α=0.05, t*=9.414
p= <0.001
α=0.05, t*=9.142
p=0.001
α=0.05, t*=10.303

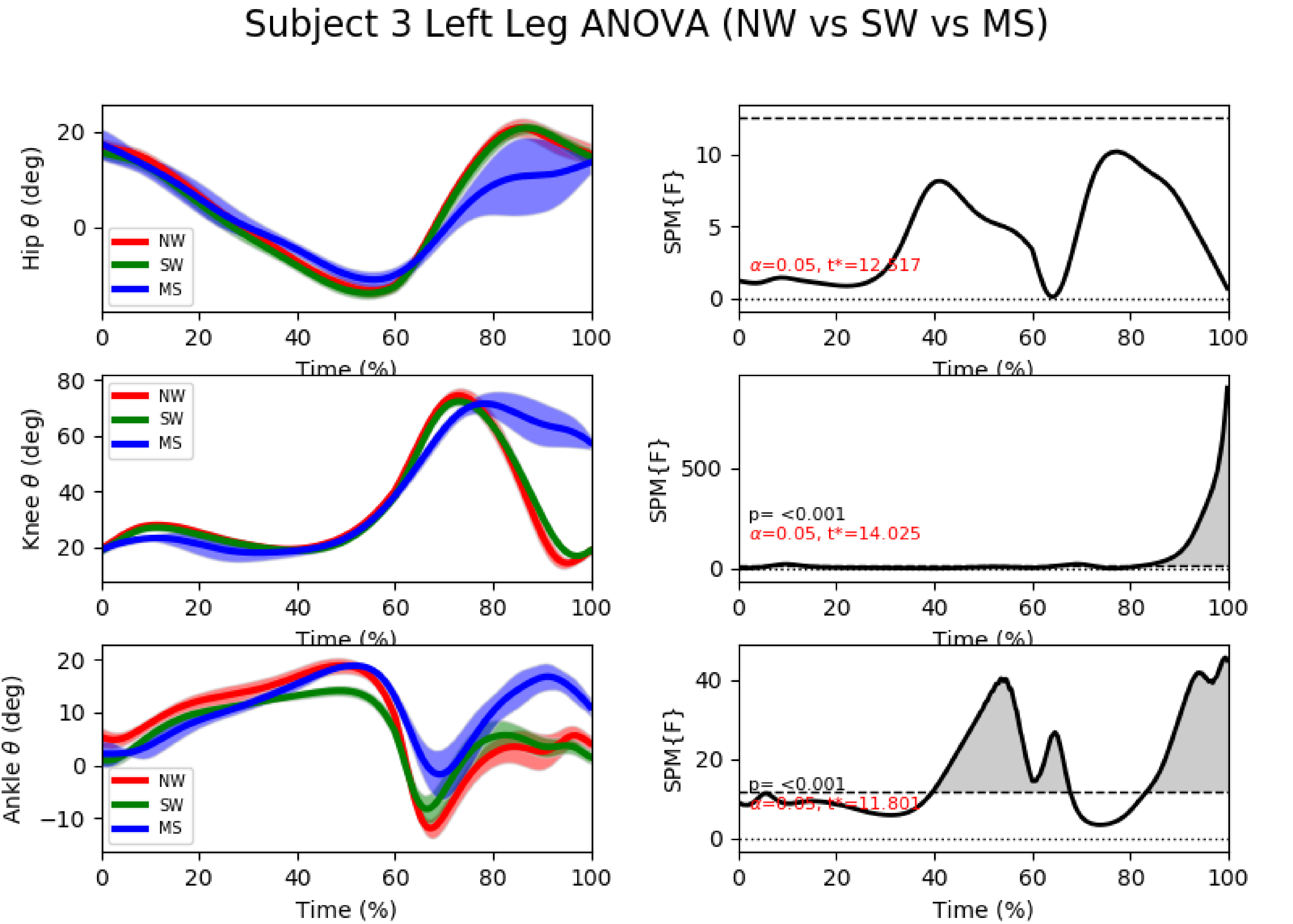

Subject 3 Left Leg ANOVA (NW vs SW vs MS)
Hip θ (deg)
Knee θ (deg)
Ankle θ (deg)
NW
SW
MS
SPM{F}
Time (%)
α=0.05, t*=12.517
p= <0.001
α=0.05, t*=14.025
p= <0.001
α=0.05, t*=11.801

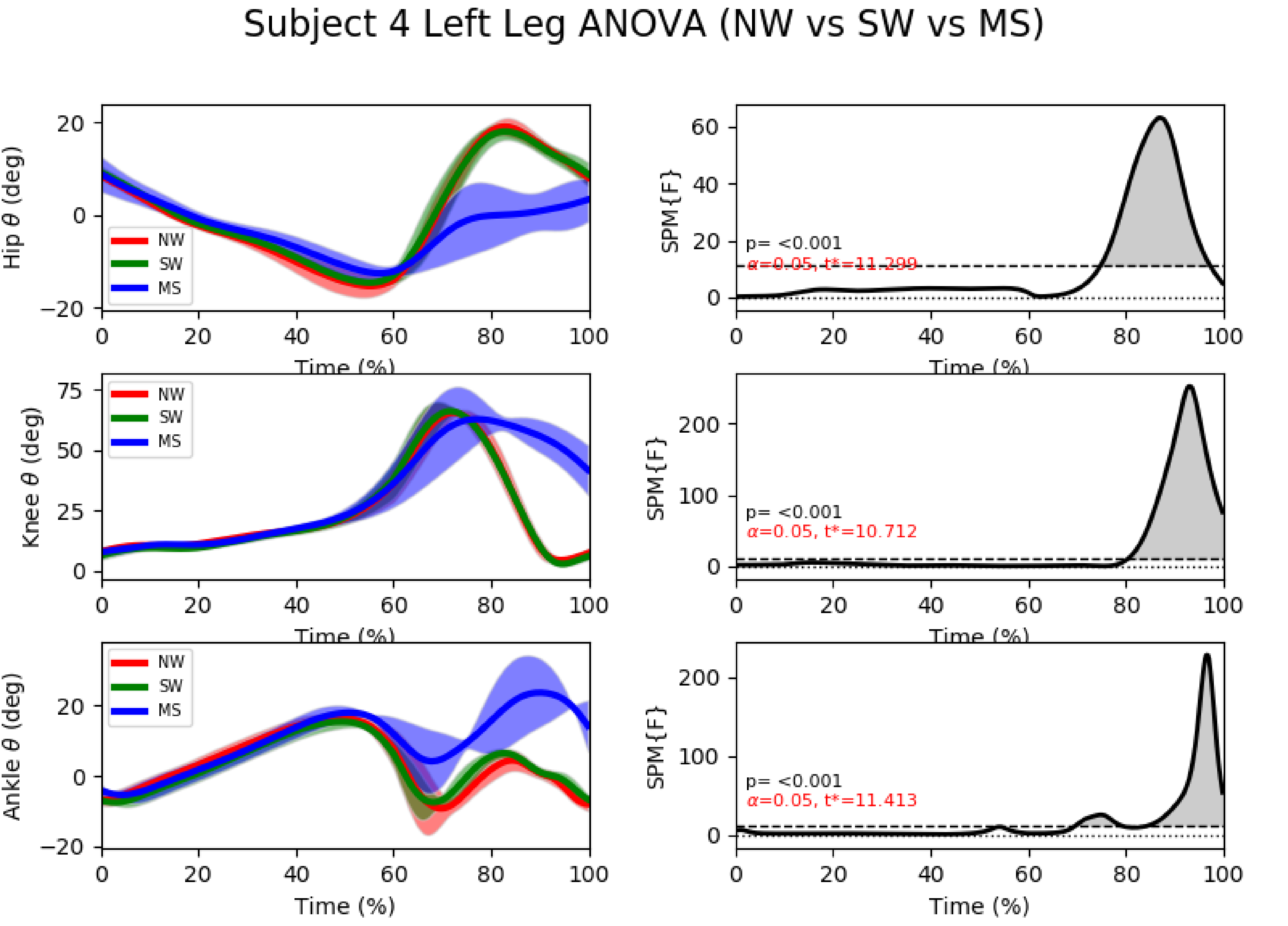
Subject 4 Left Leg ANOVA (NW vs SW vs MS)
Hip θ (deg)
Knee θ (deg)
Ankle θ (deg)
NW
SW
MS
SPM{F}
Time (%)
p= <0.001
α=0.05, t*=11.299
α=0.05, t*=10.712
α=0.05, t*=11.413

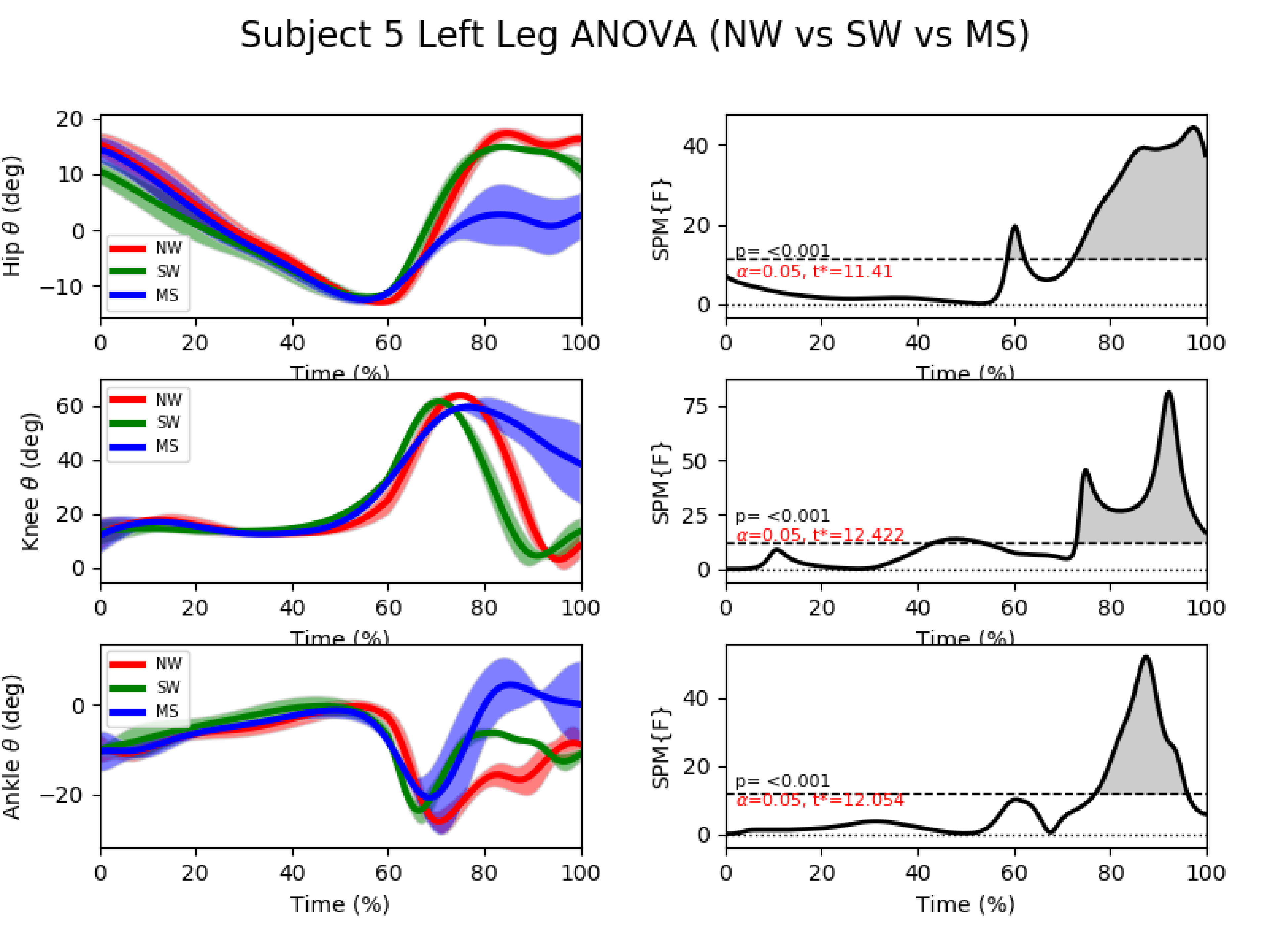

Subject 5 Left Leg ANOVA (NW vs SW vs MS)
Hip θ (deg)
Knee θ (deg)
Ankle θ (deg)
NW
SW
MS
SPM{F}
p= <0.001
α=0.05, t*=11.41
α=0.05, t*=12.422
α=0.05, t*=12.054
Time (%)

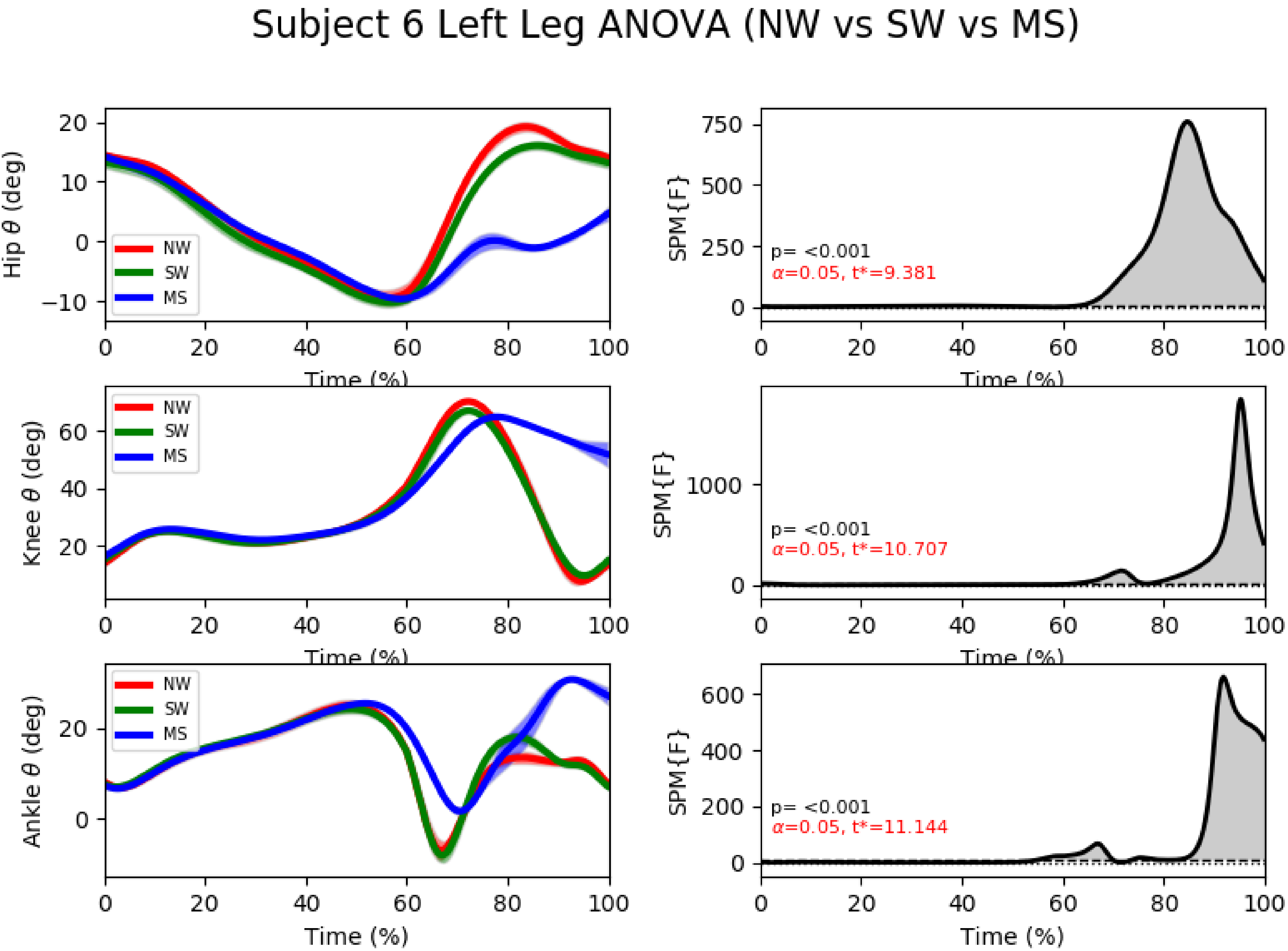

Subject 6 Left Leg ANOVA (NW vs SW vs MS)
Hip θ (deg)
Knee θ (deg)
Ankle θ (deg)
NW
SW
MS
SPM{F}
Time (%)
p= <0.001
α=0.05, t*=9.381
p= <0.001
α=0.05, t*=10.707
p= <0.001
α=0.05, t*=11.144

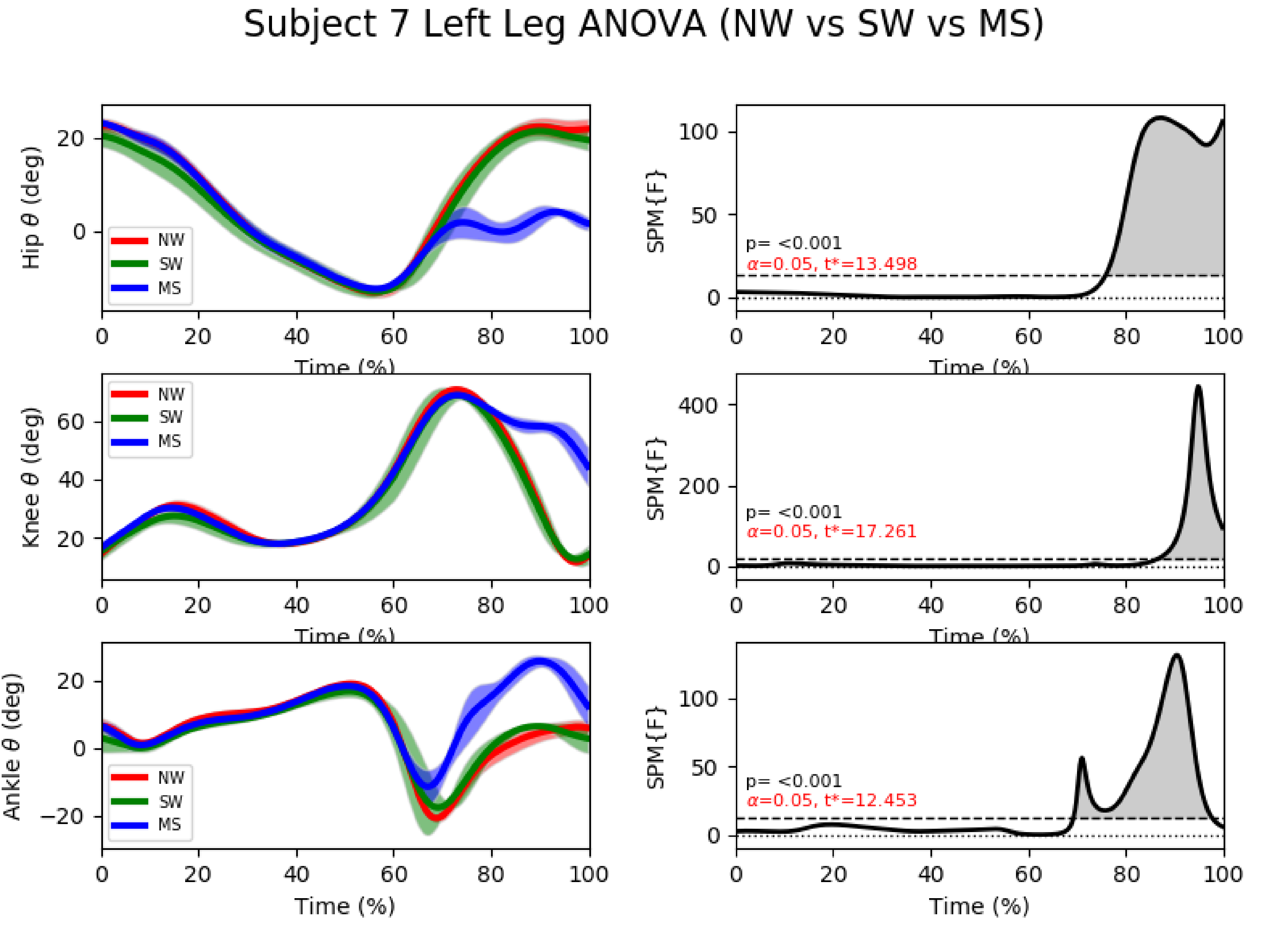

Subject 7 Left Leg ANOVA (NW vs SW vs MS)
Hip θ (deg)
NW
SW
MS
Time (%)
SPM{F}
p= <0.001
α=0.05, t*=13.498
Time (%)
Knee θ (deg)
NW
SW
MS
Time (%)
SPM{F}
p= <0.001
α=0.05, t*=17.261
Time (%)
Ankle θ (deg)
NW
SW
MS
Time (%)
SPM{F}
p= <0.001
α=0.05, t*=12.453
Time (%)

MidSwing Trip (Right leg)

Subject 1 to 7

Comparing normal walking (NW) to strap walking (SW) to mid swing trips (MS)

Subject 1 Right Leg ANOVA (NW vs SW vs MS)

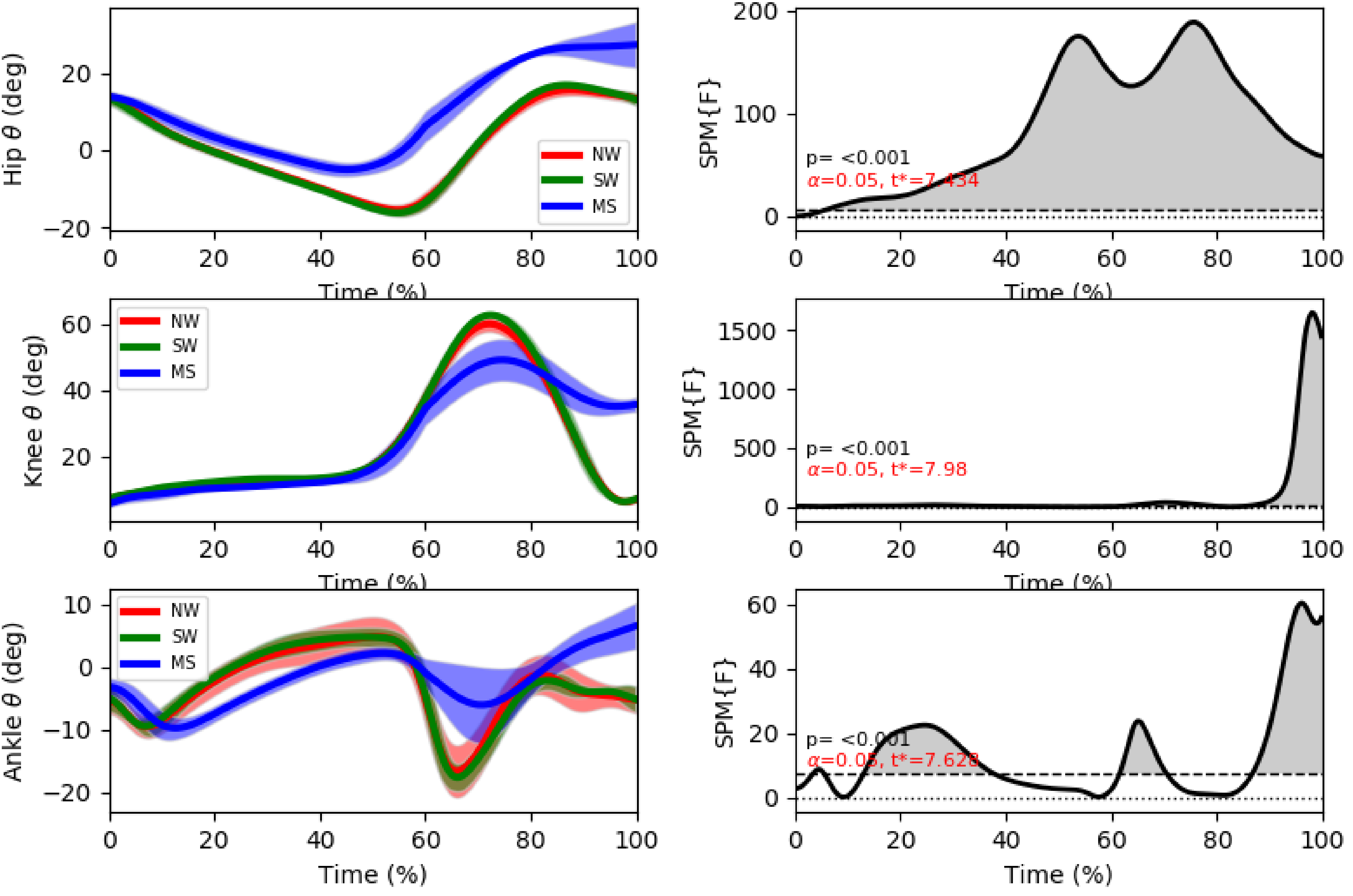

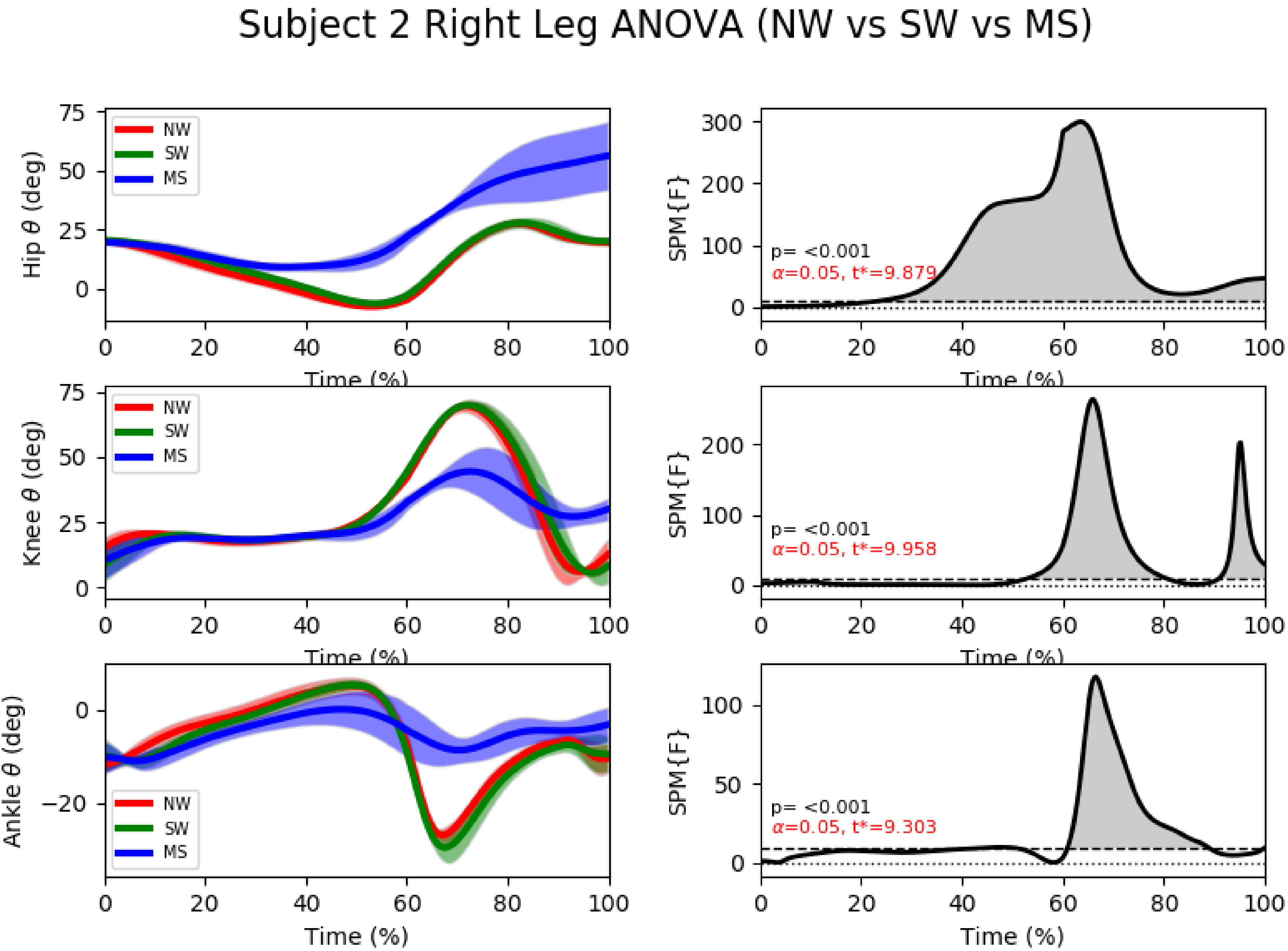
Subject 2 Right Leg ANOVA (NW vs SW vs MS)
NW
SW
MS
Hip θ (deg)
Knee θ (deg)
Ankle θ (deg)
Time (%)
SPM{F}
p= <0.001
α=0.05, t*=9.879
α=0.05, t*=9.958
α=0.05, t*=9.303

Subject 3 Right Leg ANOVA (NW vs SW vs MS)

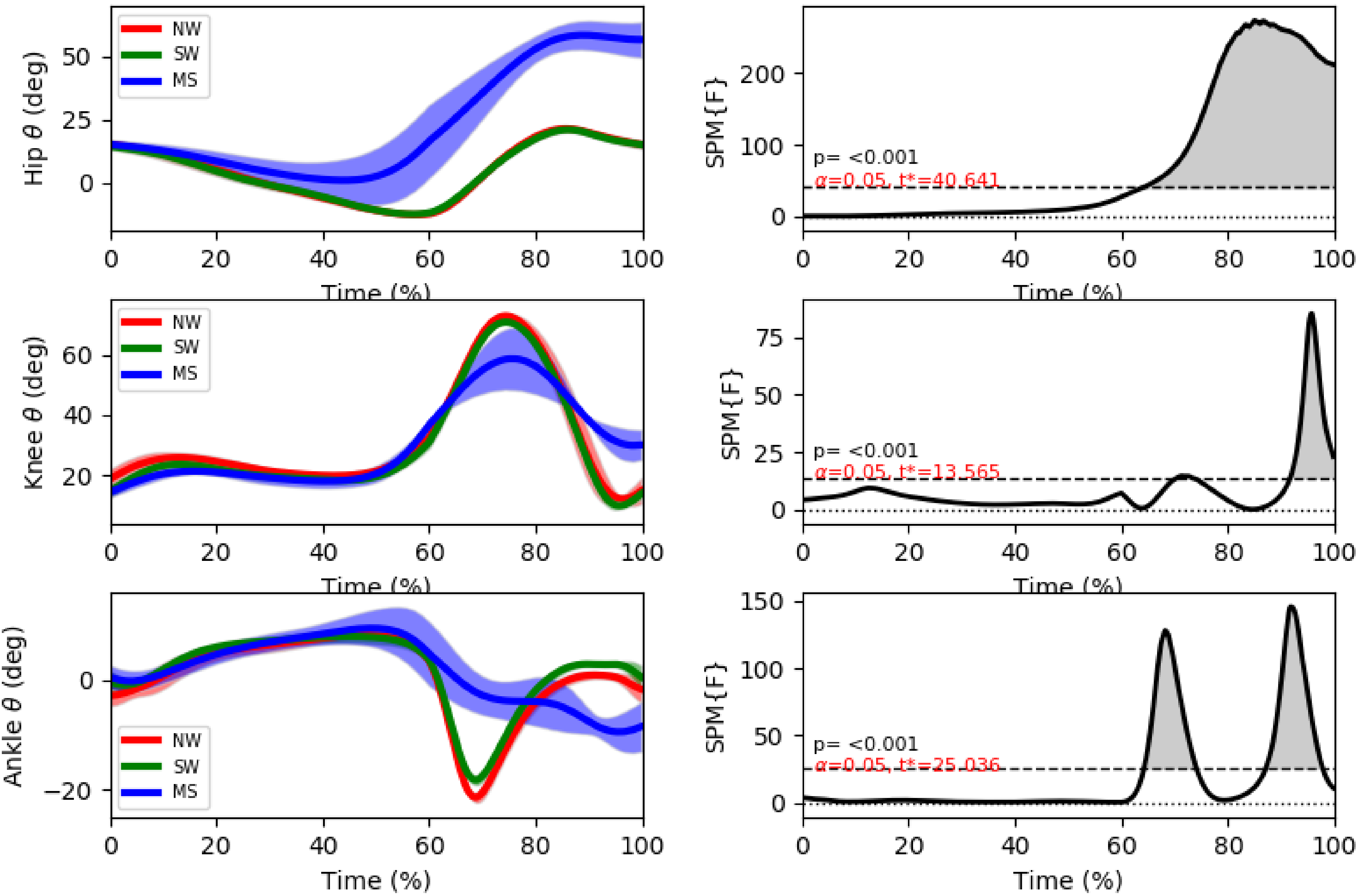

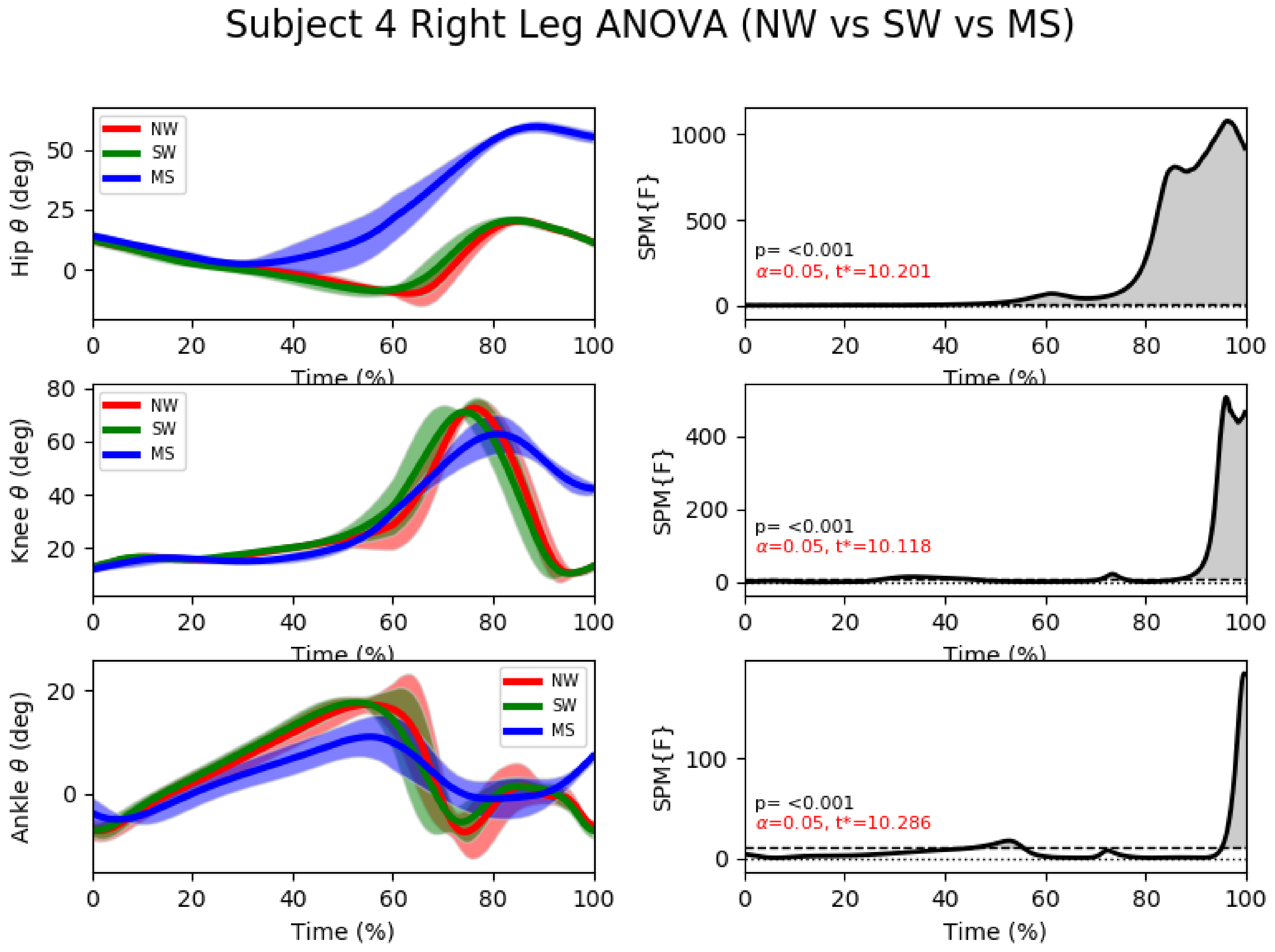

Subject 4 Right Leg ANOVA (NW vs SW vs MS)
NW
SW
MS
Hip θ (deg)
Time (%)
SPM{F}
p= <0.001
α=0.05, t*=10.201
Knee θ (deg)
α=0.05, t*=10.118
Ankle θ (deg)
α=0.05, t*=10.286

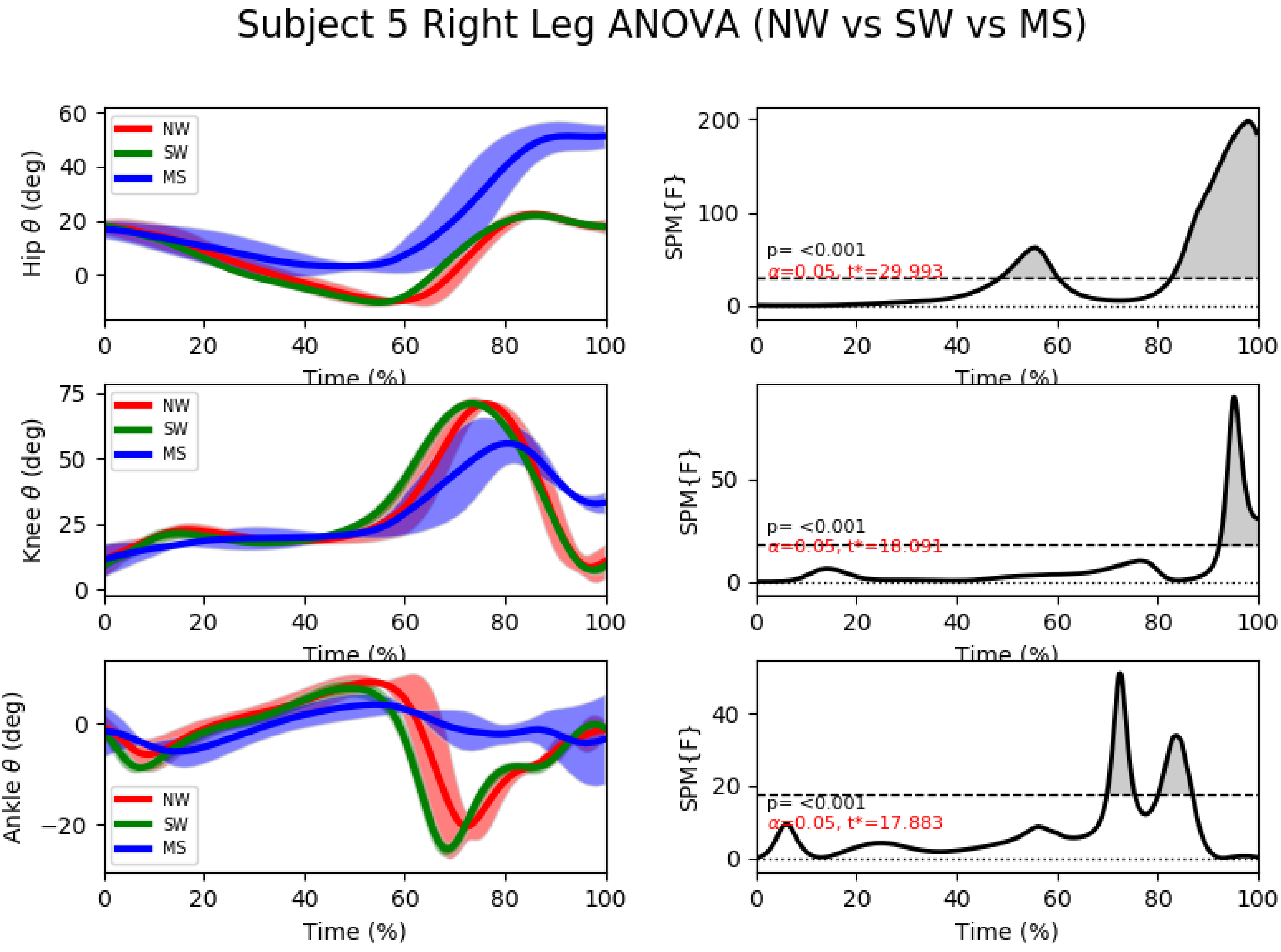

Subject 5 Right Leg ANOVA (NW vs SW vs MS)
Hip θ (deg)
NW
SW
MS
Time (%)
SPM{F}
p= <0.001
α=0.05, t*=29.993
Time (%)
Knee θ (deg)
NW
SW
MS
Time (%)
SPM{F}
p= <0.001
α=0.05, t*=18.091
Time (%)
Ankle θ (deg)
NW
SW
MS
Time (%)
SPM{F}
p= <0.001
α=0.05, t*=17.883
Time (%)

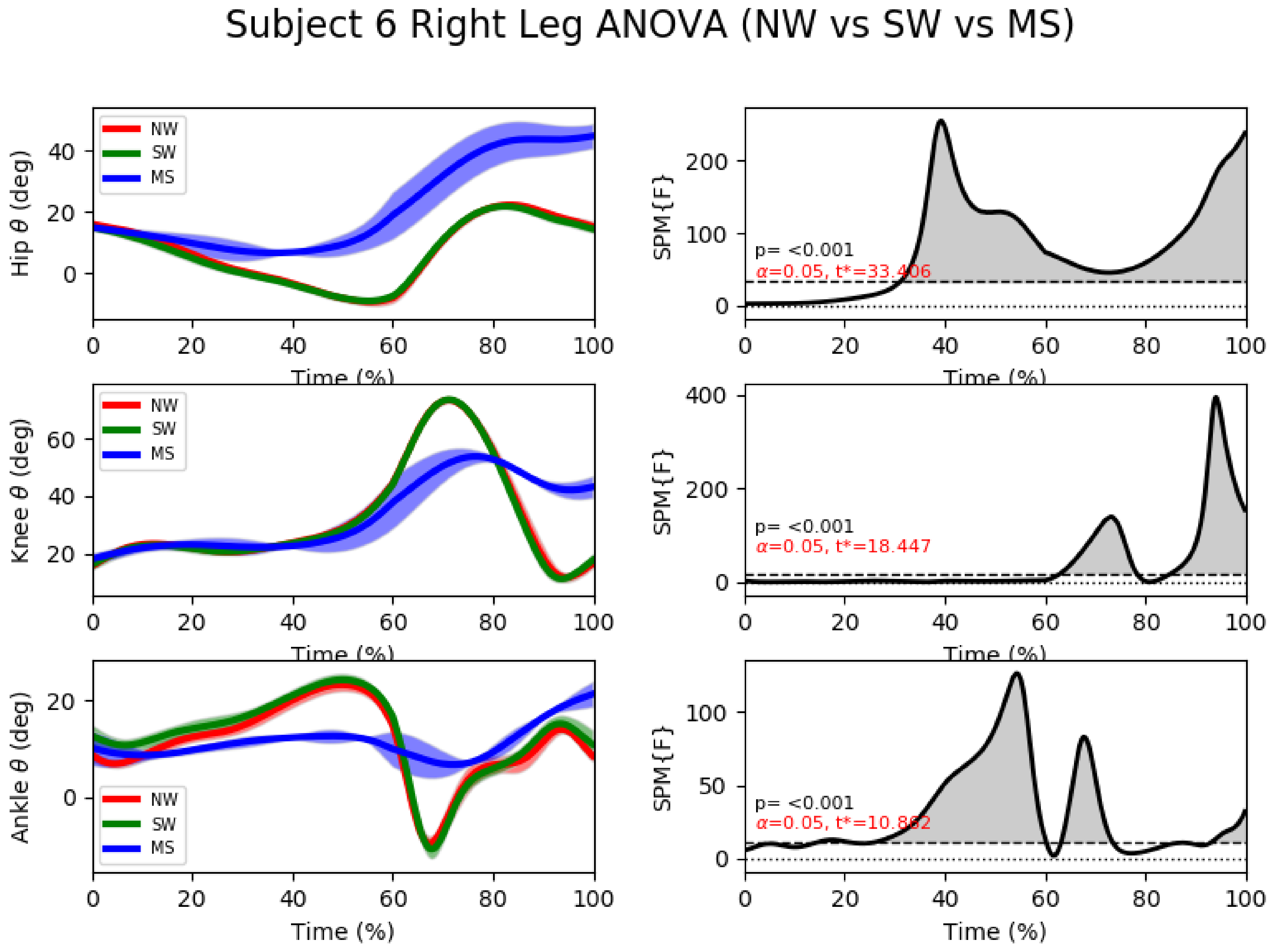
Subject 6 Right Leg ANOVA (NW vs SW vs MS)
NW
SW
MS
Hip θ (deg)
Time (%)
SPM{F}
p= <0.001
α=0.05, t*=33.406
Knee θ (deg)
α=0.05, t*=18.447
Ankle θ (deg)
α=0.05, t*=10.862

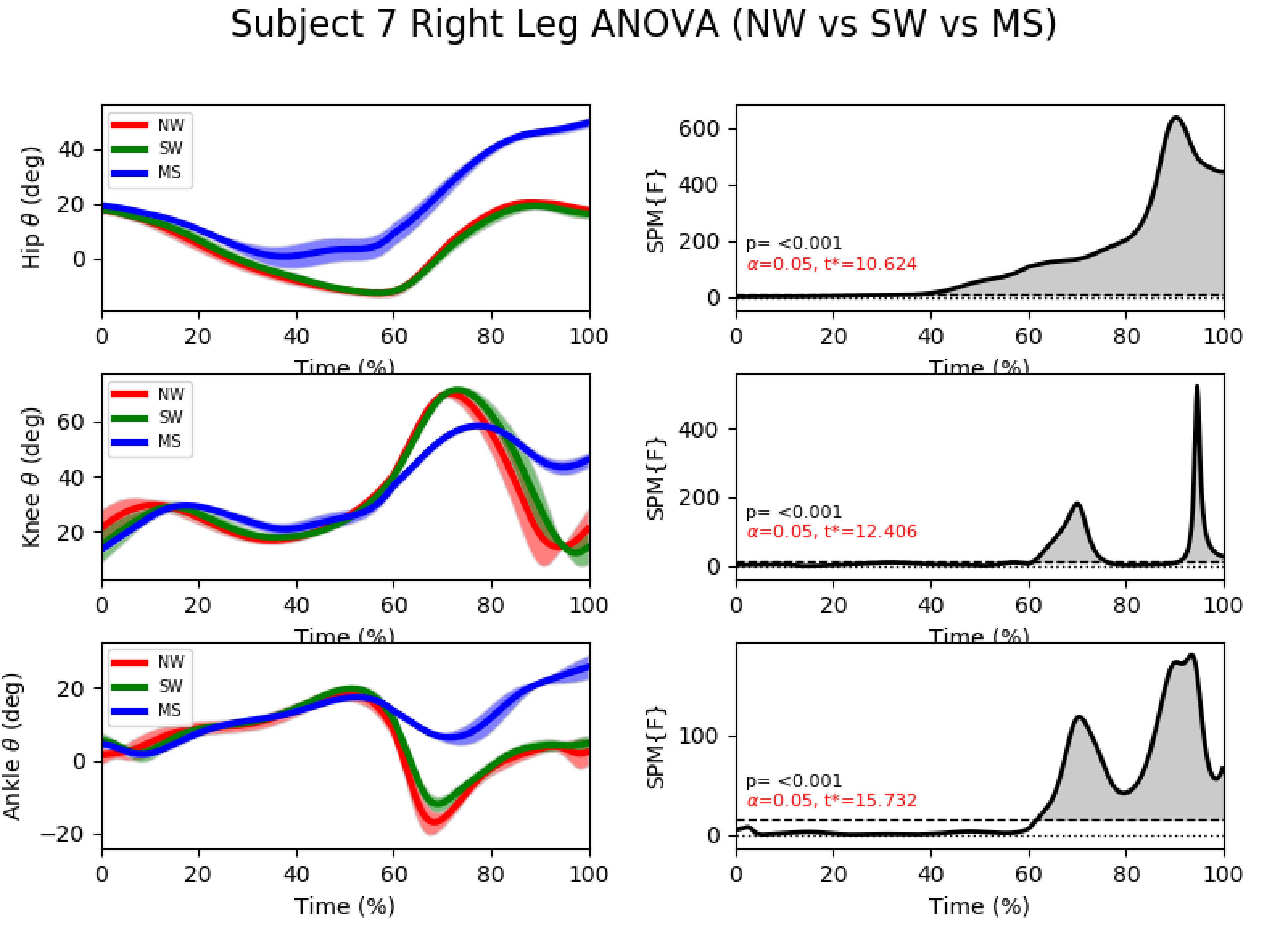
Subject 7 Right Leg ANOVA (NW vs SW vs MS)
NW
SW
MS
Hip θ (deg)
Knee θ (deg)
Ankle θ (deg)
Time (%)
SPM{F}
p= <0.001
α=0.05, t*=10.624
α=0.05, t*=12.406
α=0.05, t*=15.732

EarlyStance Slip (Left leg)

Subject 1 to 7

Comparing normal walking (NW) to strap walking (SW) to early stance slips on sliding sheet (SL)

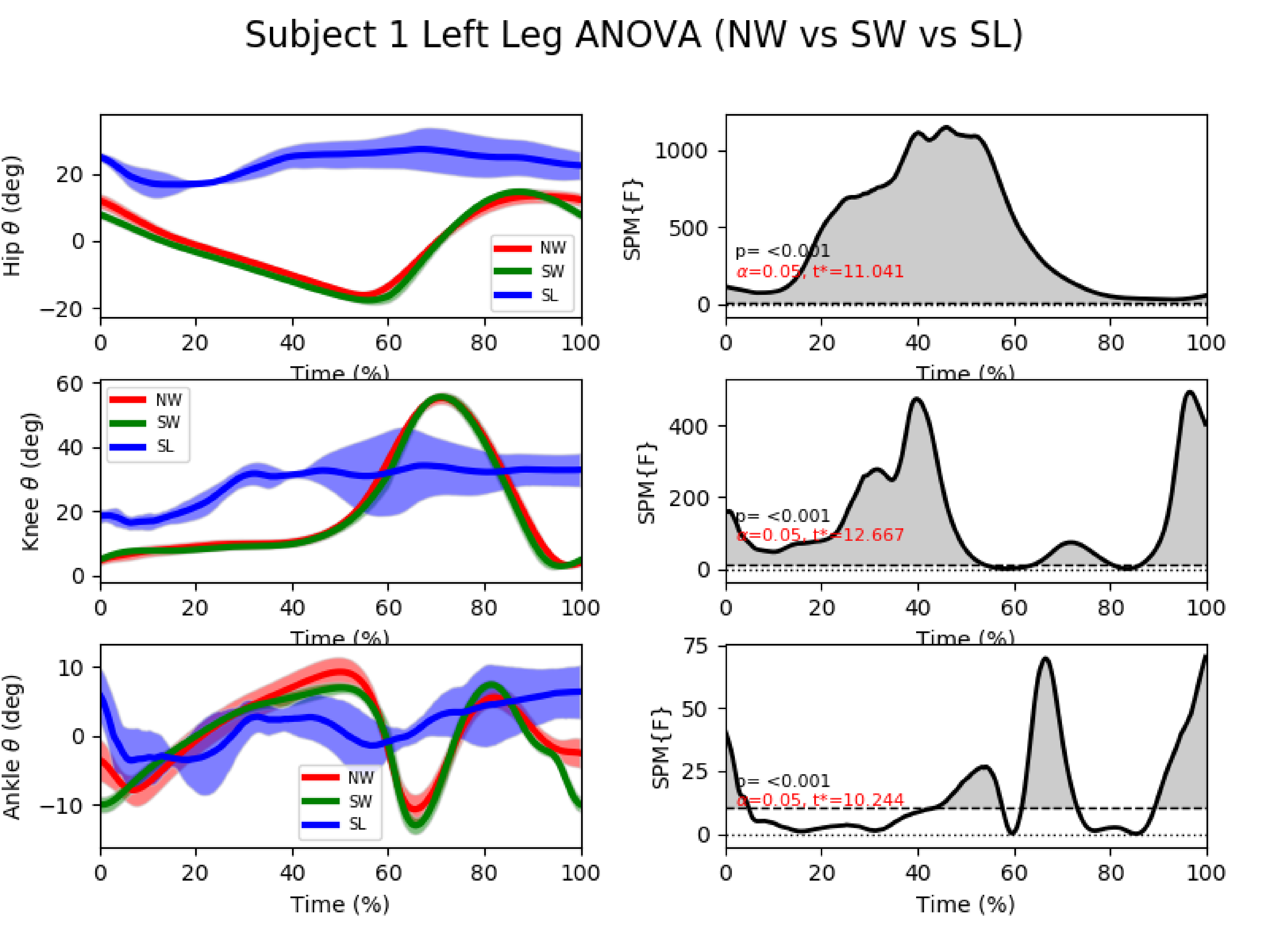

Subject 1 Left Leg ANOVA (NW vs SW vs SL)
Hip θ (deg)
Knee θ (deg)
Ankle θ (deg)
SPM{F}
Time (%)
NW
SW
SL
p= <0.001
α=0.05, t*=11.041
α=0.05, t*=12.667
α=0.05, t*=10.244

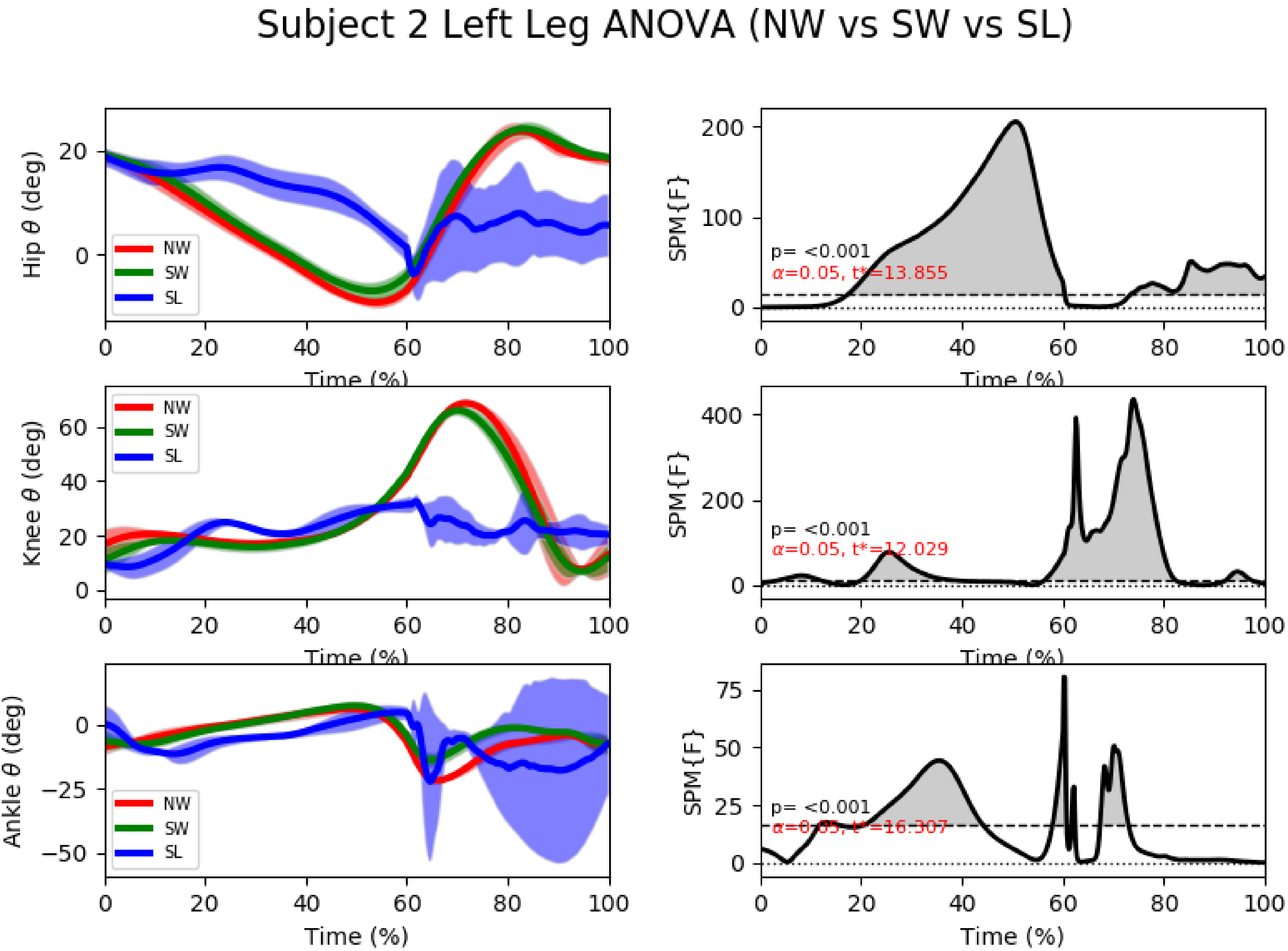
Subject 2 Left Leg ANOVA (NW vs SW vs SL)
Hip θ (deg)
NW
SW
SL
Time (%)
SPM{F}
p= <0.001
α=0.05, t*=13.855
Knee θ (deg)
α=0.05, t*=12.029
Ankle θ (deg)
α=0.05, t*=16.307

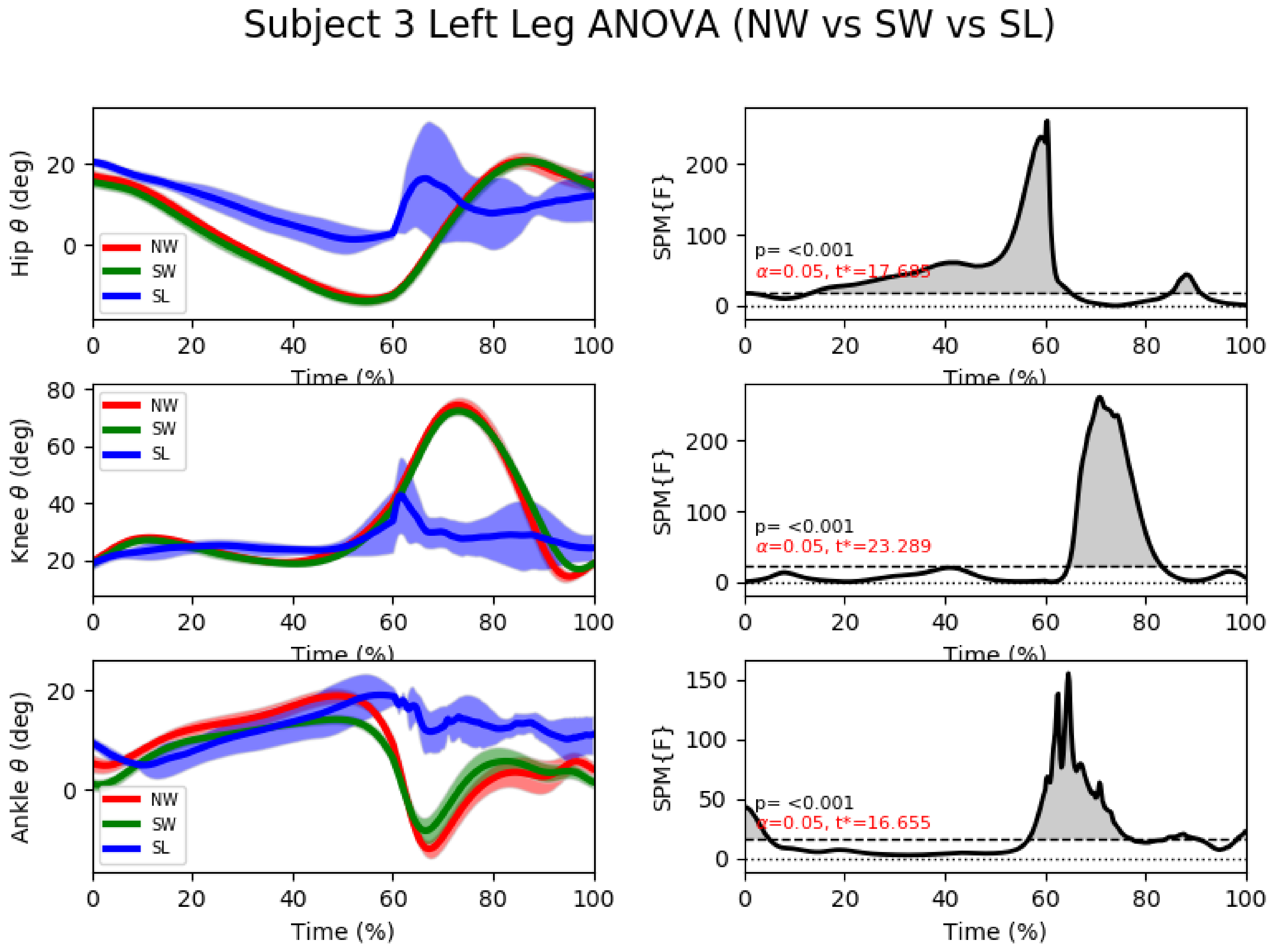

Subject 3 Left Leg ANOVA (NW vs SW vs SL)
Hip θ (deg)
Knee θ (deg)
Ankle θ (deg)
NW
SW
SL
Time (%)
SPM{F}
p= <0.001
α=0.05, t*=17.685
α=0.05, t*=23.289
α=0.05, t*=16.655

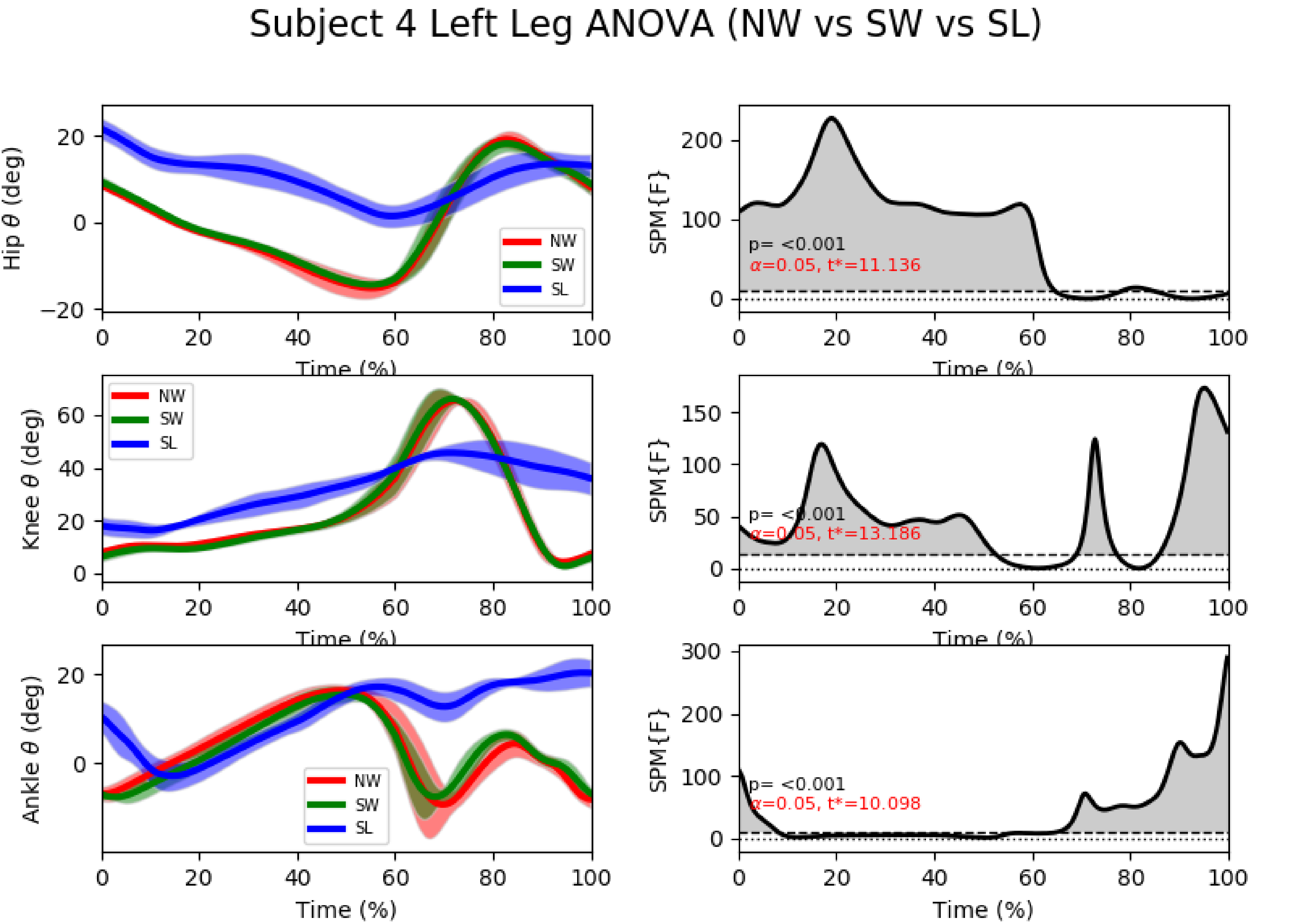

Subject 4 Left Leg ANOVA (NW vs SW vs SL)
Hip θ (deg)
Knee θ (deg)
Ankle θ (deg)
SPM{F}
Time (%)
NW
SW
SL
p= <0.001
α=0.05, t*=11.136
α=0.05, t*=13.186
α=0.05, t*=10.098

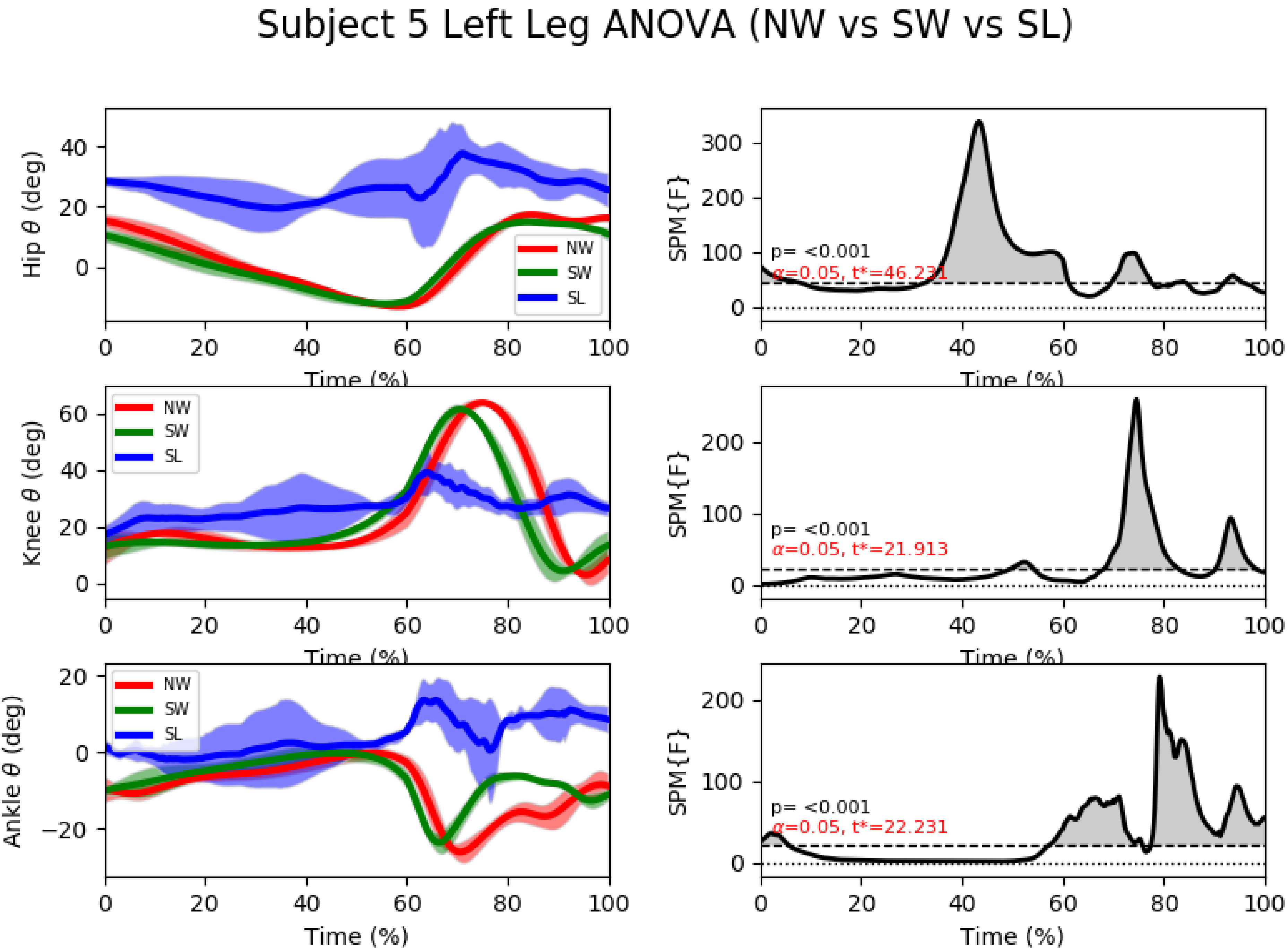

Subject 5 Left Leg ANOVA (NW vs SW vs SL)
Hip θ (deg)
Knee θ (deg)
Ankle θ (deg)
Time (%)
SPM{F}
NW
SW
SL
p= <0.001
α=0.05, t*=46.231
α=0.05, t*=21.913
α=0.05, t*=22.231

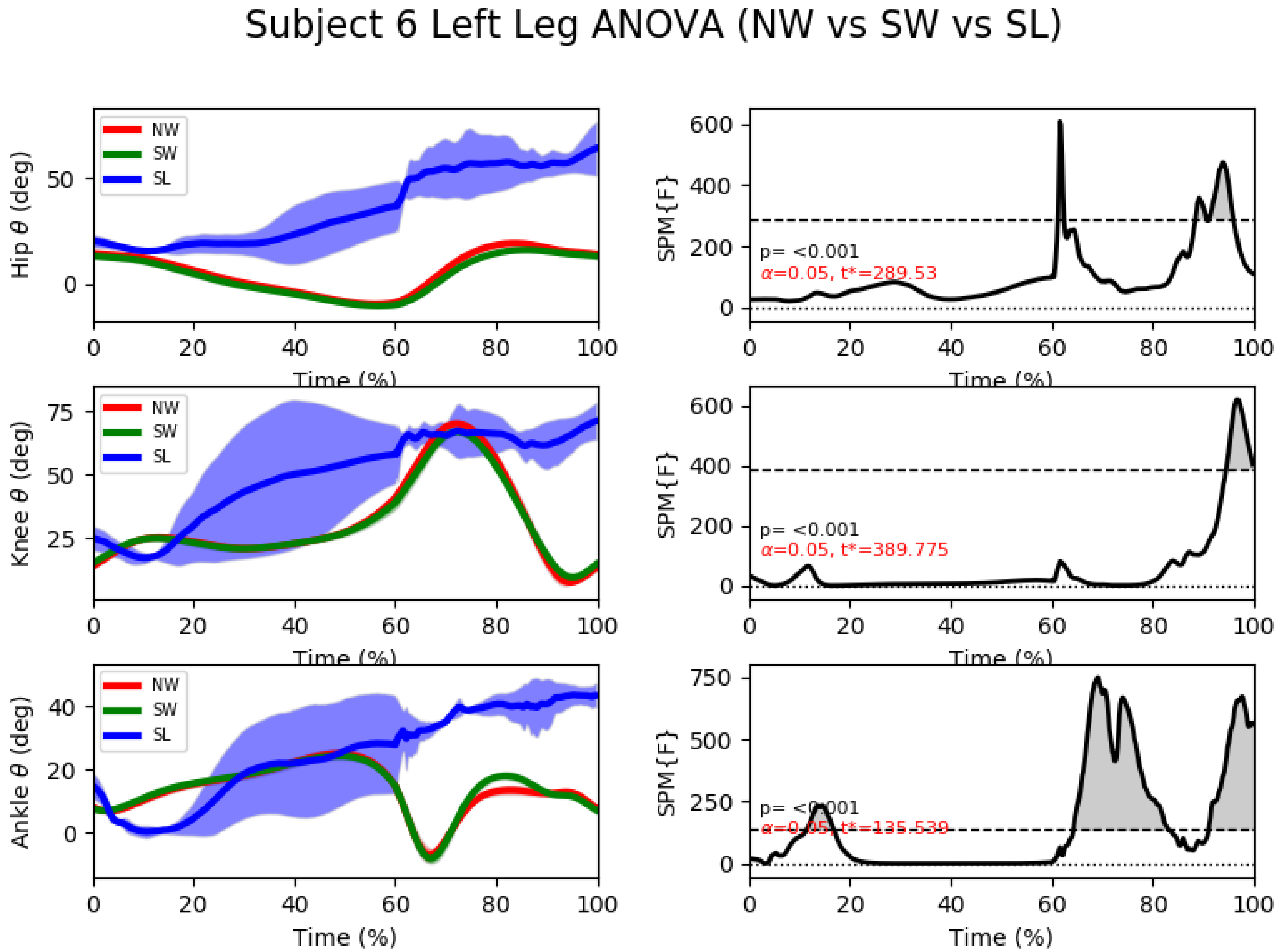
Subject 6 Left Leg ANOVA (NW vs SW vs SL)
NW
SW
SL
Hip θ (deg)
Knee θ (deg)
Ankle θ (deg)
Time (%)
SPM{F}
p= <0.001
α=0.05, t*=289.53
α=0.05, t*=389.775
α=0.05, t*=135.539

Subject 7 Left Leg ANOVA (NW vs SW vs SL)

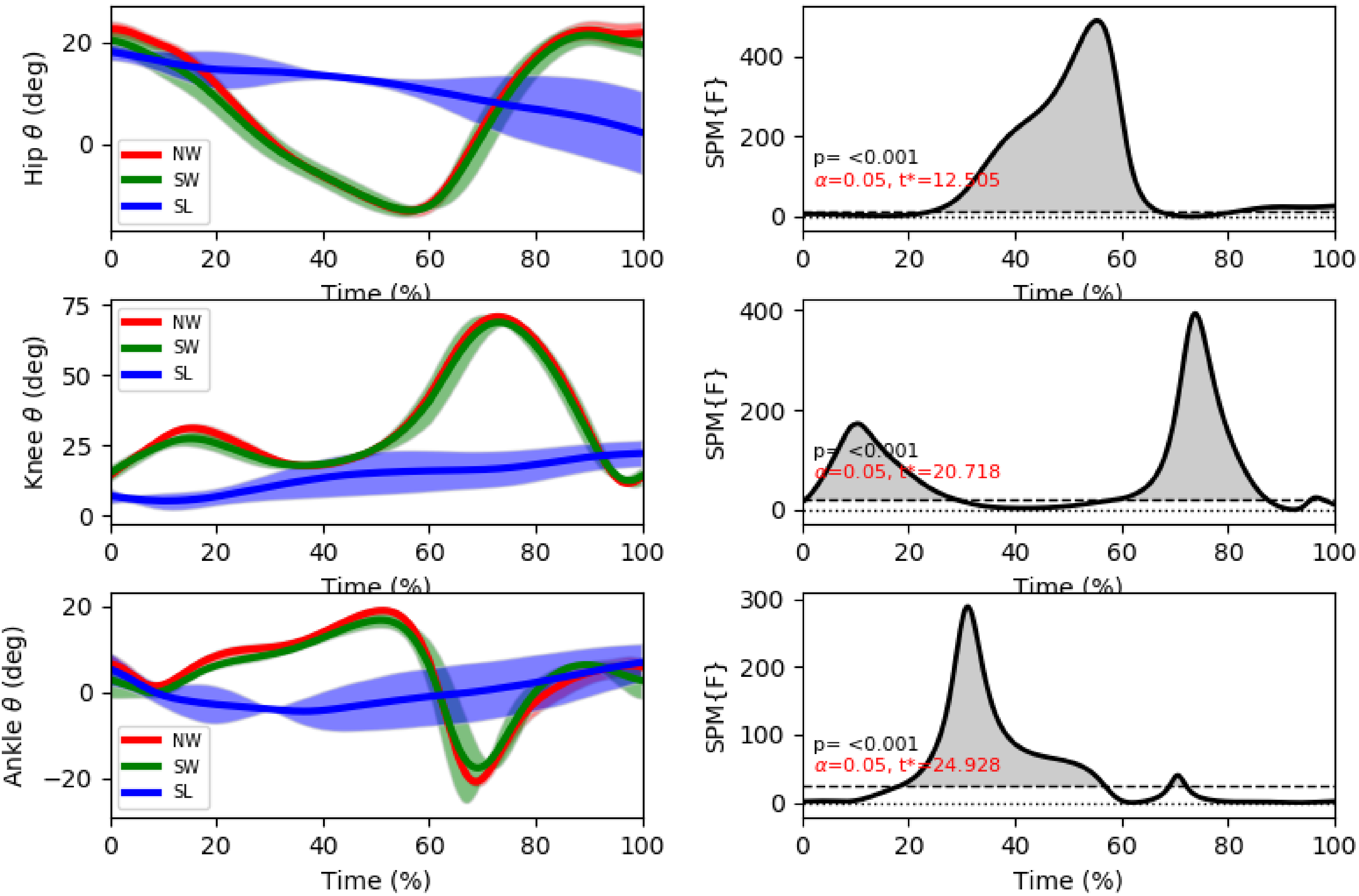

EarlyStance Slip (Right leg)

Subject 1 to 7

Comparing normal walking (NW) to strap walking (SW) to early stance slips on sliding sheet (SL)

Subject 1 Right Leg ANOVA (NW vs SW vs SL)

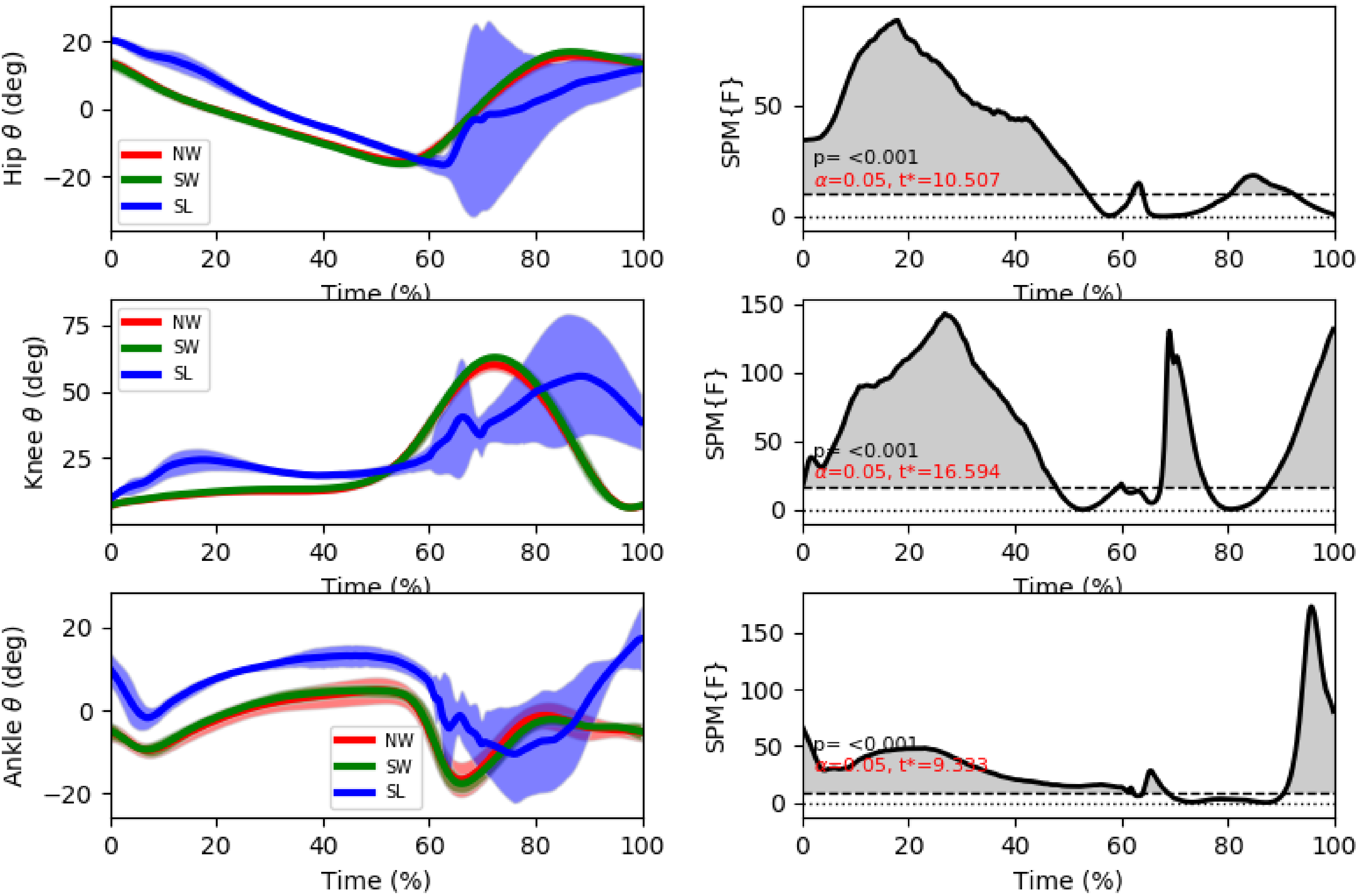

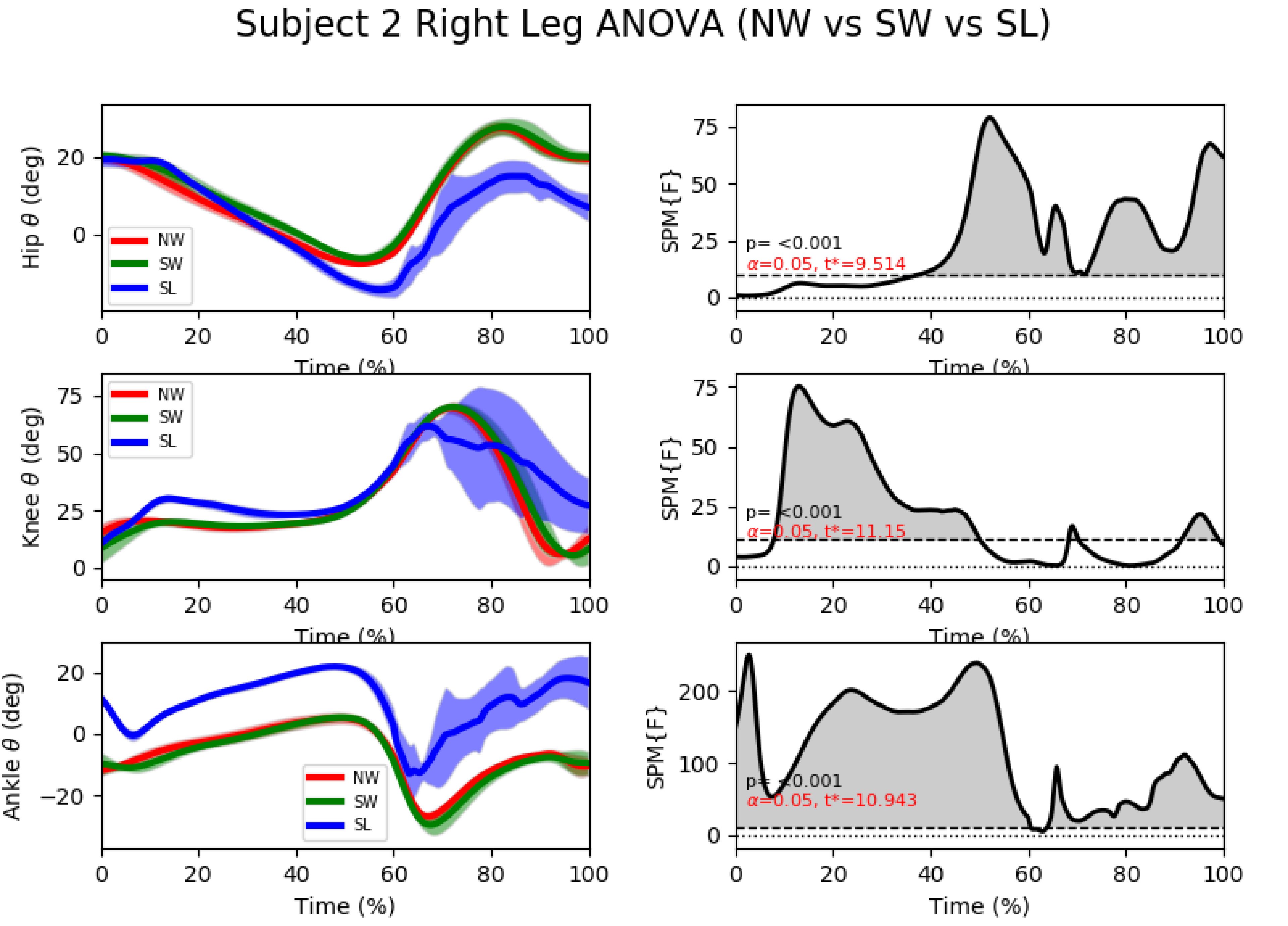

Subject 2 Right Leg ANOVA (NW vs SW vs SL)
Hip θ (deg)
NW
SW
SL
Time (%)
SPM{F}
p= <0.001
α=0.05, t*=9.514
Knee θ (deg)
α=0.05, t*=11.15
Ankle θ (deg)
α=0.05, t*=10.943

Subject 3 Right Leg ANOVA (NW vs SW vs SL)

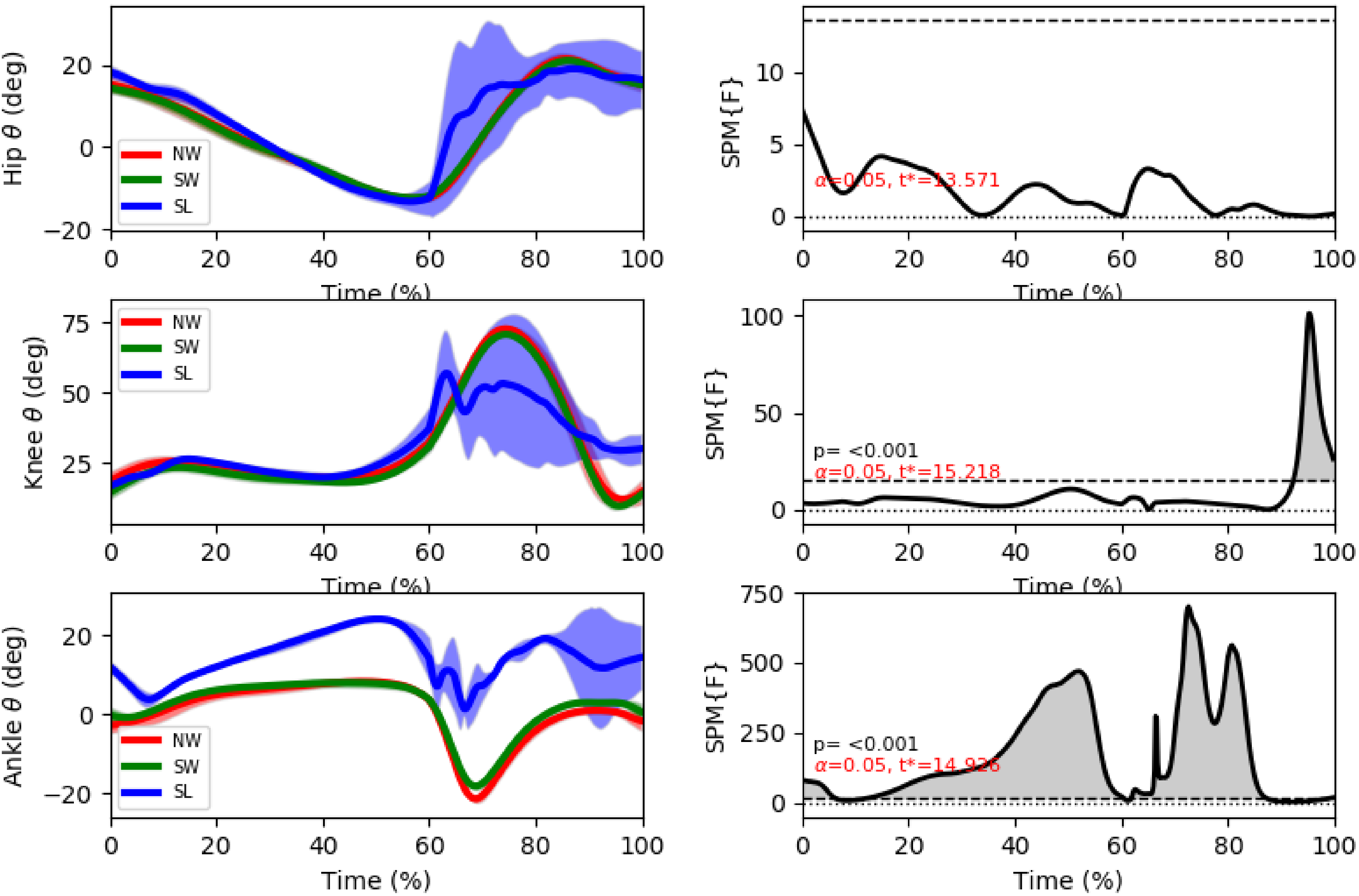

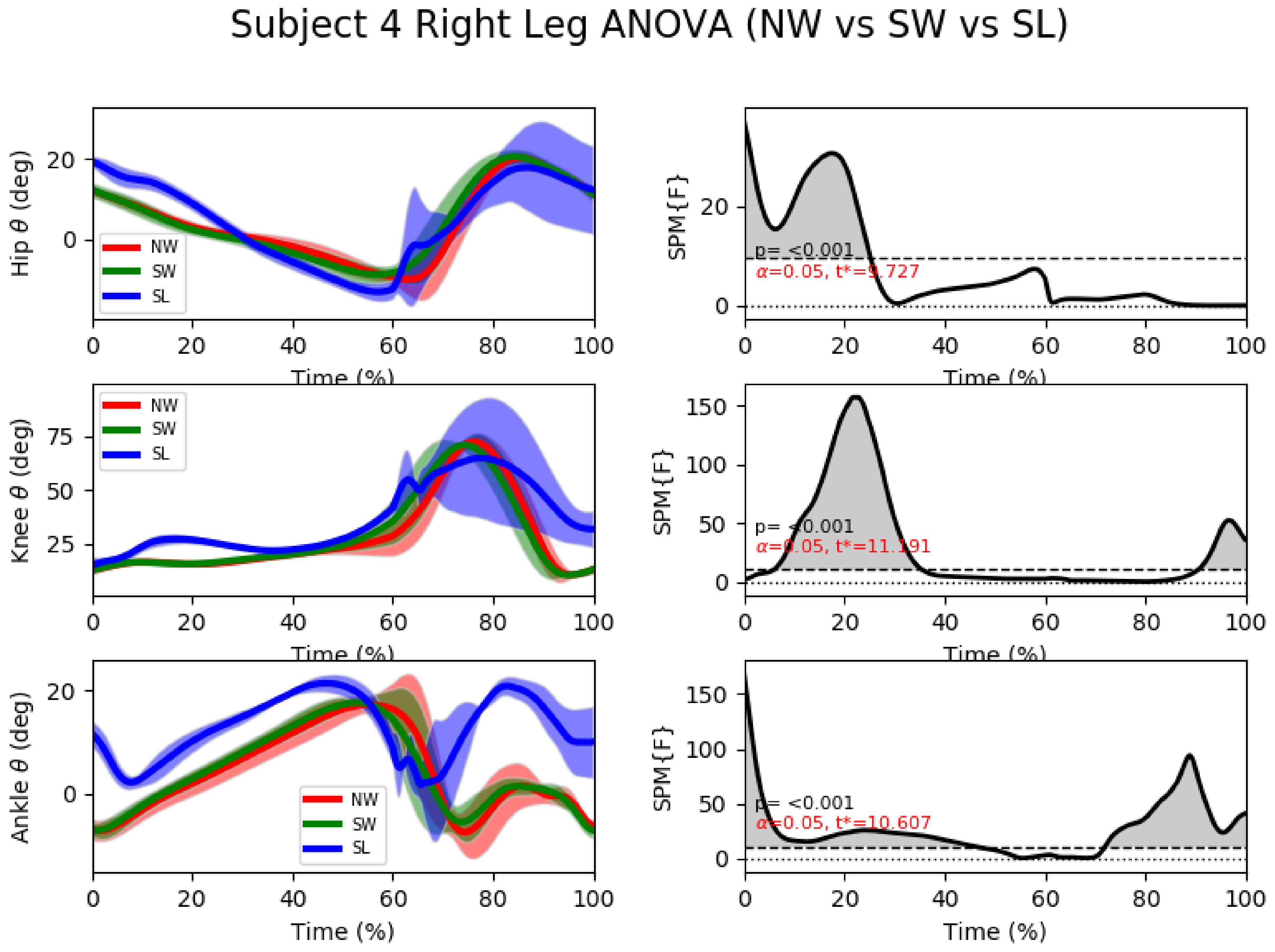
Subject 4 Right Leg ANOVA (NW vs SW vs SL)
Hip θ (deg)
Knee θ (deg)
Ankle θ (deg)
NW
SW
SL
SPM{F}
p= <0.001
α=0.05, t*=9.727
α=0.05, t*=11.191
α=0.05, t*=10.607
Time (%)

Subject 5 Right Leg ANOVA (NW vs SW vs SL)

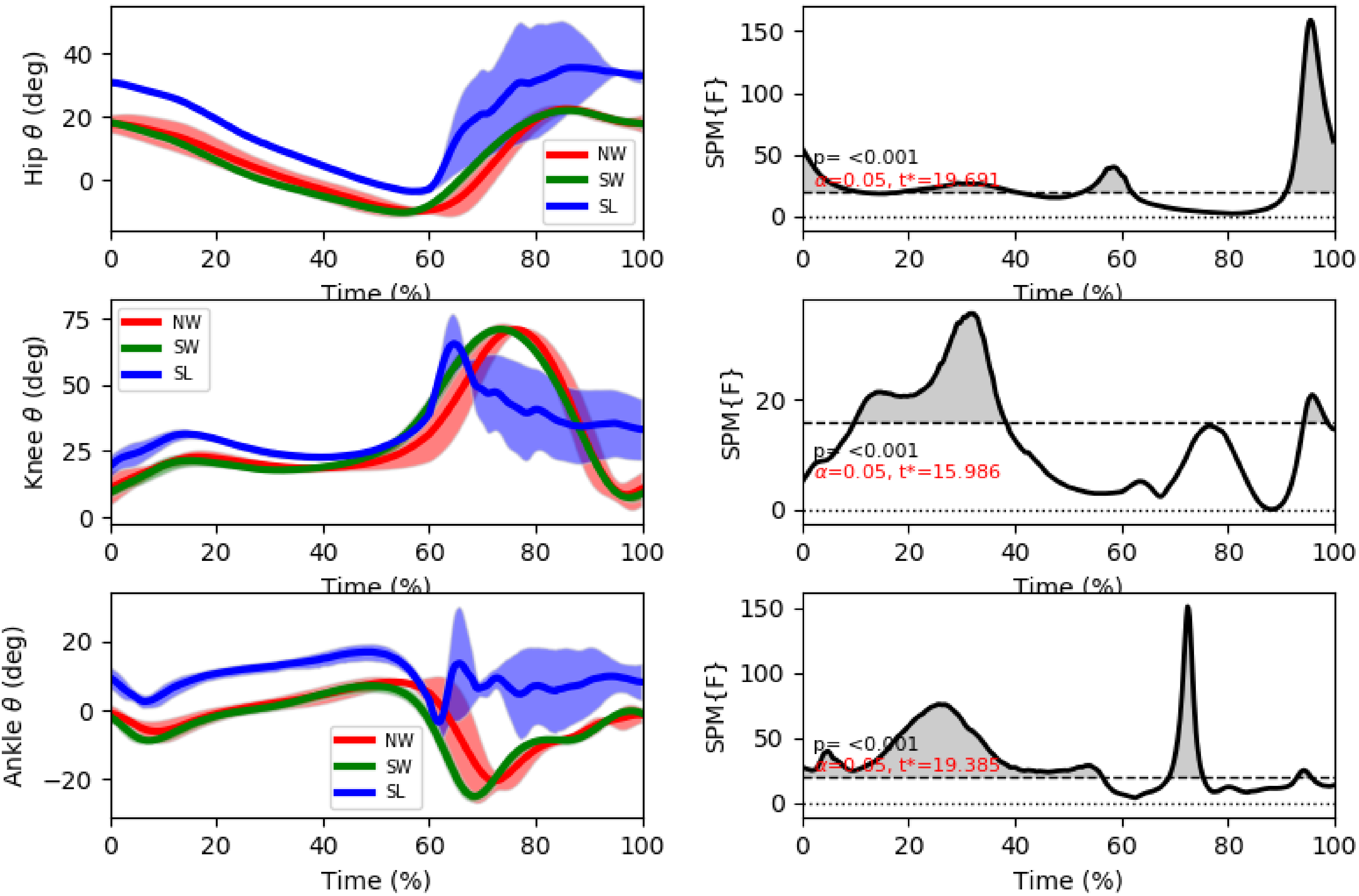

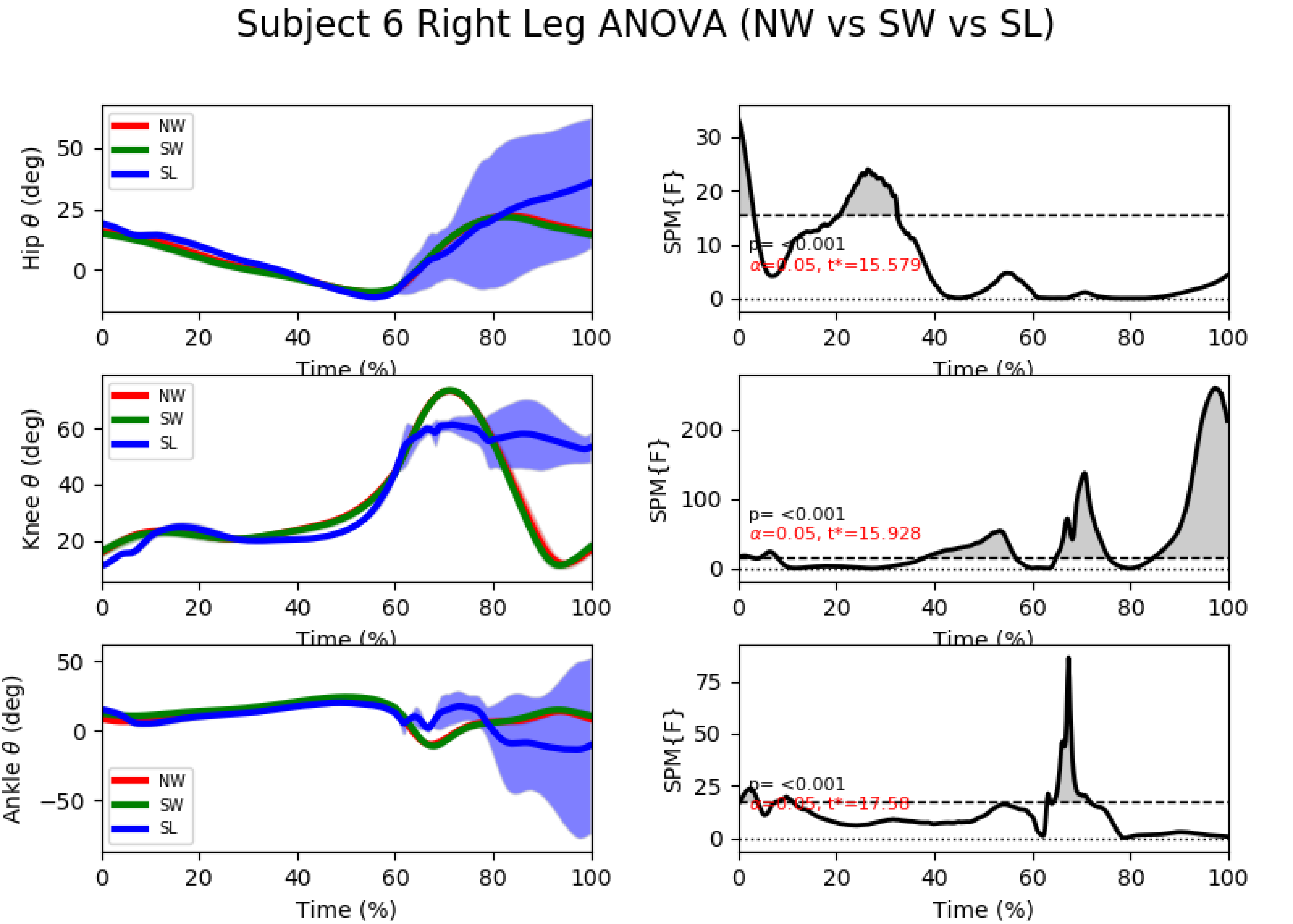

Subject 6 Right Leg ANOVA (NW vs SW vs SL)
NW
SW
SL
Hip θ (deg)
Time (%)
SPM{F}
p= <0.001
α=0.05, t*=15.579
Knee θ (deg)
p= <0.001
α=0.05, t*=15.928
Ankle θ (deg)
p= <0.001
α=0.05, t*=17.58

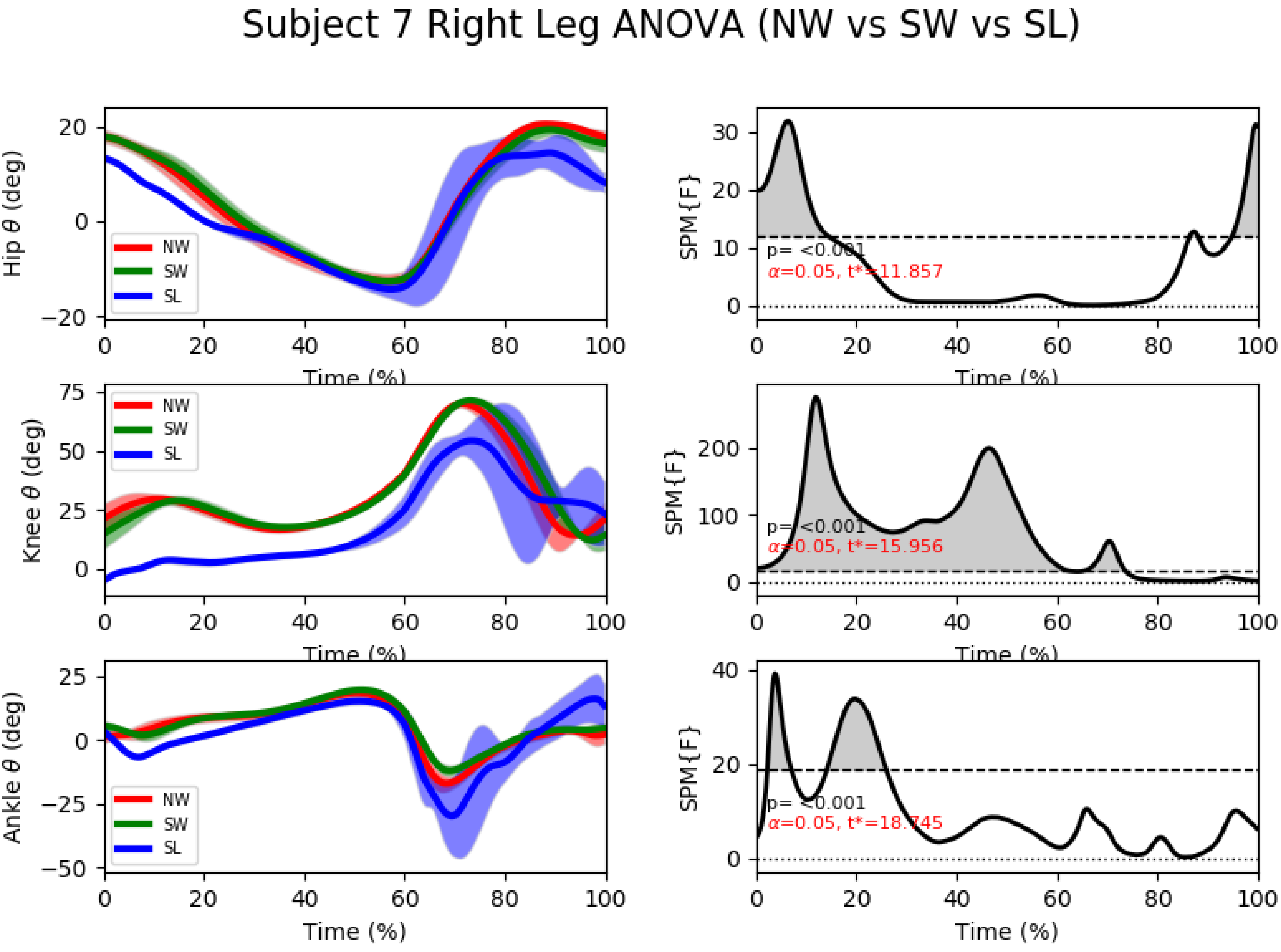

Subject 7 Right Leg ANOVA (NW vs SW vs SL)
Hip θ (deg)
Knee θ (deg)
Ankle θ (deg)
NW
SW
SL
SPM{F}
p= <0.001
α=0.05, t*=11.857
α=0.05, t*=15.956
α=0.05, t*=18.745
Time (%)
